# Gene Therapy of Dominant *CRX*-Leber Congenital Amaurosis using Patient Stem Cell-Derived Retinal Organoids

**DOI:** 10.1016/j.stemcr.2020.12.018

**Published:** 2021-01-28

**Authors:** Kamil Kruczek, Zepeng Qu, James Gentry, Benjamin R. Fadl, Linn Gieser, Suja Hiriyanna, Zachary Batz, Mugdha Samant, Ananya Samanta, Colin J. Chu, Laura Campello, Brian P. Brooks, Zhijian Wu, Anand Swaroop

**Affiliations:** 1 Neurobiology, Neurodegeneration and Repair Laboratory, National Eye Institute, National Institutes of Health, MSC0610, 6 Center Drive, Bethesda, MD 20892, USA; 2 Ocular Gene Therapy Core, National Eye Institute, National Institutes of Health, Bethesda, MD, USA; 3 Laboratory of Immune System Biology, National Institute of Allergy and Infectious Diseases, National Institutes of Health, Bethesda, MD, USA; 4 Ophthalmic Genetics and Visual Function Branch, National Eye Institute, National Institutes of Health, Bethesda, MD, USA

**Keywords:** pluripotent stem cells, iPSC, 3-D organoids, retinal degeneration, AAV, therapy, disease modeling, transcription factor, transcriptome, scRNA-seq

## Abstract

Mutations in the photoreceptor transcription factor gene cone-rod homeobox (CRX) lead to distinct retinopathy phenotypes, including early-onset vision impairment in dominant Leber congenital amaurosis (LCA). Using induced pluripotent stem cells (iPSCs) from a patient with *CRX*-I138fs48 mutation, we established an *in vitro* model of *CRX*-LCA in retinal organoids that showed defective photoreceptor maturation by histology and gene profiling, with diminished expression of visual opsins. Adeno-associated virus (AAV)-mediated *CRX* gene augmentation therapy partially restored photoreceptor phenotype and expression of phototransduction-related genes as determined by single-cell RNA-sequencing. Retinal organoids derived from iPSCs of a second dominant *CRX*-LCA patient carrying K88N mutation revealed the loss of opsin expression as a common phenotype, which was alleviated by AAV-mediated augmentation of CRX. Our studies provide a proof-of-concept for developing gene therapy of dominant *CRX*-LCA and other *CRX* retinopathies.

## Highlights

• Leber congenital amaurosis caused by *CRX* mutations is modeled in retinal organoids
• Patient iPSCs-derived organoids show impaired expression of visual opsins
• AAV-mediated *CRX* delivery partially restores expression of phototransduction genes
• Gene therapy is applicable to mutations in DNA-binding and transactivation domains

## Graphical Abstract

**Figure:**
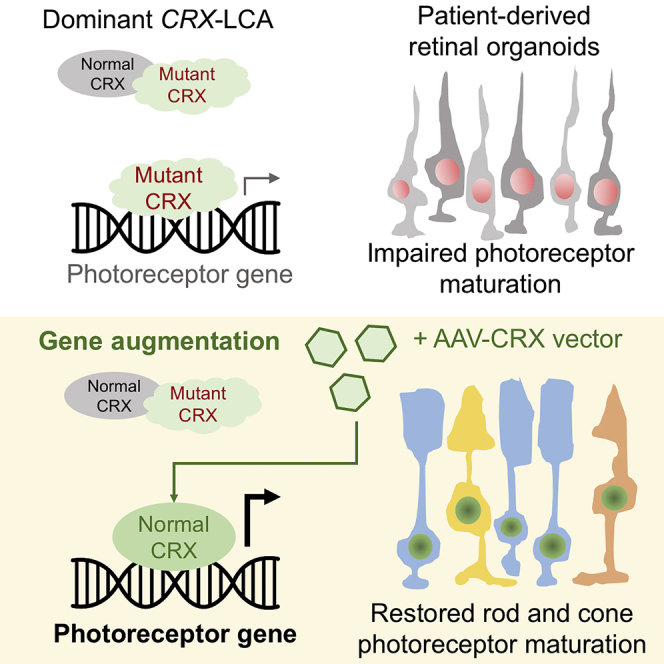

## Teaser

In this article, Swaroop and colleagues show impaired expression of opsin visual pigments in retinal organoids derived from iPSC lines of patients with Leber congenital amaurosis, caused by dominant mutations in the photoreceptor transcription factor gene *CRX*. AAV-mediated *CRX* gene augmentation partially restores the expression of phototransduction genes, suggesting a potential therapeutic strategy.

## Introduction

Leber congenital amaurosis (LCA) constitutes a group of rare early-onset retinal dystrophies with severe clinical manifestations, resulting in vision loss during infancy (den Hollander et al., 2008). LCA is genetically heterogeneous with mutations identified in at least 25 genes; a vast majority of these are crucial for photoreceptor development and/or function (Kumaran et al., 2017). Most patients with LCA exhibit autosomal recessive inheritance. Mouse models have been valuable for elucidating disease etiology (Veleri et al., 2015) and for preclinical therapy development, leading to the first U.S. Food and Drug Administration-approved adeno-associated virus (AAV)-based gene therapy of recessive LCA caused by *RPE65* mutations (Apte, 2018). However, it is widely recognized that available animal models do not fully capture complexities of human disease, slowing further clinical translation. No treatment currently exists for other, especially dominant, forms of LCA.

Cone-rod homeobox protein CRX is essential for development of photoreceptors in the retina (Furukawa et al., 1999). CRX controls expression of most rod and cone photoreceptor genes through its interaction with bZIP transcription factor NRL (Mitton et al., 2000) and/or an intricate network of regulatory proteins (Hennig et al., 2008). Heterozygous mutations in *CRX* cause early-onset retinal dystrophies with extensive phenotypic heterogeneity, with rare reports of biallelic variants in LCA (Huang et al., 2012; Hull et al., 2014; Ibrahim et al., 2018; Rivolta et al., 2001; Swaroop et al., 1999). Notably, a majority of reported dominant LCA can be attributed to *CRX*. Disease-causing *CRX* mutations can either reduce the transcriptional activity or impart a gain of function effect (Tran et al., 2014). Studies in animal models revealed a severe phenotype associated with dominant frameshift mutations in *Crx* compared with loss-of-function alleles (Roger et al., 2014; Tran et al., 2014).

We previously described clinical presentations of two pediatric patients carrying c.G264T (p.K88N)- or c.413delT (p.I138fs48) dominant *CRX* mutations manifested as LCA, with a complete loss of light-evoked responses in electroretinogram recordings (Nichols et al., 2010). Importantly, retinal imaging revealed preservation of outer nuclear layer, suggesting presence of viable but nonfunctional photoreceptors, which could be potential targets for gene therapy. Molecular analysis of mutant alleles demonstrated differential interaction with NRL *in vitro*, suggesting interference with gene regulation in rod photoreceptors (Nichols et al., 2010). Although animal models phenocopy human *CRX* retinopathies (Roger et al., 2014; Tran et al., 2014) and can provide proof-of-concept for treatment (Watanabe et al., 2013), there are significant differences in development, cell-type composition, and molecular profiles of human retina (Hoshino et al., 2017). Therefore, we decided to take advantage of patient-derived induced pluripotent stem cells (iPSCs) to create a human model for developing therapeutic paradigms for *CRX*-LCA.

Directed differentiation of iPSCs into three-dimensional (3D) retinal organoids has enabled the modeling of retinopathies in patient-specific genetic background (Kruczek and Swaroop, 2020). Next generation sequencing technologies have provided a detailed comparison of developing retinal organoids with human retina (Collin et al., 2019; Cowan et al., 2020; Hoshino et al., 2017; Kaya et al., 2019; Kim et al., 2019) and permit evaluation of major retinal cell types in a disease context. In this study, we established a retinal organoid model of *CRX*-LCA from patient-derived iPSCs and demonstrate perturbation in molecular phenotype of photoreceptors, including diminished expression of visual opsins, in concordance with clinically observed loss of light responses. We also show partial restoration of both rod and cone gene expression by delivering correct CRX transgene driven by a human *CRX* promoter via an AAV vector. Our studies provide a path forward for treatment of dominant *CRX*-LCA by gene augmentation.

## Results

**Figure 1:**
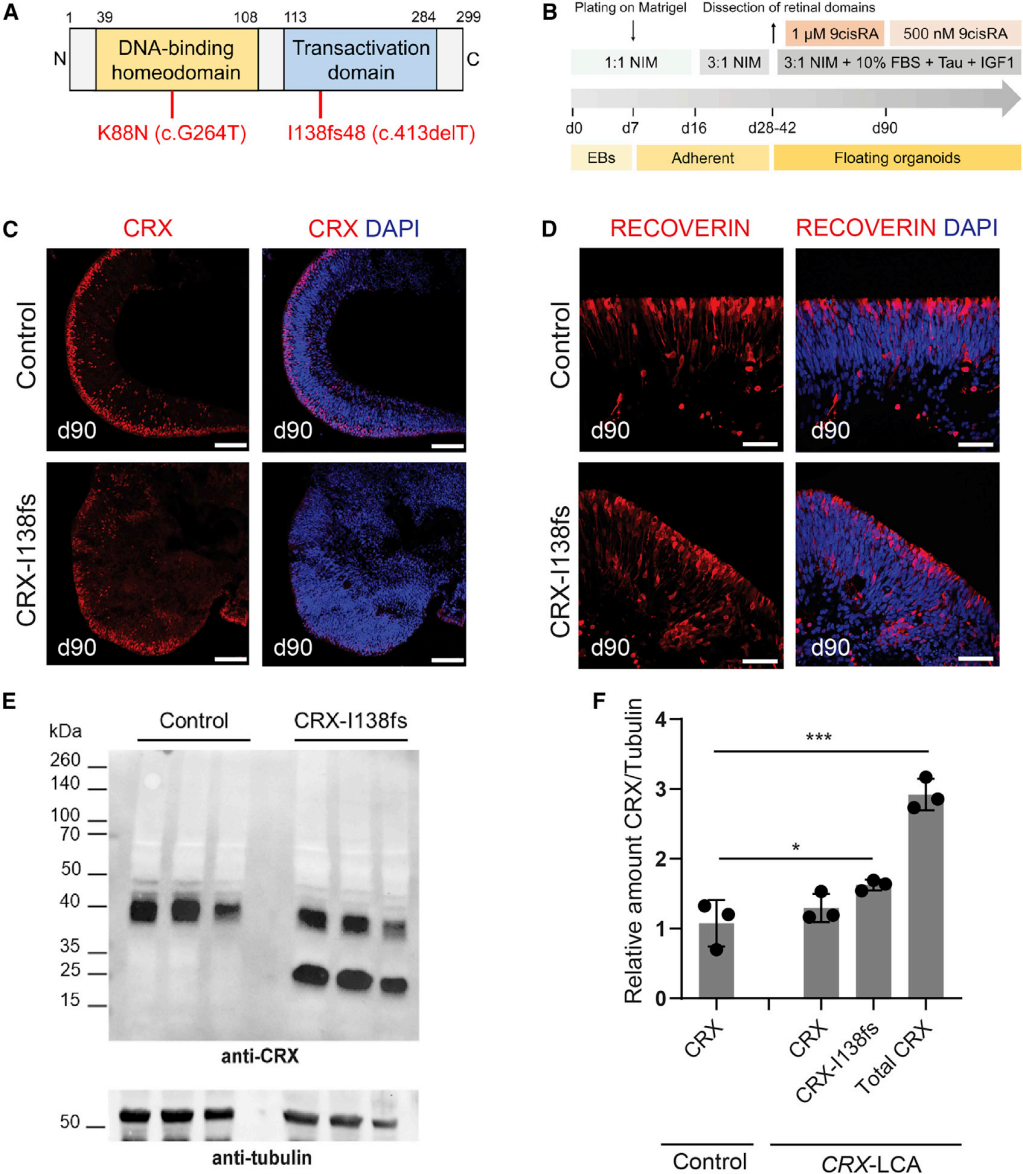
Differentiation of Photoreceptors in CRX-I138fs Retinal Organoids (A) Schematic representation of CRX protein, showing domain structure and the positions of dominant pathological mutations in the study patients. Number of amino acid residue is indicated over the bar. N and C indicate amino- and carboxyl-terminal of the CRX protein. (B) An overview of the retinal organoid differentiation protocol. (C) CRX expression in developing photoreceptors at day 90. CRX antibody labeled apically aligning developing photoreceptors in both control and patient organoids. Scale bar, 50 μm. Note a more diffuse pattern in the patient sample. Nuclei were stained with DAPI (4′,6-diamidino-2-phenylindole). (D) Developing photoreceptors of both genotypes also express the marker protein Recoverin. Scale bar, 20 μm. (E) Immunoblot analysis of organoid protein extracts at day 90. Molecular mass markers (kDa) are indicated on the left. Protein samples from control and patient stem cell-derived organoids (n = 3 biological replicates containing three organoids each) were run in separate lanes. Tubulin was used as a loading control. A smaller molecular weight band, which corresponds to the mutant CRX-I138fs48 isoform, is evident in the patient samples. (F) Densitometry quantification of CRX protein bands normalized to the Tubulin loading control. Total CRX protein is significantly more abundant in CRX-I138fs patient samples. Mean ± SD plotted, ^∗^p < 0.05, ^∗∗∗^p < 0.001, one-way ANOVA.

Skin biopsies of two LCA patients carrying dominant c.G264T (p.K88N) or c.413delT (p.I138fs48) mutation in the *CRX* gene (Figure 1A), as well as unaffected familial controls (Table S1), were used to derive iPSC lines, which demonstrated normal karyotype and typical features of stem cells (Figure S1A, S1B, S5A, and S5B; Table S2). Retinal organoids were differentiated using a previously published protocol (Kaya et al., 2019; Zhong et al., 2014) (Figure 1B). Pairwise comparisons of organoids derived from each patient to respective healthy familial control were performed in all presented data. Both patient and control cell lines formed morphologically similar retinal neural epithelia (Figures S1C and S5C).

**Figure 2:**
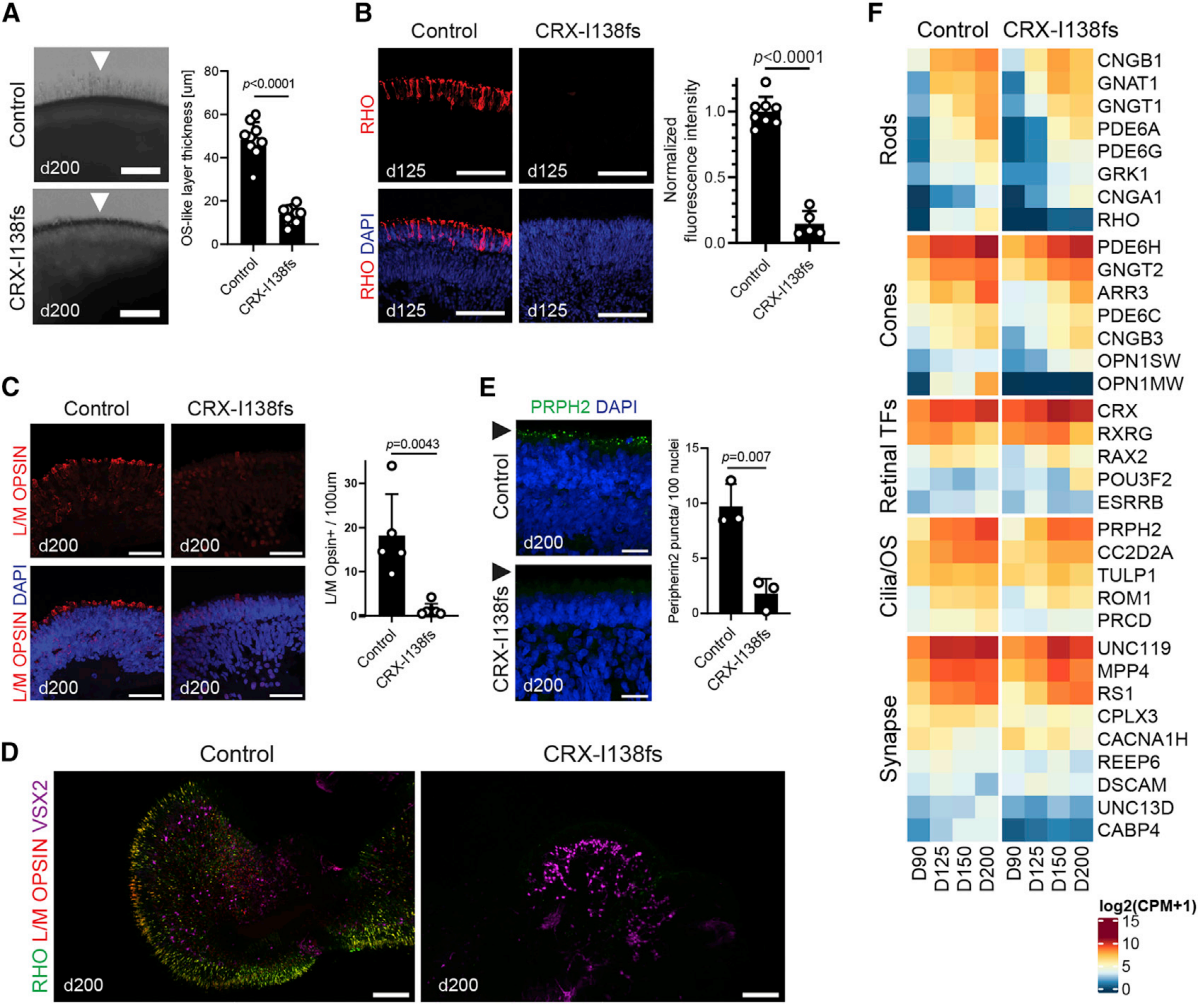
Impaired Photoreceptor Maturation in *CRX*-LCA Retinal Organoids (A) Brightfield images of apical aspect of organoid neural retina. Scale bar, 400 μm. Note loss of brush-like outer segment (OS) structures in patient (arrowheads). (B) Rhodopsin (RHO) immunostaining at day 125 and fluorescence intensity quantification. Nuclei were stained with DAPI (4′,6-diamidino-2-phenylindole). Scale bar, 20 μm. (C) L/M Opsin immunostaining with quantification of positive cells. Scale bar, 20 μm. (D) Representative whole-mount confocal images of organoids at day 200 immunostained for RHO, L/M Opsin, and Visual System Homeobox 2 (VSX2). Scale bar, 160 μm. (E) Peripherin2 (PRPH2) staining and puncta quantification. Scale bar, 10 μm. (F) Heatmap comparing expression of genes (bulk RNA-seq) across organoid development (days 90, 125, 150, and 200). Values are shown as log2(CPM+1). TFs, transcription factors; OS, outer segment. Number of organoids per genotype analyzed for quantifications: (A) control n = 10, patient n = 10; (B) control n = 8, patient n = 5; (C) n = 5 control, n = 3 patient; (E) control n = 3, patient n = 3. Values represent mean ± SD with individual data points plotted (three sections per organoid). Statistical significance was determined by Student’s t test; p values indicated.

Given that a frameshift mutation showed a particularly severe retinal phenotype in *Crx*^*Rip*^ mouse model (Roger et al., 2014), we first examined the organoids derived from c.413delT(p.I138fs48) iPSCs. Retinal organoids exhibit three stereotypical stages of differentiation (Capowski et al., 2019; Kaya et al., 2019) corresponding to developmental epochs in human fetal retina (Hoshino et al., 2017). At stage 1, neuroepithelia of control and patient organoids were indistinguishable (Figures S1C–S1E). During stage 2, CRX-expressing photoreceptors aligned at the apical side of neural retina in both control and patient organoids (Figure 1C) and stained positive for early photoreceptor marker, Recoverin (Figure 1D). Immunoblotting of day 90 organoid protein extracts revealed a truncated CRX protein (referred as I138fs) in patient samples (Figure 1E). Quantification of the protein bands indicated higher levels of total CRX in patient samples with a significant proportion contributed by the mutant allele (Figure 1F). The phenotypic distinction between the control and patient organoids was evident at stage 3, when photoreceptors began to form outer segment-like structures visible as an apical “brush” in light microscopy. While clearly detectable in control organoids, formation of these structures was abrogated in CRX-I138fs patient organoids (Figure 2A; details of sampling size used for quantifications and description of statistical analyses are provided in methods and figure legends). As early as day 125, control organoids showed patches of developing rods as revealed by immunostaining of the rod visual pigment Rhodopsin, a transcriptional target of CRX. However, Rhodopsin staining was virtually absent in CRX-I138fs organoids (Figure 2B). Similarly, cone photoreceptor opsins displayed altered expression. S Opsin immunostaining was delayed compared with the control (Figures S2A–S2F), whereas the number of L/M Opsin + cones was substantially reduced (Figure 2C). 3D renderings of organoids at day 200 confirmed the diminished opsin staining (Figure 2D, Videos S1 and S2). Another outer segment component, Peripherin2, was also dramatically reduced in CRX-I138fs organoids (Figure 2E). In contrast, developing ribbon synapses appeared unaffected (Figures S2G and S2H). Transcriptome analysis at key stages of organoid differentiation, days 90, 125, 150, and 200, identified differential expression of multiple transcripts associated with photoreceptor function (Figure 2F), consistent with the established role of CRX (Furukawa et al., 1999; Hennig et al., 2008). Rod and L/M cone opsins (*RHO*, *OPN1MW*) were the most significantly diminished transcripts in CRX-I138fs organoids (Figure 2F), in accordance with immunostaining results (Figures 2B and 2C). Thus, histological and transcriptome analyses of retinal organoids showed aberrant photoreceptor development due to the I138fs mutation in *CRX*.

Video S1. Control retinal organoid wholemounts were cleared and immunostaining of Rhodopsin, L/M Opsin and VSX2 performed in the whole tissue, related to Figure 1 Imaging using confocal microscopy was followed by 3D reconstruction using Imaris software. Scale bar, 160 μm.

Video S2. CRX-I138fs patient retinal organoid wholemounts were cleared and immunostaining of Rhodopsin, L/M Opsin, and VSX2 performed as in SV1, related to Figure 1 3D reconstruction using Imaris software revealed near complete loss of Rhodopsin and significant reduction in L/M Opsin signal compared with healthy control. In contrast to the opsins, VSX2, a marker of bipolar cells, was present in organoids of both genotypes. Scale bar, 160 μm.

**Figure 3:**
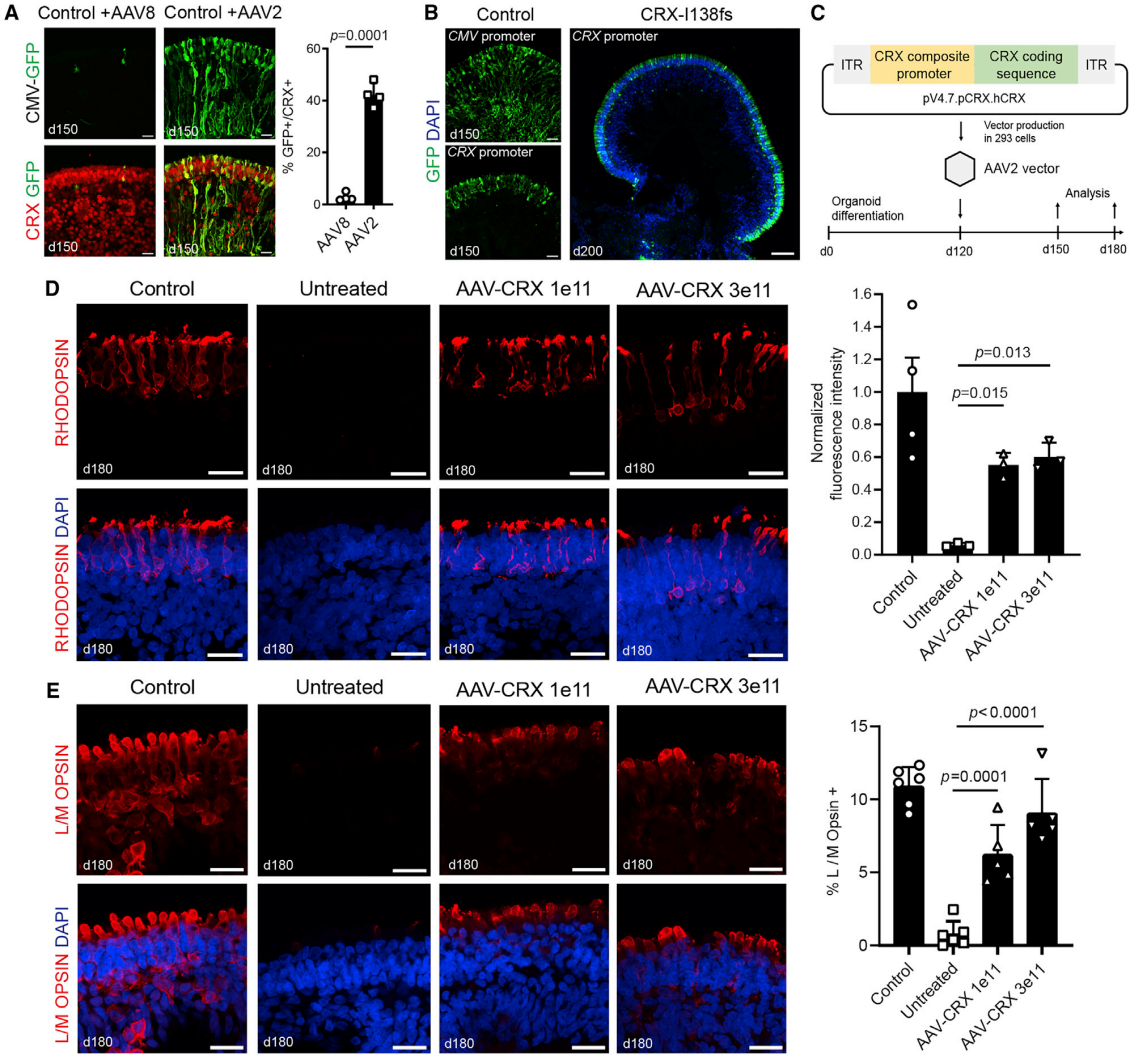
AAV-Delivered Gene Augmentation for *CRX*-LCA Caused by I138fs Mutation (A) Transduction of CRX-expressing cells by AAV2 and AAV8 serotypes. Quantification of percent of AAV-delivered GFP reporter in CRX-positive cells on control organoid cryosections. Scale bar, 20 μm. For both serotypes, n = 4 organoids, three sections each averaged; mean ± SD, statistical significance by Student’s t test, p value indicated. (B) Promoter testing in retinal organoids. Comparison of GFP reporter expression driven by CMV or CRX promoters in day 150 control organoids (left; scale bar, 20 μm). Note broad expression using CMV as compared with localization primarily to outer organoid layer with CRX promoter. GFP expression driven by CRX promoter in CRX-I138fs48 patient organoids at day 200 (right). Nuclei were stained with DAPI (4′,6-diamidino-2-phenylindole). Scale bar, 200 μm. (C) Schematic representation of the therapeutic AAV vector design and testing in retinal organoids. (D and E) Rhodopsin (D) and Cone L/M Opsin (E) staining in healthy control, and untreated and AAV-treated patient retinal organoids at day 180. Two doses of AAV-CRX vector were tested (1×10^11^ and 3×10^11^ vg per organoid). Scale bar, 20 μm. Efficiency of treatment was evaluated with quantification of Rhodopsin expression rescue and percentage of L/M Opsin + cones. Number of organoids analyzed for quantifications: (D) control n = 4, untreated and AAV-treated n = 3 each dose; (E) control and untreated n = 6; AAV-treated n = 5 each dose; three sections each. Values represent mean ± SD with individual data points plotted. Statistical significance was determined by one-way ANOVA; p values indicated.

**Figure 4:**
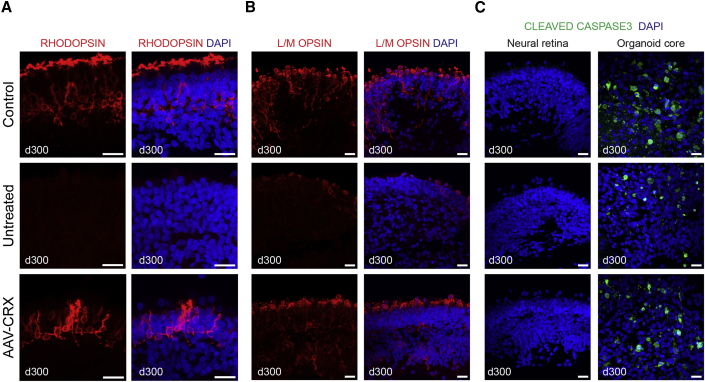
Long-Term Effects of AAV-CRX Gene Augmentation in CRX-I138fs Retinal Organoids Retinal organoids were transduced at day 120 with AAV-CRX vector at 1×10^11^ vg per organoid and examined 180 days later, at day 300 in culture. Immunostaining for (A) Rhodopsin, (B) L/M Opsin, (C) cleaved Caspase3. Nuclei were counterstained with DAPI. Note the continued presence of cells with rescued expression of opsins. Prolonged CRX expression does not appear to induce apoptotic cell death in the photoreceptor layer, where cleaved Caspase3 staining is absent (C, left), in contrast to the core region of the organoid showing extensive apoptosis in extended culture (C, right). Scale bar in all images, 20 μm.

Next, we asked whether gene augmentation with normal CRX might alleviate the disease phenotype by competing with the mutant isoform. First, we tested transduction with two commonly used AAV capsid serotypes AAV2 and AAV8 with cytomegalovirus (CMV) promoter driving a GFP reporter. AAV2 capsid showed much higher proportion of cells expressing both GFP and CRX in organoids at day 150, 10 days post vector addition at day 140 (Figure 3A). We then tested human *CRX* promoter sequences for driving transcription in retinal cells that normally express the *CRX* gene. Three promoter fragments were combined to generate a shorter composite promoter (see Table S5), which was placed upstream of GFP and packaged into AAV2 capsid (Figure 3B). As compared with transduction with ubiquitously expressed CMV promoter, *CRX* promoter-driven GFP signal localized to a distinct apical lamina corresponding to the photoreceptor layer in both control and CRX-I138fs patient organoids (Figure 3B). The final therapeutic construct contained this *CRX* composite promoter driving human *CRX* expression (Figure 3C). This AAV-*CRX* vector was added to patient organoids at day 120, and the samples were analyzed at days 150 and 180. Transduction with the vector increased CRX mRNA and protein in treated organoids (Figures S3A–S3D). Rhodopsin and L/M Opsin expression were used as surrogates for treatment, given their near absence in patient organoids. Retinal organoids transduced with 1×10^11^ vector genomes (vg) began to show rescue of Rhodopsin expression by day 150 (Figures S3E and S3F), which became more widespread by day 180 (Figure 3D). Quantification of Rhodopsin immunolabeling intensities confirmed higher fluorescence compared with untreated patient iPSC-derived organoids with both 1×10^11^ vg and 3×10^11^ vg doses and was almost half of signal intensity measured for the healthy control (Figure 3D). Fluorescence intensity levels were similar at both vector doses, suggesting that 1×10^11^ vg per organoid is sufficient for substantial rescue of Rhodopsin expression. Treatment also partially restored L/M Opsin expression (Figures 3E, S3G, and S3H). Rescue effect persisted at least for 6 months after treatment (day 300) with both Rhodopsin and L/M Opsin-positive cells still detectable (Figures 4A and 4B). Long-term expression of CRX did not lead to activation of the apoptotic marker Caspase3 in AAV-treated organoid neural retina (Figure 4C).

**Figure 5:**
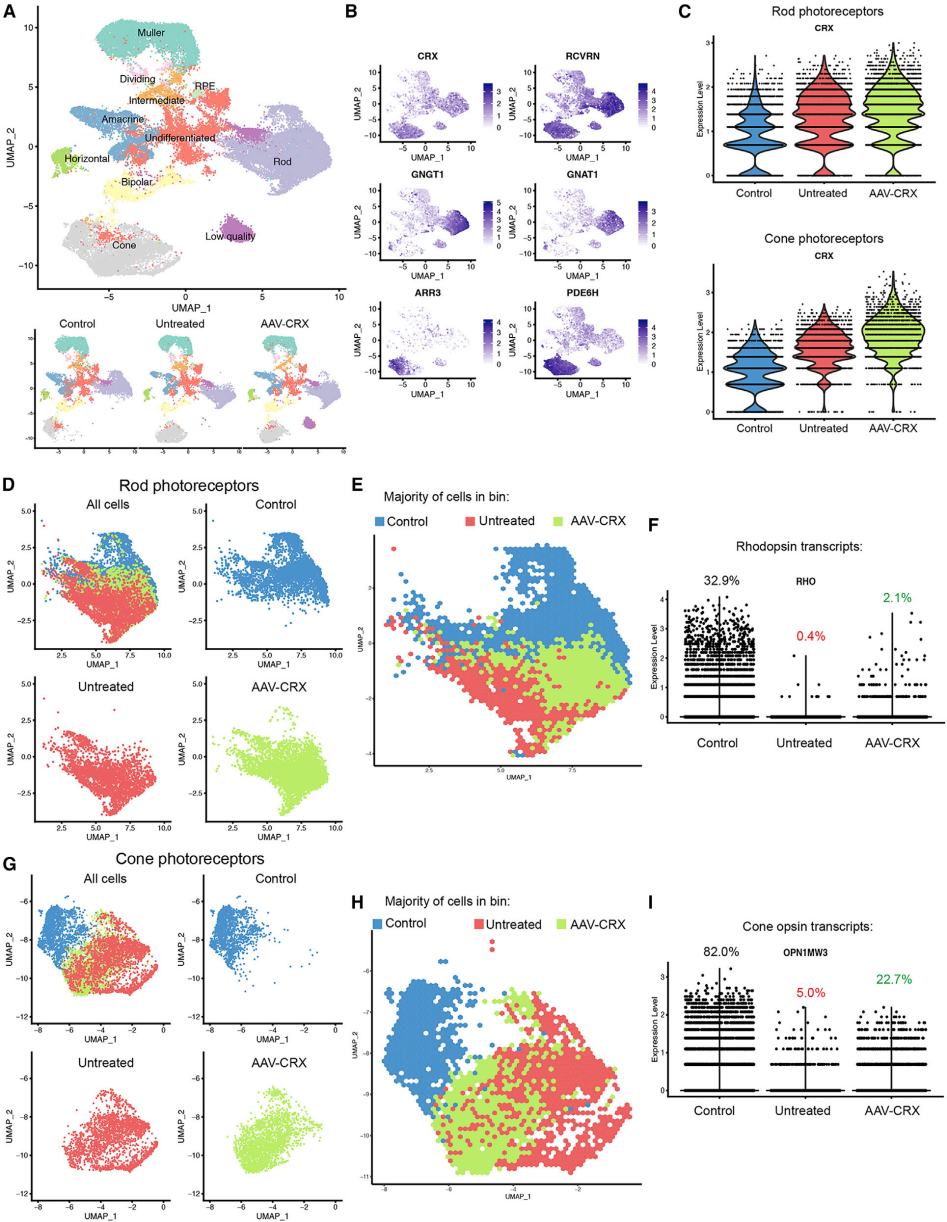
Altered Gene Expression Patterns and AAV Treatment Effects in Cone and Rod Photoreceptor Subtypes of CRX-I138fs48 Patient Retinal Organoids at day 200 (A) Top: UMAP representation of the single-cell RNA-seq dataset (n = 40,712 transcriptomes) displaying major cell types (annotated using known cell-type marker genes). Bottom: UMAP plots showing the distribution of cells of control (n = 2 biological replicates, 4 organoids each), and untreated and AAV-CRX (n = 2 biological replicates, 3 organoids each) organoid samples. (B) Expression of photoreceptor cell-type- (*CRX*, *RCVRN*) and subtype-specific markers (rods: *GNGT1*, *GNAT1*; cones: *ARR3*, *PDE6H*). (C) Violin plot profiles of CRX expression levels in rods and cones. Note increased expression with AAV-CRX gene augmentation. (D–I) Treatment effects in rod (D–F) and cone (G–I) photoreceptor subtypes. (D and G) UMAP plots showing the distribution of rod and cone cells by sample origin (control, blue; untreated, red; AAV-CRX, green). (E and H) Hexagonal bin plots illustrating identity of cell origin (coloring each hexagon according to the origin of the majority of cells it covers). Note placement of AAV-CRX treatment between patient and control sample majority areas. (F and I) Opsin transcript reads in the different samples visualizing increased expression in patient-derived samples following treatment. Percentages of cells of each origin in which transcript reads were detected are indicated.

To decipher and validate the impact of CRX-I138fs mutation on specific cell types within retinal organoids, we performed single-cell RNA-sequencing (scRNA-seq) using 10x Genomics platform. Control, untreated CRX-I138fs and AAV-treated organoids were dissociated using a papain-based method (Fadl et al., 2020) at day 200 yielding 40,712 single-cell transcriptional profiles. Data processing using Seurat package identified cell clusters, which were assigned to known retinal cell types and visualized using UMAP dimension reduction (Figure 5A and S4A–S4C). In this representation, major retinal cell classes (apart from ganglion cells) emerge from centrally located undifferentiated cells (Figure 5A). Cell-type distribution was similar across the three sample origins (Figures 5A and S4B). Rods and cones formed well-defined differentiation trajectories in this manifold and were identified by expression of both common (*CRX*, *RCVRN*) and subtype-specific markers (rod: *GNGT1*, *GNAT1*; cone: *ARR3*, *PDE6H;* Figures 5B, S4D,and S4E). As predicted, CRX transcripts increased in photoreceptors after AAV-CRX transduction (Figure 5C). Rod and cone expression profiles could be clearly separated based on control or patient sample origin, whereas AAV-treated cells occupied the space in between (Figures 5D, 5E, 5G, and 5H). This shift was particularly evident by plotting the origin of most cells across hexagonal bins (Figures 5E and 5H). ScRNA-seq detected partially rescued expression of opsins following AAV treatment (*RHO*, *OPN1MW3*, Figures 5F and 5I; *OPN1MW3* was the most significantly dysregulated of three medium wavelength opsin genes *OPN1MW1-3*), as well as of other rod- and cone-specific transcripts (Figures S4D and S4E; for each gene adjusted p value <0.05, nonparametric Wilcoxon rank-sum test with Bonferroni correction; minimum percent expressed = 10% cells, minimum log fold change = 0.25). CABP4, a retinal disease gene and direct transcriptional target of CRX (Assawachananont et al., 2018) showed a similar trend (Figures S4F and S4G). Thus, single-cell analysis confirmed treatment effect of AAV-mediated overexpression of normal CRX.

**Figure 6:**
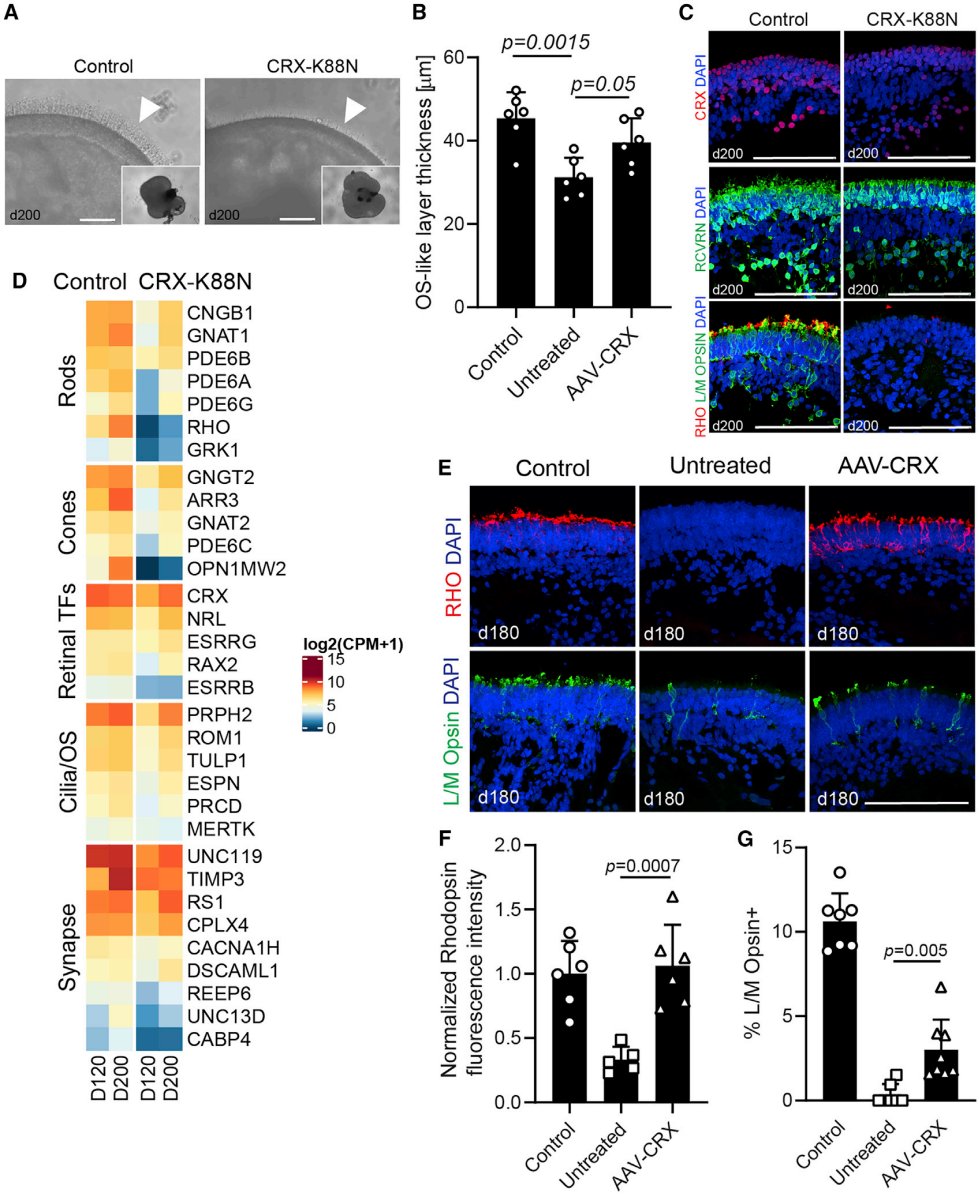
Disease Phenotype and Gene Augmentation Therapy of CRX-K88N Patient Retinal Organoids (A) Brightfield images of control and CRX-K88N patient organoids showing reduced outer segment (OS) apical “brush” layer (arrowheads). (B) Quantification of the outer segment-like layer thickness; n = 6 organoids per group, three sections each; mean ± SD, p values from one-way ANOVA. (C) Immunostaining of organoids at day 200 for CRX, Recoverin, Rhodopsin and L/M Opsin. Note diminished Rhodopsin and L/M Opsin staining in patient-derived organoids. Nuclei were stained with DAPI (4′,6-diamidino-2-phenylindole). Scale bar, 100 μm. (D) Heatmap comparing expression of genes (bulk RNA-seq) of day 120 and day 200 organoids. Expression of many photoreceptor-specific transcripts is either delayed or reduced in CRX-K88N patient samples. Normalized log2(CPM+1) values plotted. TFs, transcription factors; OS, outer segment. (E) AAV treatment assessment by immunostaining. Immunoreactivity for both Rhodopsin and L/M Opsin is partially restored following AAV treatment. Scale bar, 100 μm. (F) Quantification of Rhodopsin fluorescence intensity in AAV-treated retinal organoids. Control n = 6, untreated n = 5, and AAV-treated n = 6 organoids; three sections each; mean ± SD, p values from one-way ANOVA. (G) Quantification of the percentage of L/M Opsin + cones in AAV-treated retinal organoids. Control n = 7, untreated n = 7, and AAV-treated n = 8 organoids; three sections each; mean ± SD, p values from one-way ANOVA.

To determine whether the observed phenotypes and rescue were mutation-specific or could be generalized to other cases of dominant *CRX*-LCA, we examined organoids with the _CR_X _K8_8N mutation. As for the frameshift mutation, no differences in morphology and expression of key retinal markers were evident at stage 1 or 2 of organoid differentiation (Figures S5C–S5E). However, outer segment-like structures were less developed at stage 3 in patient iPSC-derived organoids compared with the control (Figures 6A and 6B). Immunostaining showed the presence of CRX and Recoverin, but severely diminished Rhodopsin and L/M Opsin staining in CRX-K88N organoids (Figure 6C). Transcriptome analyses at days 120 and 200 revealed delayed upregulation of many photoreceptor-specific genes (Figure 6D) and confirmed the loss of Rhodopsin and L/M Opsin expression (*RHO*, *OPN1MW2*; Figure 6D). Treatment of CRX-K88N organoids with AAV-CRX vector increased CRX levels (Figure S6A), partially rescued Rhodopsin and L/M Opsin expression (Figures 6E–6G) and reduced abnormal S Opsin levels (Figure S6B), with a modest induction of rod visual arrestin and synaptic proteins (Figure S6B). Thus, CRX-K88N organoids showed a similar phenotype to CRX-I138fs, and AAV gene therapy was able to restore expression of CRX target genes.

## Discussion

Dominant diseases represent a considerable challenge for developing gene therapy because of the presence of a mutant allele that interferes with normal function (Ahmed et al., 2019; Collin et al., 2019). Overexpression of a normal copy of Rhodopsin or VSX2 has been shown to partially correct defects in mouse or retinal organoid models of respective dominant disease (Mao et al., 2011; Phillips et al., 2014). We previously observed reexpression of Rhodopsin following electroporation of wild-type *Crx* into the retina of a mouse model harboring a spontaneous dominant frameshift mutation in the *Crx* gene (Roger et al., 2014). Building on that observation, we tested the hypothesis that overexpression of a correct *CRX* cDNA increasing the ratio of normal to mutant protein (gene augmentation) may rescue defects in dominant human *CRX* retinopathies. We developed retinal organoid models of dominant *CRX*-LCA from patients with two different mutation types and demonstrate the feasibility of using AAV-delivered *CRX* as a therapeutic strategy. We note that patient iPSC-derived organoids have recently been used to provide proof-of-concept for AAV-mediated gene therapy of recessive X-linked retinitis pigmentosa caused by mutations in *RP2* (Lane et al., 2020).

*CRX*-LCA patient organoids showed impaired maturation of photoreceptors with particularly disrupted expression patterns of visual opsins. Rhodopsin and L/M cone opsins were almost undetectable, whereas S Opsin expression was initially delayed but recovered later, consistent with findings in animal models (Roger et al., 2014; Tran et al., 2014). We observed increased total CRX protein levels in organoids of the patient with I138fs mutation, especially of the mutant isoform, whereas K88N organoids showed reduced overall levels of CRX protein. These findings suggest distinct potential downstream impacts of the dominant alleles: (1) competition of mutant protein with normal CRX for target binding sites, (2) prolonged occupancy of mutant protein at binding sites preventing the correct form from associating to the target DNA, and (3) disruption of stoichiometric interactions with other retinal transcription factors, such as NRL. The contribution of these mechanisms may vary depending on a specific mutation, but as we demonstrate, convergent molecular pathology such as disruption of opsin expression is evident.

We took advantage of scRNA-seq to determine disease-associated changes in photoreceptor subtypes as well as to evaluate treatment effect following gene therapy. We observed clear separation of patient cones and rods from control populations and reduced expression of cell-type-specific genes. In rods, partial shift toward control population with AAV treatment is associated with higher rescue of Rhodopsin expression in a subset of transduced cells, validating histological observations. Importantly, we detected continued rescue of Rhodopsin and L/M Opsin at 6 months posttreatment without any indication of apoptosis. Further investigations are needed to define optimal dosing as well as time window for gene therapy. In addition, we may require *in vivo* model(s) to fully evaluate the long-term impact of augmented CRX expression. In any event, single-cell transcriptomics offers a powerful approach to assess treatment approaches for retinopathies.

The difference in rescue of L/M Opsin expression was evident between patient organoids of the two genotypes, suggesting differential interaction of mutant CRX with binding partners in cones. While restoration of cone gene expression was clearly evident in our study, further enhancement is likely by selecting a more potent capsid serotype targeting cones (Gonzalez-Cordero et al., 2018). Treatment of cone photoreceptors is of relevance because mutations in *CRX* also cause milder adult-onset cone-rod dystrophies and macular dystrophies, which could potentially benefit from gene therapy treatment described here.

In conclusion, we used retinal organoids as a platform to examine disease mechanisms and design gene augmentation therapy for dominant *CRX*-LCA. We suggest that our experimental strategy should be applicable in developing effective treatments for rare, and even dominant, inherited diseases of the retina and other parts of the central nervous system.

## Experimental procedures

### Retinal organoids from patient-derived iPSCs

Skin biopsies were obtained from two CRX-LCA pediatric patients and respective healthy parental controls with informed consent (Table S1). Dermal fibroblasts were reprogrammed to generate iPSCs using a Sendai virus-based approach. Resulting iPSC lines were of normal karyotype and free of mycoplasma contamination (Table S2). iPSCs were differentiated following an established protocol (Kaya et al., 2019) with a minor modification of culturing dissected retinal organoids individually in a 96-well plate format (Table S3). Histology was performed as described in (Shimada et al., 2017), a list of all primary antibodies used for immunofluorescent staining is provided in Table S4. Images were obtained on Zeiss 700 confocal microscope.

### Next generation sequencing

RNA was isolated from three pooled frozen organoids per sample using the QIAGEN RNeasy Kit according to manufacturer's manual. RNA quality was assessed using the Agilent 2100 Bioanalyzer (Agilent Technologies). Samples (with RNA integrity number >7) were used for library generation using the TruSeq Library Preparation Kit (Illumina Inc). Sequencing and bioinformatic analysis were performed as described (Kaya et al., 2019).

For single-cell RNA-seq, organoids were dissociated following a papain-based method and subjected to single-cell isolation and sequencing as described (Fadl et al., 2020). Processing and analysis of single-cell transcriptomes using Seurat package is detailed in Supplemental information.

### Cloning of a composite human *CRX* promoter

Three fragments from the human CRX 5′ untranslated region (NCBI: NG_008605.1) were amplified from human genomic DNA and ligated to produce a 631 base pair promoter sequence (Table S5). In this promoter, 189 nucleotides correspond to positions 3,085–3,274, 69 correspond to 3,323–3,392, and 361 correspond to 4,808–5,169 of the reference. This promoter element contains first exon of the human *CRX* gene (nucleotides 4,999–5,169).

### Statistical analysis

At least three independently cultured organoids (individual wells of a 96-well plate) were used for quantification. Three differentiation batches were analyzed to verify disease phenotypes. In histological analyses, data from three sections were averaged to account for regional variation within individual organoids. With respect to next generation sequencing and single-cell sequencing, three organoids were pooled to obtain one sample. GraphPad Prism version 8.0 software was used to plot data and perform statistical analysis. For two group comparisons two-tailed Student’s t test was used. For multiple groups, one-way ANOVA analysis was used with either Dunnett's or Tukey's post hoc tests. Quantitative data are presented as means ± standard deviation (SD). Exact p values are reported in the figures, and p values of <0.05 were considered statistically significant.

### Study approval

Skin biopsies were obtained from the patients and unaffected family members at the National Eye Institute Clinical Center under an institutional review board-approved protocol #15-EI-0128, NCT01432847.

Detailed description of all experimental methods is provided in Supplemental information.

## Author contributions

Conceptualization, A. Swaroop, K.K.; Methodology, Investigation, Validation and Analysis, K.K., Z.Q., L.C., C.J.C., S.H., M.S., A.Samanta; Data curation and analysis, and Visualization, J.G., B.R.F., Z.B.; Resources, L.G., S.H., Z.W., B.P.B.; Writing – Original draft, K.K., A. Swaroop; Writing – Review & Editing, all authors; Project administration and supervision, Funding acquisition, A. Swaroop.

## Conflicts of interest

K.K., S.H., Z.W., and A.S. are inventors on a patent application “Gene therapy for treatment of CRX-associated retinopathies,” submitted by the National Eye Institute. Z.W. is now an employee of PTC Therapeutics. Other authors declare no conflict of interest.

## Accession numbers

The accession number for the RNA-seq data reported in this paper is GEO: GSE153101.

## Supplemental information

Document S1. Supplemental experimental procedures, Figures S1–S6, and Tables S1–S6

Document S2. Article plus Supplemental Information

## Footnotes

Supplemental information can be found online at https://doi.org/10.1016/j.stemcr.2020.12.018.

## References

[bib1] Ahmed C.M., Dwyer B.T., Romashko A., Van Adestine S., Park E.H., Lou Z., Welty D., Josiah S., Savinainen A., Zhang B. (2019). SRD005825 acts as a pharmacologic chaperone of opsin and promotes survival of photoreceptors in an animal model of autosomal dominant retinitis pigmentosa. Transl. Vis. Sci. Technol..

[bib2] Apte R.S. (2018). Gene therapy for retinal degeneration. Cell.

[bib3] Assawachananont J., Kim S.Y., Kaya K.D., Fariss R., Roger J.E., Swaroop A. (2018). Cone-rod homeobox CRX controls presynaptic active zone formation in photoreceptors of mammalian retina. Hum. Mol. Genet..

[bib4] Capowski E.E., Samimi K., Mayerl S.J., Phillips M.J., Pinilla I., Howden S.E., Saha J., Jansen A.D., Edwards K.L., Jager L.D. (2019). Reproducibility and staging of 3D human retinal organoids across multiple pluripotent stem cell lines. Development.

[bib5] Collin J., Queen R., Zerti D., Dorgau B., Hussain R., Coxhead J., Cockell S., Lako M. (2019). Deconstructing retinal organoids: single cell RNA-seq reveals the cellular components of human pluripotent stem cell-derived retina. Stem Cells.

[bib6] Cowan C.S., Renner M., De Gennaro M., Gross-Scherf B., Goldblum D., Hou Y., Munz M., Rodrigues T.M., Krol J., Szikra T. (2020). Cell types of the human retina and its organoids at single-cell resolution. Cell.

[bib7] den Hollander A.I., Roepman R., Koenekoop R.K., Cremers F.P. (2008). Leber congenital amaurosis: genes, proteins and disease mechanisms. Prog. Retin. Eye Res..

[bib8] Fadl B.R., Brodie S.A., Malasky M., Boland J.F., Kelly M.C., Kelley M.W., Boger E., Fariss R., Swaroop A., Campello L. (2020). An optimized protocol for retina single-cell RNA sequencing. Mol. Vis..

[bib9] Furukawa T., Morrow E.M., Li T., Davis F.C., Cepko C.L. (1999). Retinopathy and attenuated circadian entrainment in Crx-deficient mice. Nat. Genet..

[bib10] Gonzalez-Cordero A., Goh D., Kruczek K., Naeem A., Fernando M., Kleine Holthaus S.M., Takaaki M., Blackford S.J.I., Kloc M., Agundez L. (2018). Assessment of AAV vector tropisms for mouse and human pluripotent stem cell-derived rpe and photoreceptor cells. Hum. Gene Ther..

[bib11] Hennig A.K., Peng G.H., Chen S. (2008). Regulation of photoreceptor gene expression by Crx-associated transcription factor network. Brain Res..

[bib12] Hoshino A., Ratnapriya R., Brooks M.J., Chaitankar V., Wilken M.S., Zhang C., Starostik M.R., Gieser L., La Torre A., Nishio M. (2017). Molecular anatomy of the developing human retina. Dev. Cell.

[bib13] Huang L., Xiao X., Li S., Jia X., Wang P., Guo X., Zhang Q. (2012). CRX variants in cone-rod dystrophy and mutation overview. Biochem. Biophys. Res. Commun..

[bib14] Hull S., Arno G., Plagnol V., Chamney S., Russell-Eggitt I., Thompson D., Ramsden S.C., Black G.C., Robson A., Holder G.E. (2014). The phenotypic variability of retinal dystrophies associated with mutations in CRX, with report of a novel macular dystrophy phenotype. Invest. Ophthalmol. Vis. Sci..

[bib15] Ibrahim M.T., Alarcon-Martinez T., Lopez I., Fajardo N., Chiang J., Koenekoop R.K. (2018). A complete, homozygous CRX deletion causing nullizygosity is a new genetic mechanism for Leber congenital amaurosis. Sci. Rep..

[bib16] Kaya K.D., Chen H.Y., Brooks M.J., Kelley R.A., Shimada H., Nagashima K., de Val N., Drinnan C.T., Gieser L., Kruczek K. (2019). Transcriptome-based molecular staging of human stem cell-derived retinal organoids uncovers accelerated photoreceptor differentiation by 9-cis retinal. Mol. Vis..

[bib17] Kim S., Lowe A., Dharmat R., Lee S., Owen L.A., Wang J., Shakoor A., Li Y., Morgan D.J., Hejazi A.A. (2019). Generation, transcriptome profiling, and functional validation of cone-rich human retinal organoids. Proc. Natl. Acad. Sci. U S A.

[bib18] Kruczek K., Swaroop A. (2020). Pluripotent stem cell-derived retinal organoids for disease modeling and development of therapies. Stem Cells.

[bib19] Kumaran N., Moore A.T., Weleber R.G., Michaelides M. (2017). Leber congenital amaurosis/early-onset severe retinal dystrophy: clinical features, molecular genetics and therapeutic interventions. Br. J. Ophthalmol..

[bib20] Lane A., Jovanovic K., Shortall C., Ottaviani D., Panes A.B., Schwarz N., Guarascio R., Hayes M.J., Palfi A., Chadderton N. (2020). Modeling and rescue of RP2 retinitis pigmentosa using iPSC-derived retinal organoids. Stem Cell Rep..

[bib21] Mao H., James T., Schwein A., Shabashvili A.E., Hauswirth W.W., Gorbatyuk M.S., Lewin A.S. (2011). AAV delivery of wild-type rhodopsin preserves retinal function in a mouse model of autosomal dominant retinitis pigmentosa. Hum. Gene Ther..

[bib22] Mitton K.P., Swain P.K., Chen S., Xu S., Zack D.J., Swaroop A. (2000). The leucine zipper of NRL interacts with the CRX homeodomain. A possible mechanism of transcriptional synergy in rhodopsin regulation. J. Biol. Chem..

[bib23] Nichols L.L., Alur R.P., Boobalan E., Sergeev Y.V., Caruso R.C., Stone E.M., Swaroop A., Johnson M.A., Brooks B.P. (2010). Two novel CRX mutant proteins causing autosomal dominant Leber congenital amaurosis interact differently with NRL. Hum. Mutat..

[bib24] Phillips M.J., Perez E.T., Martin J.M., Reshel S.T., Wallace K.A., Capowski E.E., Singh R., Wright L.S., Clark E.M., Barney P.M. (2014). Modeling human retinal development with patient-specific induced pluripotent stem cells reveals multiple roles for visual system homeobox 2. Stem Cells.

[bib25] Rivolta C., Berson E.L., Dryja T.P. (2001). Dominant Leber congenital amaurosis, cone-rod degeneration, and retinitis pigmentosa caused by mutant versions of the transcription factor CRX. Hum. Mutat..

[bib26] Roger J.E., Hiriyanna A., Gotoh N., Hao H., Cheng D.F., Ratnapriya R., Kautzmann M.A., Chang B., Swaroop A. (2014). OTX2 loss causes rod differentiation defect in CRX-associated congenital blindness. J. Clin. Invest..

[bib27] Shimada H., Lu Q., Insinna-Kettenhofen C., Nagashima K., English M.A., Semler E.M., Mahgerefteh J., Cideciyan A.V., Li T., Brooks B.P. (2017). In vitro modeling using ciliopathy-patient-derived cells reveals distinct cilia dysfunctions caused by CEP290 mutations. Cell Rep..

[bib28] Swaroop A., Wang Q.L., Wu W., Cook J., Coats C., Xu S., Chen S., Zack D.J., Sieving P.A. (1999). Leber congenital amaurosis caused by a homozygous mutation (R90W) in the homeodomain of the retinal transcription factor CRX: direct evidence for the involvement of CRX in the development of photoreceptor function. Hum. Mol. Genet..

[bib29] Tran N.M., Zhang A., Zhang X., Huecker J.B., Hennig A.K., Chen S. (2014). Mechanistically distinct mouse models for CRX-associated retinopathy. PLoS Genet..

[bib30] Veleri S., Lazar C.H., Chang B., Sieving P.A., Banin E., Swaroop A. (2015). Biology and therapy of inherited retinal degenerative disease: insights from mouse models. Dis. Model. Mech..

[bib31] Watanabe S., Sanuki R., Ueno S., Koyasu T., Hasegawa T., Furukawa T. (2013). Tropisms of AAV for subretinal delivery to the neonatal mouse retina and its application for in vivo rescue of developmental photoreceptor disorders. PLoS One.

[bib32] Zhong X., Gutierrez C., Xue T., Hampton C., Vergara M.N., Cao L.H., Peters A., Park T.S., Zambidis E.T., Meyer J.S. (2014). Generation of three-dimensional retinal tissue with functional photoreceptors from human iPSCs. Nat. Commun..

