# Supplementary material for: Gene Therapy of Dominant CRX-Leber Congenital Amaurosis using Patient Stem Cell-Derived Retinal Organoids
Source: Stem Cell Reports. 2021 Jan 28;16(2):252–63. doi: 10.1016/j.stemcr.2020.12.018 (PMC7878833; doi:10.1016/j.stemcr.2020.12.018)
Supplement: Document S1. Supplemental experimental procedures, Figures S1–S6, and Tables S1–S6 [file mmc1.pdf]

Stem Cell Reports, Volume *16*

Supplemental Information

Gene Therapy of Dominant *CRX*-Leber Congenital Amaurosis using Patient Stem Cell-Derived Retinal Organoids

Kamil Kruczek, Zepeng Qu, James Gentry, Benjamin R. Fadl, Linn Gieser, Suja Hiriyanna, Zachary Batz, Mugdha Samant, Ananya Samanta, Colin J. Chu, Laura Campello, Brian P. Brooks, Zhijian Wu, and Anand Swaroop

Supplemental Information

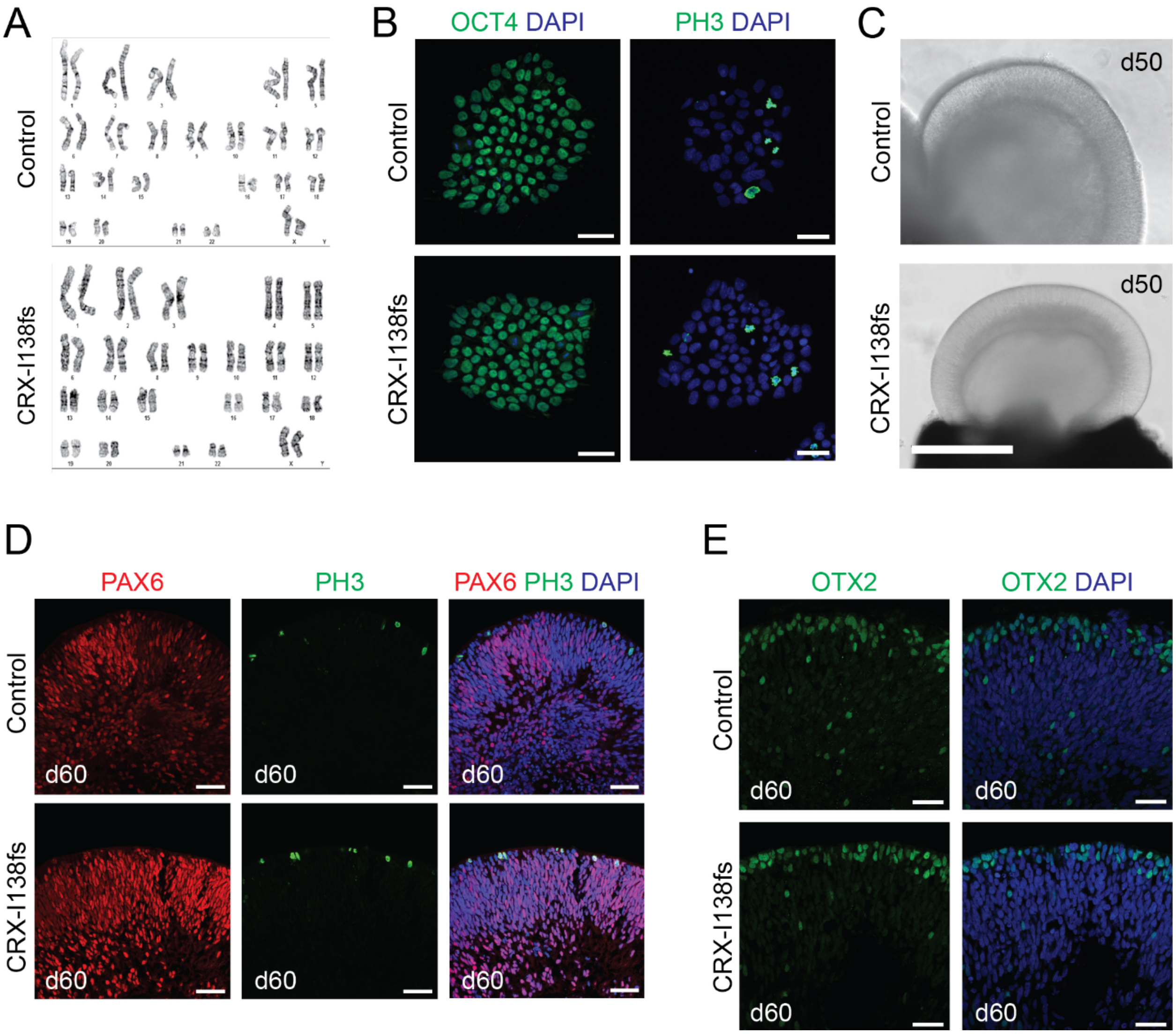

Figure S1. Differentiation of retinal organoids from control and CRX-I138fs iPSCs. Related to Figure 1.

(**A**) Representative karyograms for control and CRX-I138fs iPSC lines. (**B**) Staining of iPSCs for pluripotency (OCT4) and proliferation markers (phospho-histone 3, PH3). Scale bar, 10 µm. (**C**) Brightfield images of optic vesicle structures in control and patient organoids at differentiation day 50. Scale bar, 400 µm. (**D**) Immunostaining of organoids for early retinal marker PAX6 and proliferation marker PH3. Scale bar, 20 µm. (**E**) Immunostaining for retinal progenitor and early photoreceptor marker OTX2. Scale bar, 20 µm. Note indistinguishable patterns between control and patient organoids. Nuclei were stained with DAPI (4′,6-diamidino-2-phenylindole).

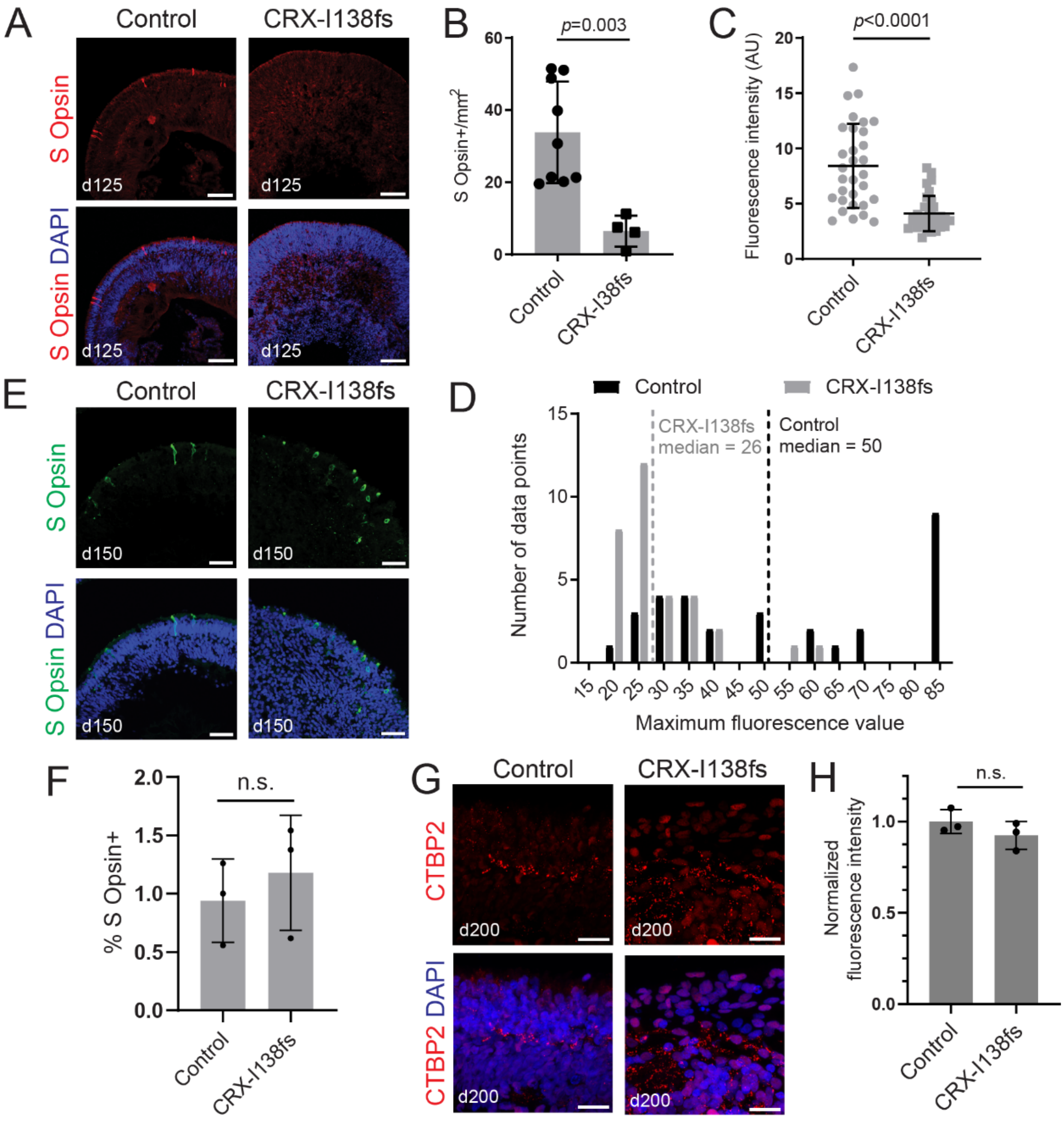

Figure S2. CRX-I138fs impairs specific aspects of photoreceptor differentiation. Related to Figure 2.

Delayed S opsin expression in CRX-I138fs organoids. (**A**) S opsin immunostaining in organoids at d125. Scale bar, 50 µm. S Opsin+ cells are mostly undetectable in the patient organoids. (**B**) Quantification of S Opsin+ cells per organoid section area at d125; control n=9, patient n=4 organoids; mean±SD. (**C**) Fluorescence intensity measurement (AU – arbitrary units) for S Opsin+ cells at d125; control n=31, patient n=32 cells; mean±SD. (**D**) Histogram of maximum fluorescence intensity values in S Opsin+ cells at d125. Control n=31, patient n=32 cells. Note the shift to lower values in patient samples. (**E**) Immunostaining for S Opsin at d150 shows induction of its expression in CRX-I138fs patient organoids by this timepoint. (**F**) Quantification of staining in (E) at d150, n=3 each group. (**G**) Immunostaining for CTBP2 (Ribeye) at d200. CTBP2-staining synaptic puncta are present in both genotypes. (**H**) Quantification of CTBP2 fluorescence intensity at d200, 3 organoids of each genotype were assessed, mean±SD plotted. Statistical significance was determined using Student's *t*-test for all quantifications; *p* values are indicated. In A, E, G: Nuclei were stained with DAPI (4′,6-diamidino-2-phenylindole).

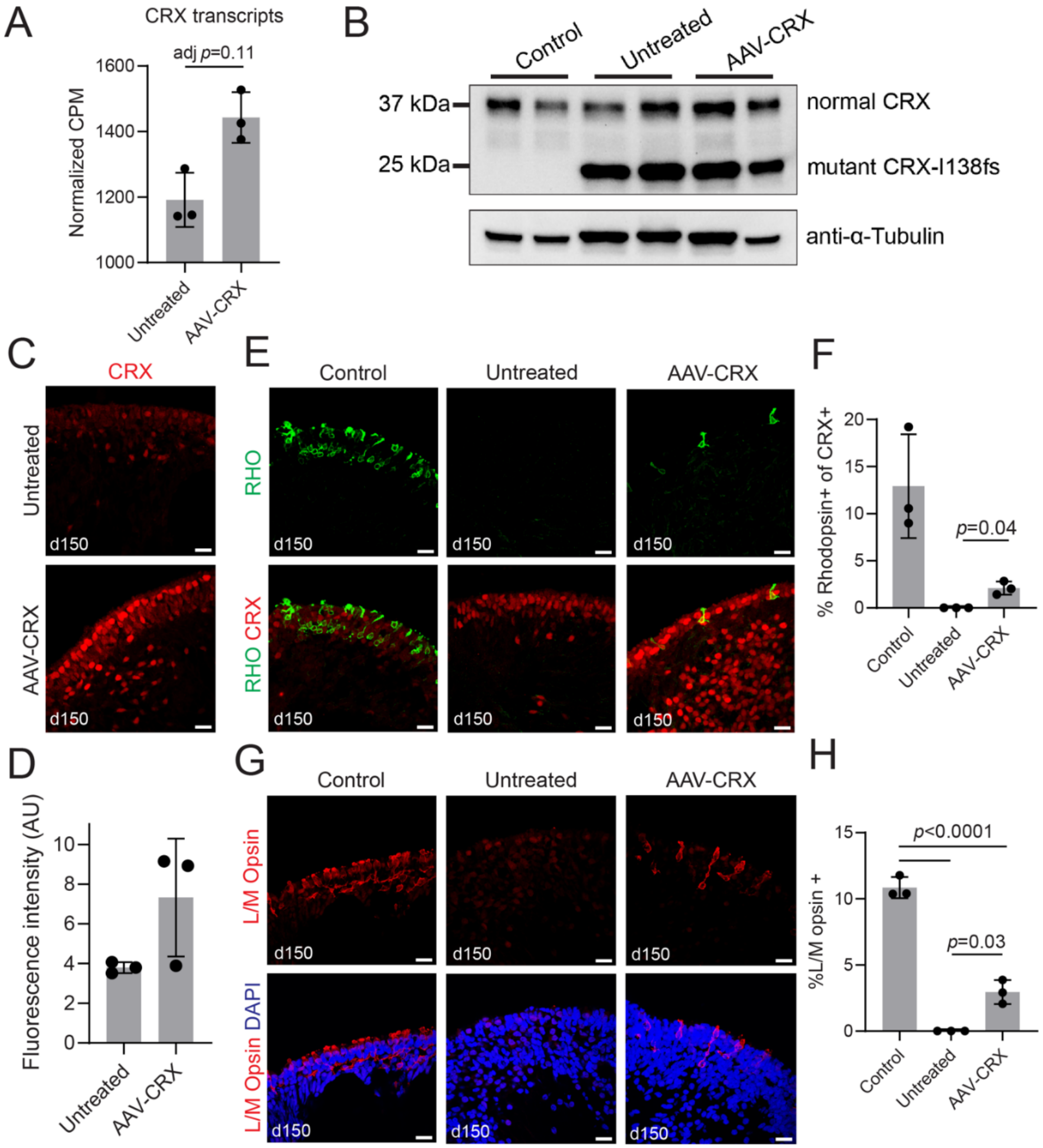

Figure S3. CRX gene augmentation in CRX-I138fs patient organoids. Related to Figure 3.

(**A**) Normalized CRX transcript counts in bulk RNA-seq data of untreated and AAV-transduced organoids at d150. (**B**) Immunoblot analysis of d200 control, untreated and AAV-treated protein samples using anti-CRX antibody. α-Tubulin was used as loading control. Molecular mass of the two CRX isoforms is indicated on the left. (**C**) Immunostaining of CRX in untreated and AAV-CRX treated organoids. Scale bar, 20 µm. (**D**) Quantification of CRX immunolabeling intensity with 3 organoids per group. (**E**) Immunostaining of Rhodopsin and CRX in control, untreated and AAV-CRX treated CRX-I138fs retinal organoids at d150, 30 days following the vector addition (at d120). (**F**) Quantification of immunostaining in (E); n=3 organoids for each group, data presented as percent of CRX+ cells. (**G**) Immunostaining of L/M Opsin of samples as in (E). Nuclei were stained with DAPI (4′,6-diamidino-2-phenylindole). (**H**) Quantification of immunostaining shown in (G), n=3 organoids per group, data presented as percent of all nuclei. Values in D,F,H represent mean ± SD with individual data points plotted. Statistical significance was determined by one-way ANOVA with Tukey's post hoc test for multiple comparisons. Significant *p* values are indicated.

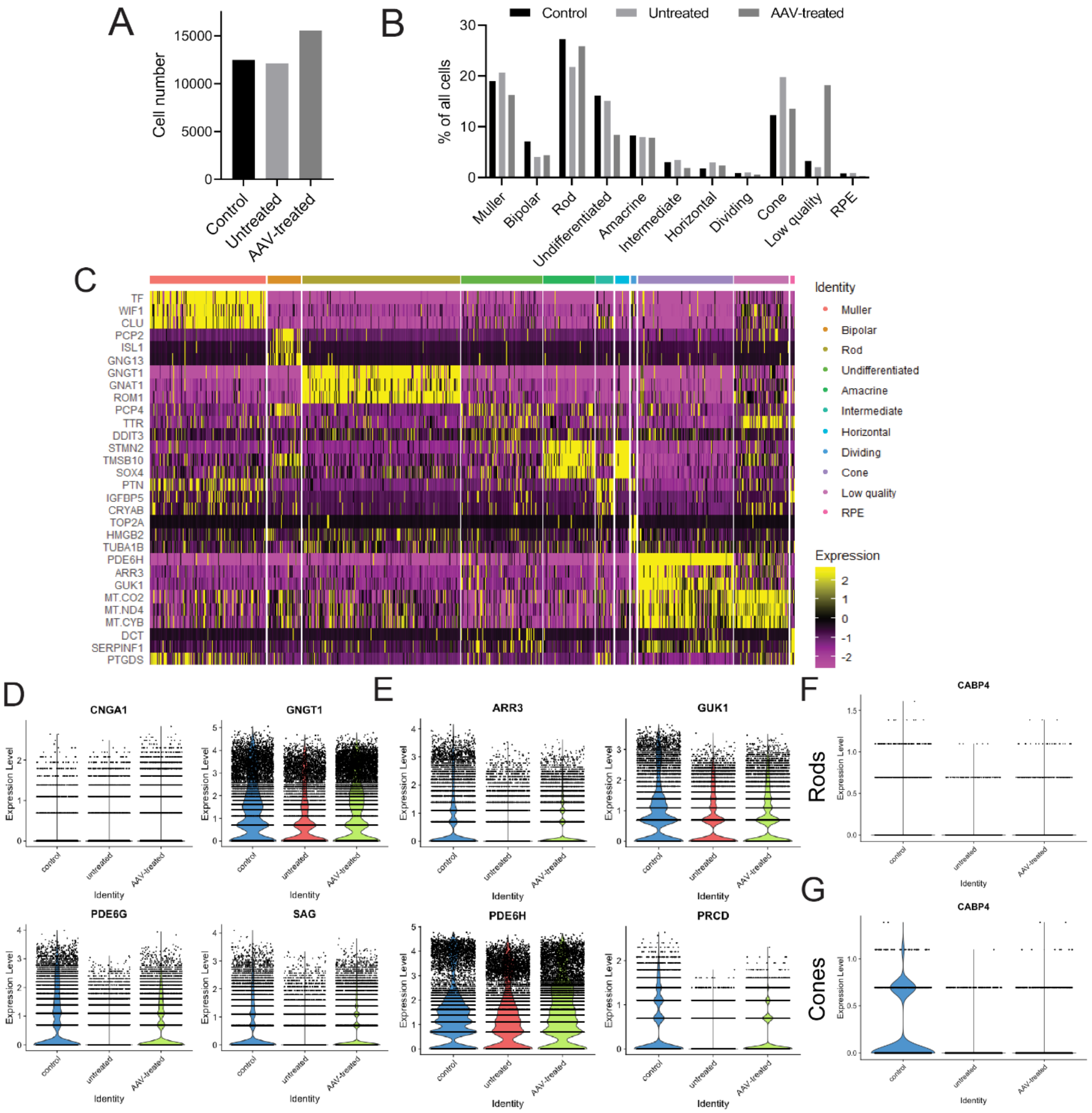

Figure S4. Cell type diversity in *CRX*-LCA retinal organoids and gene augmentation effects revealed by single cell transcriptomics. Related to Figure 5.

(**A**) Number of single cell transcriptome profiles obtained from control (12496 cells), untreated (12126 cells) and AAV-treated CRX-I138fs (15550 cells) organoids at d200. (**B**) Cell type distributions across various conditions. (**C**) Heatmap of 3 top transcripts most significantly enriched in each assigned cell class in the combined data set. The molecular markers were used to define cell types across experimental conditions. (**D**) Examples of rod-specific transcript expression rescued by AAV treatment in rod photoreceptors. For all genes adjusted p value < 0.05, Wilcoxon rank sum test with Bonferroni correction; min. percent expressed = 10% cells, min. log fold change = 0.25. (**E**) Example of cone transcripts rescued by AAV treatment. (**F,G**) Expression of CABP4, a retinal disease-associated direct transcriptional target of CRX, in rod (F) and cone (G) photoreceptors, showing a trend toward higher expression after AAV treatment.

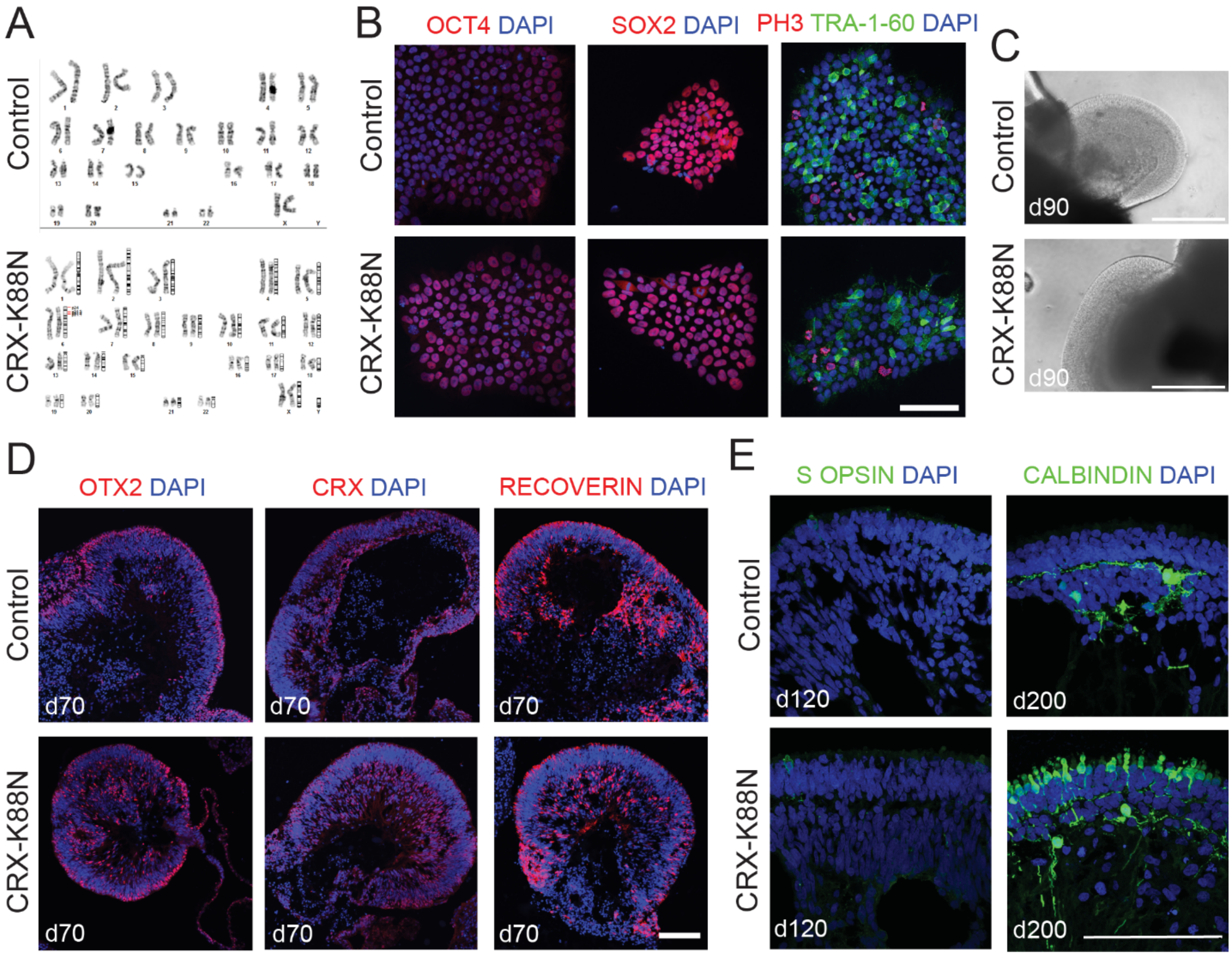

Figure S5. Differentiation of retinal organoids from CRX-K88N patient iPSCs. Related to Figure 6.

(**A**) Representative karyograms of control and CRX-K88N iPSC lines. (**B**) Immunostaining of control and CRX-K88N iPSC colonies using pluripotency (OCT4, SOX2, TRA-1-60) and proliferation (PH3) markers. (**C**) Brightfield images of organoid retinal epithelia at d90. Scale bar, 200 µm. (**D**) Immunostaining for OTX2, CRX and Recoverin in control and CRX-K88N retinal organoids at d70 of differentiation. All three markers showed similar staining between the two genotypes. (**E**) Immunostaining for S Opsin and Calbindin in control and CRX-K88N retinal organoids. Note an increased Calbindin staining in patient organoids at d200. Nuclei were stained with DAPI (4′,6-diamidino-2-phenylindole). Scale bars in B,D,E, 100 µm.

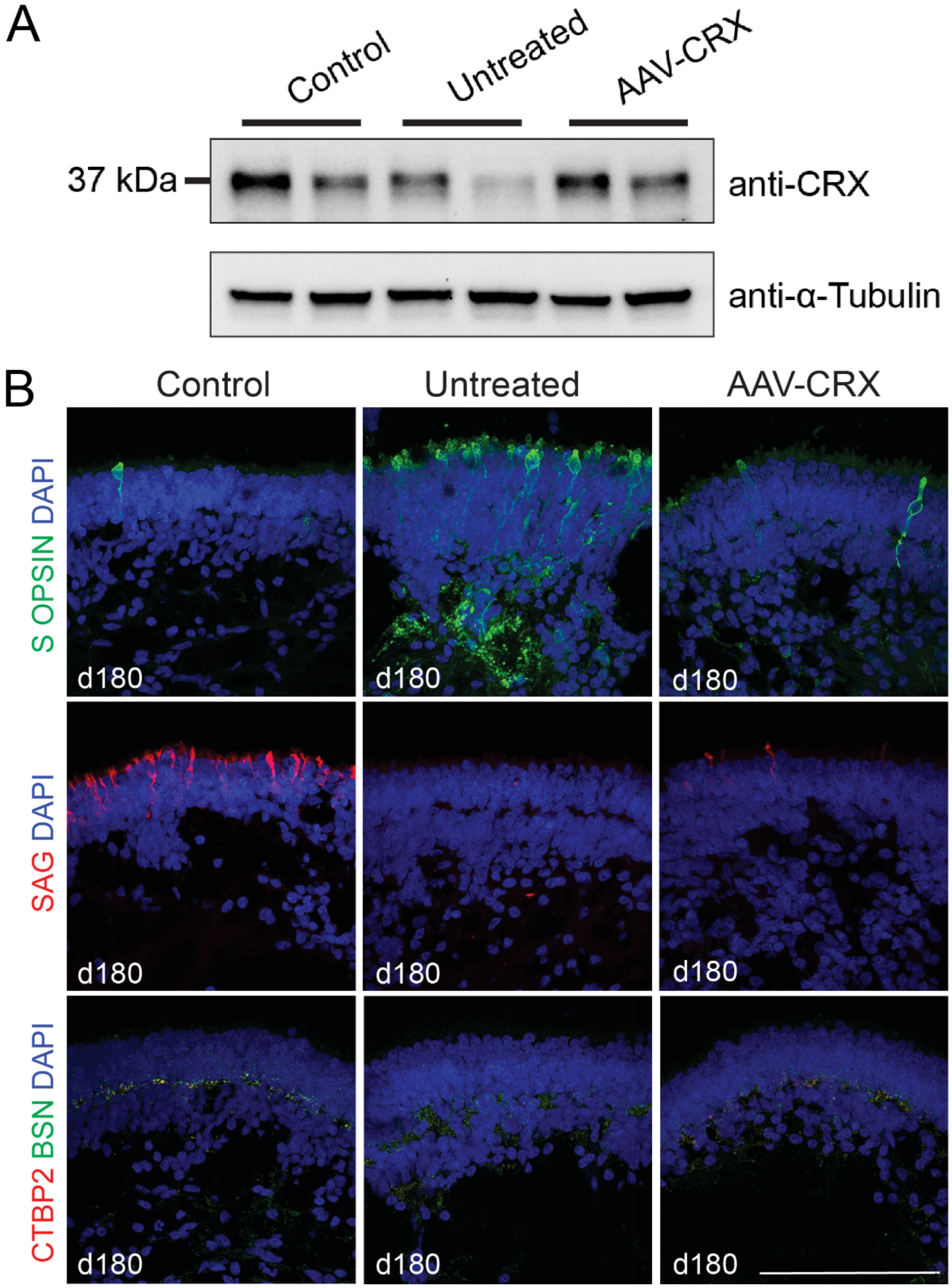

Figure S6. AAV treatment of CRX-K88N retinal organoids. Related to Figure 6.

(**A**) Immunoblot analysis of CRX protein in control, untreated and AAV-treated organoids at d200. α-tubulin was used as a loading control. Molecular mass of CRX is indicated on the left. (**B**) Immunostaining of retinal markers – S Opsin, SAG (rod Visual Arrestin) and synaptic proteins CTBP2 (Ribeye) and Bassoon (BSN) – in control, untreated and AAV-treated CRX-K88N organoids. Abnormally high S Opsin staining is reduced following AAV treatment; n=4 organoids examined in each group showed a consistent pattern. SAG expression which is undetectable in the patient organoids is modestly rescued and synaptic areas show qualitatively more staining after CRX augmentation. Nuclei were stained with DAPI. Scale bar, 100 μm.

Table S1. Study subjects. Related to all figures.

Patient ID	Disease status	Family relationship	Genotype	Protein variant	Sex	Age
NEI 001 Control	Healthy	Mother	Normal	Normal	F	36
NEI 001 Patient	*CRX*-LCA	Daughter	c.G264T	CRX-K88N	F	6
NEI 002 Control	Healthy	Mother	Normal	Normal	F	38
NEI 002 Patient	*CRX*-LCA	Daughter	c.413delT	CRX-I138fs	F	5

Table S2. IPSC line derivation and characterization. Related to all figures.

IPSC line	Derived from	Karyotype	Pluripotency markers	Mycoplasma status
NEI 001 Control B	NEI 001 Control	Normal, 46 XX	OCT4, SOX2, TRA-1-60	Negative
NEI 001 Patient A	NEI 001 Patient	Normal, 46 XX	OCT4, SOX2, TRA-1-60	Negative
NEI 002 Control A	NEI 002 Control	Normal, 46 XX	OCT4, SOX2, TRA-1-60	Negative
NEI 002 Patient B	NEI 002 Patient	Normal, 46 XX	OCT4, SOX2, TRA-1-60	Negative

Table S3. Retinal differentiation protocol. Related to all figures.

Day	Procedure
**0**	Detach the iPSCs by EDTA solution, dissociate into small clumps by pipetting a few times. Culture in suspension in E8 medium with ROCK inhibitor (final concentration: 10 µM) in ultra-low attachment dishes (9 ml in 100 mm diameter culture dish).
**1**	Add 3 ml of 1:1 NIM (composed of DMEM/F12 with Glutamax, 1% N2 supplement, 1x Minimum essential media-non essential amino acids, 2µg/ml Heparin) – ratio E8 to NIM 3:1
**2**	Add 6 ml of 1:1 NIM – ratio E8 to NIM 1:1
**3**	Collect the embryoid bodies (EBs) to a 14 ml tube and aspirate the supernatant. Suspend EBs in full 1:1 NIM (around 10 ml per dish).
**6**	Prepare Matrigel-coated dishes for day 7.
**7**	Seed the aggregates (average size of 0.22 ± 0.05 mm) onto Matrigel-coated dishes containing 1:1 NIM at an approximate density of 20 aggregates per cm^2.
**7-16**	Change the medium every 2-3 days.
**16**	Switch the medium to 3:1 NIM (composed of 3 parts DMEM, 1 part F12, 2% B27 without vitamin A, 2mM Glutamax, 1x Minimum essential media-non essential amino acids, 1% Antibiotic-antimycotic). Change medium every 2-3 days.
**16-35**	Change the medium every 2-3 days.
**28-35**	Detach horseshoe-shaped prospective neural retina domains manually with a sharpened Tungsten needle under inverted microscope. Collect retinal domains and culture in 3:1 NIM with 20 ng/ml IGF-1 in an ultra-low attachment U bottom 96-well plate.
**35-42**	Change the medium every 2-3 days.
**42**	Supplement the medium with 10% fetal bovine serum (Gibco), 100µM Taurine (Sigma) and IGF-1.
**42-63**	Change the medium every 2-3 days.
**63-91**	Use 3:1 NIM with 10% FBS, 20 ng/ml IGF-1, 100 µM taurine (Tau) and 1 µM 9-cis-retinaldehyde. Change every 2-3 days.
**91 onwards**	Lower the concentration of 9-cis-retinaldehyde to 0.5 µM. Replace every 2-3 days.

Table S4. List of primary antibodies used in the study. Related to all figures.

Antigen	Species/type	Dilution	Source	Identifier
Bassoon	Rabbit monoclonal	1:200	Cell Signaling	6897
Calbindin	Rabbit polyclonal	1:400	Millipore/Calbiochem	PC253L
CRX	Mouse monoclonal	1:100	Abnova	H00001406-M02
CTBP2	Mouse monoclonal	1:200	BD Transduction Laboratories	612044
GFP	Goat polyclonal	1:200	Rockland	600-101-215
L/M Opsin	Rabbit polyclonal	1:250	Millipore	AB5405
OCT4	Rabbit polyclonal	1:500	Abcam	ab19857
OTX1/2	Rabbit polyclonal	1:200	Abcam	ab21990
PAX6	Mouse monoclonal	1:200	DSHB	AB_528427
Peripherin2	Chicken polyclonal	1:200	Tiansen Li	n.a.
Pospho-Histone H3	Rabbit polyclonal	1:250	Cell Signaling	#9701
Recoverin	Rabbit polyclonal	1:500	Chemicon International	AB5585
Rhodopsin	Mouse monoclonal	1:500	Robert Molday	Clone 1D4
SAG	Mouse monoclonal	1:500	Abcam	ab190315
SOX2	Rabbit polyclonal	1:100	STEMGENT	09-0024
S Opsin	Rabbit polyclonal	1:250	Millipore	AB5407
TRA-1-60	Mouse monoclonal	1:200	Millipore	MAB4360
α-Tubulin	Mouse monoclonal	1:2500	Abcam	Ab7291
VSX2	Sheep polyclonal	1:200	Abcam	ab16142

Table S5. Sequence of composite human CRX promoter. Related to Figure 2.

**Composite human CRX promoter, 631 nucleotides**
CGTCGACGGGTCAGACGGCCCCTCCCTCTCTTGCTGTCATCCCTGGCTCTTCAAGCTAATGAGACCT<br>GTCCTGATTCCTCAGCCAGGCCTGTAGCCTTAATCTCTCCTAGCAGGGGGTTTGGGGGAGGGAGGA<br>GGAGAAAGAAAGGGCCCCTTATGGCTGAGACACAATGACCCAGCCACAAGGAGGGATTACCGGGG<br>AAGTGAAACAGACCCGTGTGGGACCCAGGAGCTCAGGGACATATTAATATCTAGAGAGACAGACG<br>GTCGACAGACACCAGTTAGACCTAAGGAAGGACTTCCCTGAGGAGTAGGGGCTTATGGTCACCGGC<br>AGGAGCTGGGGCCTCCCTTCCCCATCAGCCCTAATTGCCAAGATGTCATGGGGGGAAGAGGAGGGG<br>ATTAAGCAGACGGGTGCCCCTCCCCCTCCCAGCCAATGTCACCTCCTGGTGCCCAGTCGAGTCCCCC<br>ACCTTGGCCGGGATTACCCTCCGAGTTCCAGGCCATAACAAGTGACATCACTCCCGGCCCAGGCTTA<br>AAATCTCCCCACGTGAGGGGACGTGTTTCCTTCAGCCTCTGCTGTCTGGCCGCTCTGTCTAGGTCCTG<br>GGCCACGGGAGAGCCCCGTCCCTCCTTTCTGAAG

Table S6. AAV vector production protocol. Related to Figure 2.

Step	Procedures
AAV production by transient transfection	Seed HEK 293 cells in 5 roller bottles at a density of 3×10^7 cells per bottle in 300 ml DMEM medium with 10% FBS. Culture in a tissue-culture incubator at 37ºC in 5% CO_2.
	When cells reach 80% confluency add 5 ml of 1 M HEPES buffer for pH stabilization. Prepare transfection solution by mixing 150 μg of vector transgene plasmid, 150 μg of pHLP19-AAV2 capsid plasmid, 150 μg of pLAdeno5 helper plasmid with 15 ml 0.3 M $CaCl_2$ and 15 ml of 2×HBSS buffer. Mix gently by pipetting. Add the transfection solution to roller bottle immediately.
	Incubate with transfection mix 6 h to overnight at 37ºC in 5% CO_2.
	Replace medium with 100 ml of DMEM serum-free medium.
	48 h following transfection detach cells by vigorous swirling and harvest.
	Pool cells from all 5 roller bottles into one 500 ml conical tube.
	Centrifuge at 3000×*g* for 30 min. at 4ºC.
	Discard supernatant and resuspend cell pellet in 200 ml of TSM buffer. Cell pellet can either be used immediately for purification or stored at -80ºC. If stored, frozen pellet should be thawed in a water bath at 37ºC before proceeding to purification.
Isolation and purification of AAV	Homogenize cell pellet using a microfluidizer.
	In order to remove cell debris centrifuge at 3000×*g* for 30 min. and transfer supernatant into a fresh 500 ml centrifuge tube.
	Add 1M $CaCl_2$ to a final concentration of 25 mM. Mix well and leave to incubate for 10 min. at 4ºC.
	Centrifuge at 3000×*g* for 1 h. Collect supernatant and transfer into a fresh 500 ml centrifuge tube.
	Digest residual free DNA by treatment with Benzonase at 100 U/ml for 1 h at 37ºC.
	Precipitate AAV particles by adding 40% PEG8000 / 2.5 N NaCl to a final concentration of 8% PEG. Thoroughly mix, incubate for 2 h on ice.
	Centrifuge at 3000×*g* for 30 min. at 4ºC. Discard the supernatant.
	Resuspend the pellet in 25 ml of HSSE-RNase A buffer. Incubate for 30 min. at 37ºC.
	Prepare CsCl step gradient ultracentrifugation by mixing 5 ml of 1.5 g/ml CsCl to the bottom of a 38.5 ml ultracentrifuge tube, then add 8 ml of 1.3 g/ml CsCl for the middle layer. Finally add vector suspension to the top. Make sure ultracentrifuge tubes are correctly balanced before proceeding to the next step.
	Centrifuge in SW32Ti rotor for 18 h at 28,000 rpm.
	Place the ultracentrifuge tube above a halogen beam illuminator, identify and collect viral bands with a 18G needle attached to a 5 ml syringe. Transfer into a 14 ml ultracentrifuge tube for linear gradient ultracentrifugation. Fill up the tube with 1.4 g/ml CsCl.
	Centrifuge in SW40Ti rotor for 72 h at 38,000 rpm.
	Place the ultracentrifuge tube above halogen beam illuminator. Identify and collect viral band with a 18G needle attached to a 5 ml syringe.
	Dialyze overnight in a Slide-A-Lyzer cassette in Tris-buffered saline.
	Store vector solution at -80ºC until use.

Supplemental Experimental Procedures

Derivation of iPSC lines

Skin biopsies were obtained from two *CRX*-LCA pediatric patients and healthy parental controls with donor information presented Table S1. Dermal fibroblasts were reprogrammed into iPSCs using a Sendai virus-based approach. Resulting iPSC lines were of normal karyotype and free of mycoplasma contamination with details listed in Table S2.

Cell culture

Retinal organoid differentiation protocol

Differentiation was performed as described in (Kelley et al. 2020) with minor modification of maintaining dissected organoids individually in separate wells of a 96-well ultra-low attachment round-bottom plate (Corning). The detailed procedures are listed in Table S3.

Soluble factors used in retinal differentiation:

ROCK inhibitor (Y-27632 dihydrochloride, Tocris) stock 10 mM, final concentration 10 µM, dilution 1:1000.

Taurine (Sigma-Aldrich) stock 100 mM, final concentration 100 µM, dilution 1:1000.

9-cis-retinaldehyde (Sigma-Aldrich) stock solution 1 mM, final concentration 1 µM or 500 nM, dilution 1:1000 or 1:2000

IGF-1 (Gibco) stock 10 µg/ml, final concentration 20 ng/ml, dilution 1:500

Immunohistochemistry

Retinal organoids were collected using wide-bore pipette tips, washed with phosphate buffer saline (PBS; pH 7.4) and then fixed with 4% paraformaldehyde in PBS (Neuro Technologies) for at least 1 h. Organoids were then washed 3x with PBS and transferred into 15% sucrose solution until they sank to the bottom of the tube. Then the tissue was transferred into 30% sucrose solution and kept overnight. Following these dehydration steps the organoids were placed in Shandon M-1 embedding matrix (Thermo Fisher Scientific) and snap frozen in an ethanol/dry ice bath. Blocks were cryosectioned at 18 µm onto Superfrost Plus glass slides (Thermo Fisher Scientific) in a Microm HM550 cryostat (Thermo Fisher Scientific). Sections were dried before storage at -20°C. For staining, slides were rehydrated in PBS for 15 min and then blocked in 5% donkey serum (Jackson ImmunoResearch), 1% bovine serum albumin (BSA) (Sigma-Aldrich), 0.1% Triton X-100 (Sigma-Aldrich) solution in PBS for at least 1 h. Both primary and secondary antibodies were added in 1% BSA (Sigma-Aldrich), 0.1% Triton X-100 (Sigma-Aldrich) solution in PBS. List of primary antibodies used in the study is presented in Table S4. Sections were incubated with primary antibodies overnight. Slides were washed 5x with PBS, before secondary antibodies were added for 2 h. Slides were washed 3x with PBS then incubated with 4',6-diamidino-2-phenylindole (DAPI) before addition of Fluoromount-G mounting medium (SouthernBiotech) and covering with a microscopy cover glass (VWR). Samples were imaged on a Zeiss 700 confocal microscope (Zeiss) or Leica SP-8 confocal microscope (Leica Microsystems). Images were processed with ZEN Black, ZEN Blue (Zeiss), LAS X (Leica Microsystems), ImageJ and Adobe Photoshop 2020 software packages. Quantifications and fluorescence intensity measurements were performed using ImageJ software (Schroeder et al. 2020). At least three sections from three independent organoids were used for quantification.

Wholemount staining and 3D rendering: Organoids underwent fixation in a 1:4 dilution of BD Cytoperm/Cytofix buffer (BD Biosciences, 554722) for 18 h before blocking in 1x BD Perm wash (BD Biosciences) with 1% normal donkey serum (Jackson ImmunoResearch) for 12 h at room temperature. Primary antibodies (Rhodopsin, L/M Opsin, VSX2, see table above for details) were incubated at a 1:100 dilution at room temperature for 72 h. Secondary donkey anti-mouse, anti-rabbit and anti-sheep conjugated to AlexaFluor 488, 594, 647, respectively, were incubated for 4 h. Following a PBS wash, the organoids were mounted in Ce3D medium to perform tissue clearing (Li, Germain, and Gerner 2017). Imaging was performed with a Leica SP-8 confocal microscope (Leica Microsystems) and reconstructed using Imaris 9.3.0 software package (Oxford Instruments).

Bioinformatic analysis

Bulk RNA sequencing

RNA isolation and library preparation: RNA was isolated from 3 frozen organoids per sample using the QIAGEN RNeasy Kit according to manufacturer's manual (n=3 individual organoids per sample). RNA quality was assessed using the Agilent 2100 Bioanalyzer (Agilent Technologies). Samples (RIN >7) were subsequently used for library generation using the TruSeq Library Preparation Kit (Illumina Inc.). Paired-end sequencing was performed to a length of 125 bases using HiSeq2500 (Illumina Inc.). Human genome reference sequence GRCh38.p7 and Ensembl v94 annotation were used for alignment and quantitation.

Differential analysis and clustering: Transcript-level quantification was performed with kallisto v0.45.0 against Ensembl release 94 then summarized to the gene-level using tximport v1.16.0. Genes with at least 5 count per million (CPM) in all replicates of at least one group (e.g. all three replicates of the D150 control organoids) were retained for downstream analysis. Principal component analysis (PCA) was performed on normalized CPM (log2) values of TMM normalized counts (edgeR v3.20.9) (Robinson, McCarthy, and Smyth 2010). To identify differentially expressed genes (DEGs), the standard edgeR-limma-voom workflow (Ritchie et al. 2015) using empirical Bayes shrinkage of variance was used with at least a 2-fold change and a false discovery rate (FDR) ≤0.05 cutoffs. Genes identified by any of the comparisons between control and LCA model organoids at the same time point were considered DEGs.

Gene ontology analysis: GO enrichment analysis was performed using gProfileR (v 0.7.0) (Reimand et al. 2007) and clusterProfiler (v3.11) (Yu et al. 2012) packages using the Benjamini-Hochberg procedure for multiple-test correction and expressed genes as a custom background. Redundant terms were removed using the strong hierarchical filtering option. Heatmaps of GO term gene lists were generated using the log2 CPM values.

Single cell RNA sequencing

Dissociation

Dissociation performed as described in (Fadl et al. 2020). Retinal organoids were briefly washed in cold 1X HBSS, transferred to a 5 ml polypropylene round-bottom tube and dissociated using a papain-based dissociation protocol. Individual organoids were incubated in 1 ml of digestion solution containing the papain enzyme at 8°C for 1 h followed by a second incubation at 28°C for 10 min. The samples were gently mixed by inverting the tube approximately every 10 min during the 1 h incubation and after 5 min during the 10 min incubation. Following the incubation steps, the organoids, which remained morphologically intact, sank to the bottom of the tube. The supernatant of digestion solution was removed by pipetting. 700 µl of pre-warmed inactivation solution (containing papain inhibitor) was added to the organoids, mechanical trituration was carried out by slow pipetting 10-15 times with a flame polished end glass Pasteur pipette. The solution was aspirated and gently released along the tube wall until the organoids were visibly dissociated and 700 µl of chilled washing solution was layered under the cell suspension. Cells were centrifuged using a swing-bucket rotor at 200 x g for 5 min at 4°C. Supernatant was aspirated, cells resuspended in 500 µl of DPBS containing 0.04% BSA and passed through a 40 µm cell strainer (pluriSelect).

Droplet-based single-cell RNA sequencing

Single cell RNA-seq data were generated using the 10x Genomics Chromium technology. Dissociated organoids cell suspensions were loaded onto the Chromium Single Cell system using the v2 (Chromium Single Cell 3' Library & Gel Bead Kit v2) chemistry. The subsequent steps were performed according to manufacturer's instructions with minor modifications. Cell concentration and viability counts were estimated using the Cellometer Auto 2000 Cell Viability Counter (Nexcelom Bioscience). Suspensions were diluted to achieve 900-1,400 cells/µl. To account for the possibility of cell debris being falsely counted as cells, the cell count was adjusted by subtracting 200-300 cells/µl. In order to capture transcripts from approximately 10,000 cells, approximately 17,000 live cells per sample were loaded into Chromium chip following the Cell Suspension Volume Calculator Table. For every elution step in the cDNA amplification and library construction protocols, the elution volume was increased by 3 µl (Elution solution I or Buffer EB) in order to obtain a supernatant free of contaminating SPRI magnetic beads. 12 cycles were used for the cDNA amplification reaction. Libraries were quantified using the Kapa library quantification kit (Roche) and sequenced on a NovaSeq 6000 (Illumina Inc.).

Computational analysis of single cell data

Raw sequencing output was processed through Cell Ranger pipeline (v3.1.0, 10x Genomics) using *cellranger mkfastq* and *cellranger* count with default settings and the pre-built human (hg19, GRCh38) genome reference. Filtered

expression matrices were further processed using the Seurat package in R (v3.1) (Butler et al. 2018; Stuart et al. 2019) according the publisher's vignette using *sctransform* for normalization, variable gene detection and scaling (compiled October 08th, 2019). Imported data was subsequently processed using default settings for PCA dimensionality reduction, graph-based clustering, and UMAP embedding through the following functions: SCTransfrom() > RunPCA() > RunUMAP() > FindNeighbors() > FindClusters(). We used ElbowPlot() to estimate which principal components to use. Cell type identity was determined for each resulting cluster by assessing expression of known cell type-specific maker transcripts, clusters were then merged into groups reflecting broad retinal cell types.

Preparation of AAV vectors

HEK293 cells were transfected with vector plasmid and pHLP19 and pLAdeno5 helper plasmids using $CaCl_2$ method. Cell pellet was homogenized with a microfluidizer to release AAV particles. Cell debris were eliminated by centrifugation and free DNA removed by 1 h of 100U/ml bezonase treatment. AAV particles were then precipitated in 8% PEG on ice for 2 h. AAV particles pellet was collected by centrifugation and treated with RNaseA for 30 min. at 37°C. Purification of AAV was subsequently conducted using a series of ultracentrifugation steps on CsCl density gradient and dialysis. Titration was performed by qPCR. Detailed procedures are listed in Table S6.

References:

Butler, A., P. Hoffman, P. Smibert, E. Papalexi, and R. Satija. 2018. 'Integrating single-cell transcriptomic data across different conditions, technologies, and species', *Nat Biotechnol*, 36: 411-20.

Fadl, B. R., S. A. Brodie, M. Malasky, J. F. Boland, M. C. Kelly, M. W. Kelley, E. Boger, R. Fariss, A. Swaroop, and L. Campello. 2020. 'An optimized protocol for retina single-cell RNA sequencing', *Mol Vis*, 26: 705-17.

Kelley, R. A., H. Y. Chen, A. Swaroop, and T. Li. 2020. 'Accelerated Development of Rod Photoreceptors in Retinal Organoids Derived from Human Pluripotent Stem Cells by Supplementation with 9-cis Retinal', *STAR Protoc*, 1.

Li, W., R. N. Germain, and M. Y. Gerner. 2017. 'Multiplex, quantitative cellular analysis in large tissue volumes with clearing-enhanced 3D microscopy (C(e)3D)', *Proc Natl Acad Sci U S A*, 114: E7321-e30.

Reimand, J., M. Kull, H. Peterson, J. Hansen, and J. Vilo. 2007. 'g:Profiler--a web-based toolset for functional profiling of gene lists from large-scale experiments', *Nucleic Acids Res*, 35: W193-200.

Ritchie, M. E., B. Phipson, D. Wu, Y. Hu, C. W. Law, W. Shi, and G. K. Smyth. 2015. 'limma powers differential expression analyses for RNA-sequencing and microarray studies', *Nucleic Acids Res*, 43: e47.

Robinson, M. D., D. J. McCarthy, and G. K. Smyth. 2010. 'edgeR: a Bioconductor package for differential expression analysis of digital gene expression data', *Bioinformatics*, 26: 139-40.

Schroeder, A. B., E. T. A. Dobson, C. T. Rueden, P. Tomancak, F. Jug, and K. W. Eliceiri. 2020. 'The ImageJ Ecosystem: open-source software for image visualization, processing, and analysis', *Protein Sci*.

Stuart, T., A. Butler, P. Hoffman, C. Hafemeister, E. Papalexi, W. M. Mauck, 3rd, Y. Hao, M. Stoeckius, P. Smibert, and R. Satija. 2019. 'Comprehensive Integration of Single-Cell Data', *Cell*, 177: 1888-902.e21.

Yu, G., L. G. Wang, Y. Han, and Q. Y. He. 2012. 'clusterProfiler: an R package for comparing biological themes among gene clusters', *Omics*, 16: 284-7.
