# Supplementary material for: Gene Therapy of Dominant CRX-Leber Congenital Amaurosis using Patient Stem Cell-Derived Retinal Organoids
Source: Stem Cell Reports. 2021 Jan 28;16(2):252–63. doi: 10.1016/j.stemcr.2020.12.018 (PMC7878833; doi:10.1016/j.stemcr.2020.12.018)
Supplement: Document S2. Article plus Supplemental Information [file mmc4.pdf]

Stem Cell Reports
Article
OPEN ACCESS

Gene Therapy of Dominant *CRX*-Leber Congenital Amaurosis using Patient Stem Cell-Derived Retinal Organoids

Kamil Kruczek,[1] Zepeng Qu,[1] James Gentry,[1] Benjamin R. Fadl,[1] Linn Gieser,[1] Suja Hiriyanna,[2] Zachary Batz,[1] Mugdha Samant,[1,5] Ananya Samanta,[2] Colin J. Chu,[3] Laura Campello,[1] Brian P. Brooks,[4] Zhijian Wu,[2,6] and Anand Swaroop[1,*]

[1]Neurobiology, Neurodegeneration and Repair Laboratory, National Eye Institute, National Institutes of Health, MSC0610, 6 Center Drive, Bethesda, MD 20892, USA
[2]Ocular Gene Therapy Core, National Eye Institute, National Institutes of Health, Bethesda, MD, USA
[3]Laboratory of Immune System Biology, National Institute of Allergy and Infectious Diseases, National Institutes of Health, Bethesda, MD, USA
[4]Ophthalmic Genetics and Visual Function Branch, National Eye Institute, National Institutes of Health, Bethesda, MD, USA
[5]Present address: All of Us Research Program, Office of the Director, National Institutes of Health, Rockville, MD, USA
[6]Present address: PTC Therapeutics, Inc., South Plainfield, NJ, USA
*Correspondence: swaroopa@nei.nih.gov
https://doi.org/10.1016/j.stemcr.2020.12.018

SUMMARY

Mutations in the photoreceptor transcription factor gene cone-rod homeobox (CRX) lead to distinct retinopathy phenotypes, including early-onset vision impairment in dominant Leber congenital amaurosis (LCA). Using induced pluripotent stem cells (iPSCs) from a patient with *CRX*-I138fs48 mutation, we established an *in vitro* model of *CRX*-LCA in retinal organoids that showed defective photoreceptor maturation by histology and gene profiling, with diminished expression of visual opsins. Adeno-associated virus (AAV)-mediated *CRX* gene augmentation therapy partially restored photoreceptor phenotype and expression of phototransduction-related genes as determined by single-cell RNA-sequencing. Retinal organoids derived from iPSCs of a second dominant *CRX*-LCA patient carrying K88N mutation revealed the loss of opsin expression as a common phenotype, which was alleviated by AAV-mediated augmentation of CRX. Our studies provide a proof-of-concept for developing gene therapy of dominant *CRX*-LCA and other *CRX* retinopathies.

INTRODUCTION

Leber congenital amaurosis (LCA) constitutes a group of rare early-onset retinal dystrophies with severe clinical manifestations, resulting in vision loss during infancy (den Hollander et al., 2008). LCA is genetically heterogeneous with mutations identified in at least 25 genes; a vast majority of these are crucial for photoreceptor development and/or function (Kumaran et al., 2017). Most patients with LCA exhibit autosomal recessive inheritance. Mouse models have been valuable for elucidating disease etiology (Veleri et al., 2015) and for preclinical therapy development, leading to the first U.S. Food and Drug Administration-approved adeno-associated virus (AAV)-based gene therapy of recessive LCA caused by *RPE65* mutations (Apte, 2018). However, it is widely recognized that available animal models do not fully capture complexities of human disease, slowing further clinical translation. No treatment currently exists for other, especially dominant, forms of LCA.

Cone-rod homeobox protein CRX is essential for development of photoreceptors in the retina (Furukawa et al., 1999). CRX controls expression of most rod and cone photoreceptor genes through its interaction with bZIP transcription factor NRL (Mitton et al., 2000) and/or an intricate network of regulatory proteins (Hennig et al., 2008). Heterozygous mutations in *CRX* cause early-onset retinal dystrophies with extensive phenotypic heterogeneity, with rare reports of biallelic variants in LCA (Huang et al., 2012; Hull et al., 2014; Ibrahim et al., 2018; Rivolta et al., 2001; Swaroop et al., 1999). Notably, a majority of reported dominant LCA can be attributed to *CRX*. Disease-causing *CRX* mutations can either reduce the transcriptional activity or impart a gain of function effect (Tran et al., 2014). Studies in animal models revealed a severe phenotype associated with dominant frameshift mutations in *Crx* compared with loss-of-function alleles (Roger et al., 2014; Tran et al., 2014).

We previously described clinical presentations of two pediatric patients carrying c.G264T (p.K88N)- or c.413delT (p.I138fs48) dominant *CRX* mutations manifested as LCA, with a complete loss of light-evoked responses in electroretinogram recordings (Nichols et al., 2010). Importantly, retinal imaging revealed preservation of outer nuclear layer, suggesting presence of viable but nonfunctional photoreceptors, which could be potential targets for gene therapy. Molecular analysis of mutant alleles demonstrated differential interaction with NRL *in vitro*, suggesting interference with gene regulation in rod photoreceptors (Nichols et al., 2010). Although animal models phenocopy human *CRX* retinopathies (Roger et al., 2014; Tran et al., 2014) and can provide proof-of-concept for treatment (Watanabe et al., 2013), there are significant differences in development, cell-type composition, and

This is an open access article under the CC BY license (http://creativecommons.org/licenses/by/4.0/).

molecular profiles of human retina (Hoshino et al., 2017). Therefore, we decided to take advantage of patient-derived induced pluripotent stem cells (iPSCs) to create a human model for developing therapeutic paradigms for *CRX*-LCA.

Directed differentiation of iPSCs into three-dimensional (3D) retinal organoids has enabled the modeling of retinopathies in patient-specific genetic background (Kruczek and Swaroop, 2020). Next generation sequencing technologies have provided a detailed comparison of developing retinal organoids with human retina (Collin et al., 2019; Cowan et al., 2020; Hoshino et al., 2017; Kaya et al., 2019; Kim et al., 2019) and permit evaluation of major retinal cell types in a disease context. In this study, we established a retinal organoid model of *CRX*-LCA from patient-derived iPSCs and demonstrate perturbation in molecular phenotype of photoreceptors, including diminished expression of visual opsins, in concordance with clinically observed loss of light responses. We also show partial restoration of both rod and cone gene expression by delivering correct CRX transgene driven by a human *CRX* promoter via an AAV vector. Our studies provide a path forward for treatment of dominant *CRX*-LCA by gene augmentation.

RESULTS

Skin biopsies of two LCA patients carrying dominant c.G264T (p.K88N) or c.413delT (p.I138fs48) mutation in the *CRX* gene (Figure 1A), as well as unaffected familial controls (Table S1), were used to derive iPSC lines, which demonstrated normal karyotype and typical features of stem cells (Figure S1A, S1B, S5A, and S5B; Table S2). Retinal organoids were differentiated using a previously published protocol (Kaya et al., 2019; Zhong et al., 2014) (Figure 1B). Pairwise comparisons of organoids derived from each patient to respective healthy familial control were performed in all presented data. Both patient and control cell lines formed morphologically similar retinal neural epithelia (Figures S1C and S5C).

Given that a frameshift mutation showed a particularly severe retinal phenotype in Crx^{Rip} mouse model (Roger et al., 2014), we first examined the organoids derived from c.413delT(p.I138fs48) iPSCs. Retinal organoids exhibit three stereotypical stages of differentiation (Capowski et al., 2019; Kaya et al., 2019) corresponding to developmental epochs in human fetal retina (Hoshino et al., 2017). At stage 1, neuroepithelia of control and patient organoids were indistinguishable (Figures S1C–S1E). During stage 2, CRX-expressing photoreceptors aligned at the apical side of neural retina in both control and patient organoids (Figure 1C) and stained positive for early photoreceptor marker, Recoverin (Figure 1D). Immunoblotting of day 90 organoid protein extracts revealed a truncated CRX protein (referred as I138fs) in patient samples (Figure 1E). Quantification of the protein bands indicated higher levels of total CRX in patient samples with a significant proportion contributed by the mutant allele (Figure 1F). The phenotypic distinction between the control and patient organoids was evident at stage 3, when photoreceptors began to form outer segment-like structures visible as an apical "brush" in light microscopy. While clearly detectable in control organoids, formation of these structures was abrogated in CRX-I138fs patient organoids (Figure 2A; details of sampling size used for quantifications and description of statistical analyses are provided in methods and figure legends). As early as day 125, control organoids showed patches of developing rods as revealed by immunostaining of the rod visual pigment Rhodopsin, a transcriptional target of CRX. However, Rhodopsin staining was virtually absent in CRX-I138fs organoids (Figure 2B). Similarly, cone photoreceptor opsins displayed altered expression. S Opsin immunostaining was delayed compared with the control (Figures S2A–S2F), whereas the number of L/M Opsin + cones was substantially reduced (Figure 2C). 3D renderings of organoids at day 200 confirmed the diminished opsin staining (Figure 2D, Videos S1 and S2). Another outer segment component, Peripherin2, was also dramatically reduced in CRX-I138fs organoids (Figure 2E). In contrast, developing ribbon synapses appeared unaffected (Figures S2G and S2H). Transcriptome analysis at key stages of organoid differentiation, days 90, 125, 150, and 200, identified differential expression of multiple transcripts associated with photoreceptor function (Figure 2F), consistent with the established role of CRX (Furukawa et al., 1999; Hennig et al., 2008). Rod and L/M cone opsins (*RHO*, *OPN1MW*) were the most significantly diminished transcripts in CRX-I138fs organoids (Figure 2F), in accordance with immunostaining results (Figures 2B and 2C). Thus, histological and transcriptome analyses of retinal organoids showed aberrant photoreceptor development due to the I138fs mutation in *CRX*.

Next, we asked whether gene augmentation with normal CRX might alleviate the disease phenotype by competing with the mutant isoform. First, we tested transduction with two commonly used AAV capsid serotypes AAV2 and AAV8 with cytomegalovirus (CMV) promoter driving a GFP reporter. AAV2 capsid showed much higher proportion of cells expressing both GFP and CRX in organoids at day 150, 10 days post vector addition at day 140 (Figure 3A). We then tested human *CRX* promoter sequences for driving transcription in retinal cells that normally express the *CRX* gene. Three promoter fragments were combined to generate a shorter composite promoter (see Table S5), which was placed upstream of GFP and packaged into AAV2 capsid (Figure 3B). As compared with

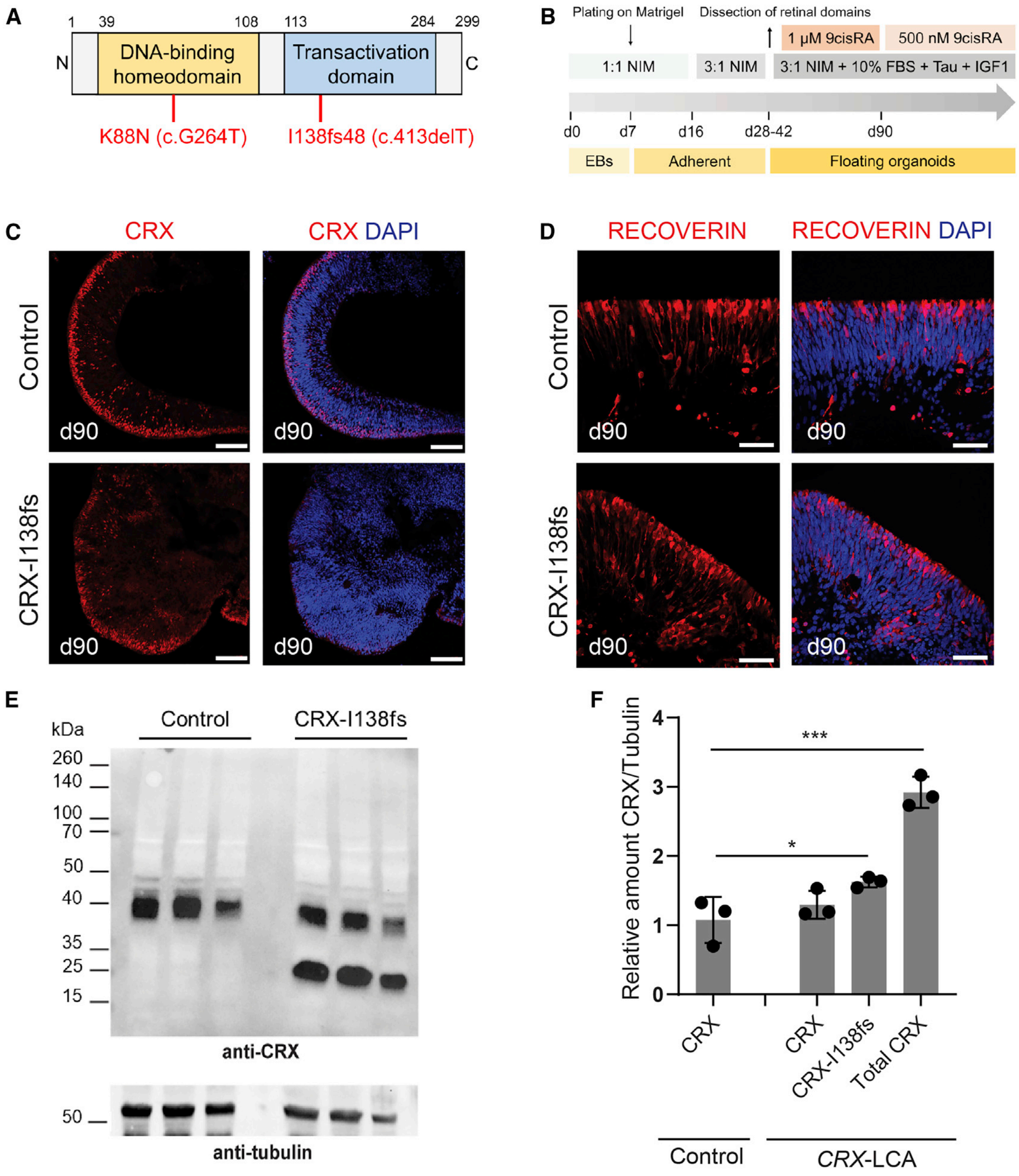

Figure 1. Differentiation of Photoreceptors in CRX-I138fs Retinal Organoids

(A) Schematic representation of CRX protein, showing domain structure and the positions of dominant pathological mutations in the study patients. Number of amino acid residue is indicated over the bar. N and C indicate amino- and carboxyl-terminal of the CRX protein.

(B) An overview of the retinal organoid differentiation protocol.

(C) CRX expression in developing photoreceptors at day 90. CRX antibody labeled apically aligning developing photoreceptors in both control and patient organoids. Scale bar, 50 μm. Note a more diffuse pattern in the patient sample. Nuclei were stained with DAPI (4′,6-diamidino-2-phenylindole).

(D) Developing photoreceptors of both genotypes also express the marker protein Recoverin. Scale bar, 20 μm.

(E) Immunoblot analysis of organoid protein extracts at day 90. Molecular mass markers (kDa) are indicated on the left. Protein samples from control and patient stem cell-derived organoids (n = 3 biological replicates containing three organoids each) were run in separate lanes. Tubulin was used as a loading control. A smaller molecular weight band, which corresponds to the mutant CRX-I138fs48 isoform, is evident in the patient samples.

(F) Densitometry quantification of CRX protein bands normalized to the Tubulin loading control. Total CRX protein is significantly more abundant in CRX-I138fs patient samples. Mean ± SD plotted, *$p < 0.05$, ***$p < 0.001$, one-way ANOVA.

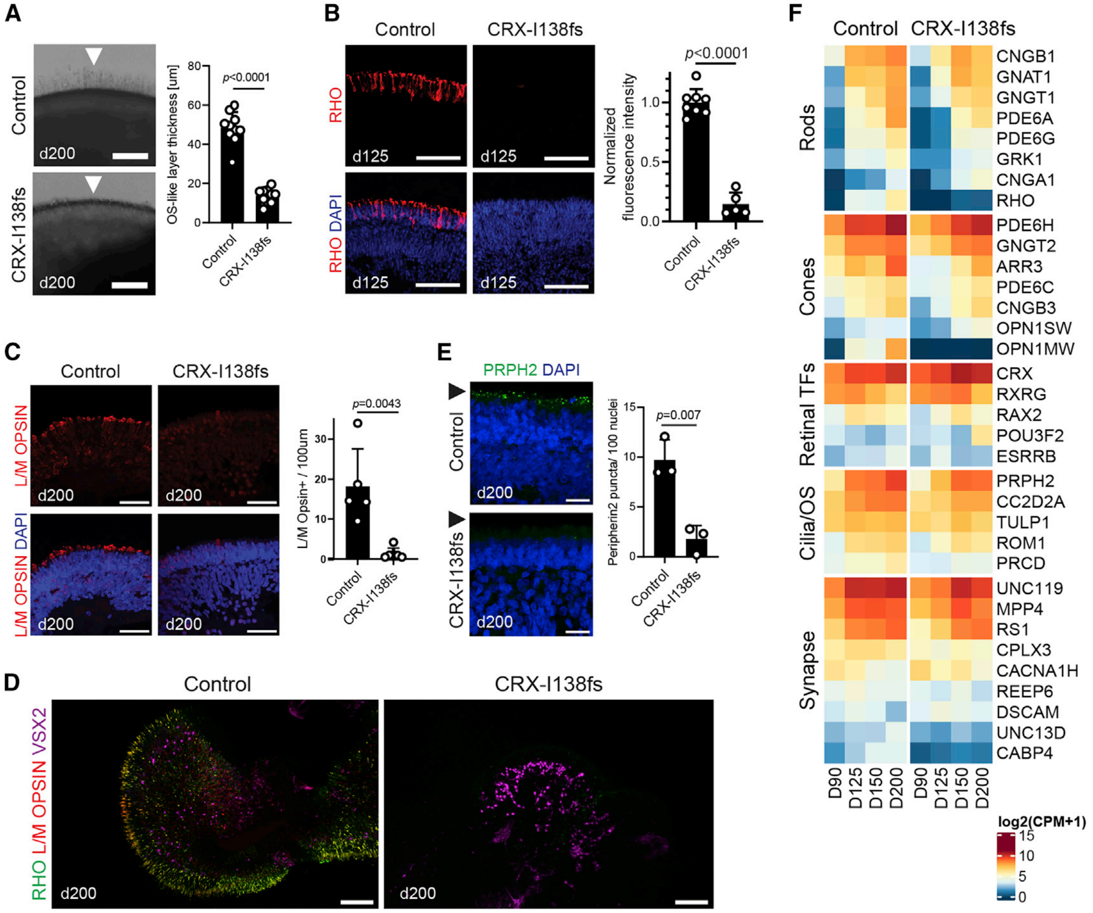

Figure 2. Impaired Photoreceptor Maturation in *CRX*-LCA Retinal Organoids

(A) Brightfield images of apical aspect of organoid neural retina. Scale bar, 400 μm. Note loss of brush-like outer segment (OS) structures in patient (arrowheads).

(B) Rhodopsin (RHO) immunostaining at day 125 and fluorescence intensity quantification. Nuclei were stained with DAPI (4′,6-diamidino-2-phenylindole). Scale bar, 20 μm.

(C) L/M Opsin immunostaining with quantification of positive cells. Scale bar, 20 μm.

(D) Representative whole-mount confocal images of organoids at day 200 immunostained for RHO, L/M Opsin, and Visual System Homeobox 2 (VSX2). Scale bar, 160 μm.

(E) Peripherin2 (PRPH2) staining and puncta quantification. Scale bar, 10 μm.

(F) Heatmap comparing expression of genes (bulk RNA-seq) across organoid development (days 90, 125, 150, and 200). Values are shown as log2(CPM+1). TFs, transcription factors; OS, outer segment. Number of organoids per genotype analyzed for quantifications: (A) control n = 10, patient n = 10; (B) control n = 8, patient n = 5; (C) n = 5 control, n = 3 patient; (E) control n = 3, patient n = 3. Values represent mean ± SD with individual data points plotted (three sections per organoid). Statistical significance was determined by Student's t test; p values indicated.

transduction with ubiquitously expressed CMV promoter, *CRX* promoter-driven GFP signal localized to a distinct apical lamina corresponding to the photoreceptor layer in both control and CRX-I138fs patient organoids (Figure 3B). The final therapeutic construct contained this *CRX* composite promoter driving human *CRX* expression (Figure 3C). This AAV-*CRX* vector was added to patient organoids at day 120, and the samples were analyzed at days 150 and 180. Transduction with the vector increased CRX mRNA and protein in treated organoids (Figures S3A–S3D). Rhodopsin and L/M Opsin expression were used as surrogates for treatment, given their near absence in patient organoids. Retinal organoids transduced with 1×10^{11} vector genomes (vg) began to show rescue of Rhodopsin expression by day 150 (Figures S3E and S3F), which became more widespread by day 180 (Figure 3D). Quantification of Rhodopsin immunolabeling intensities confirmed higher fluorescence compared with untreated

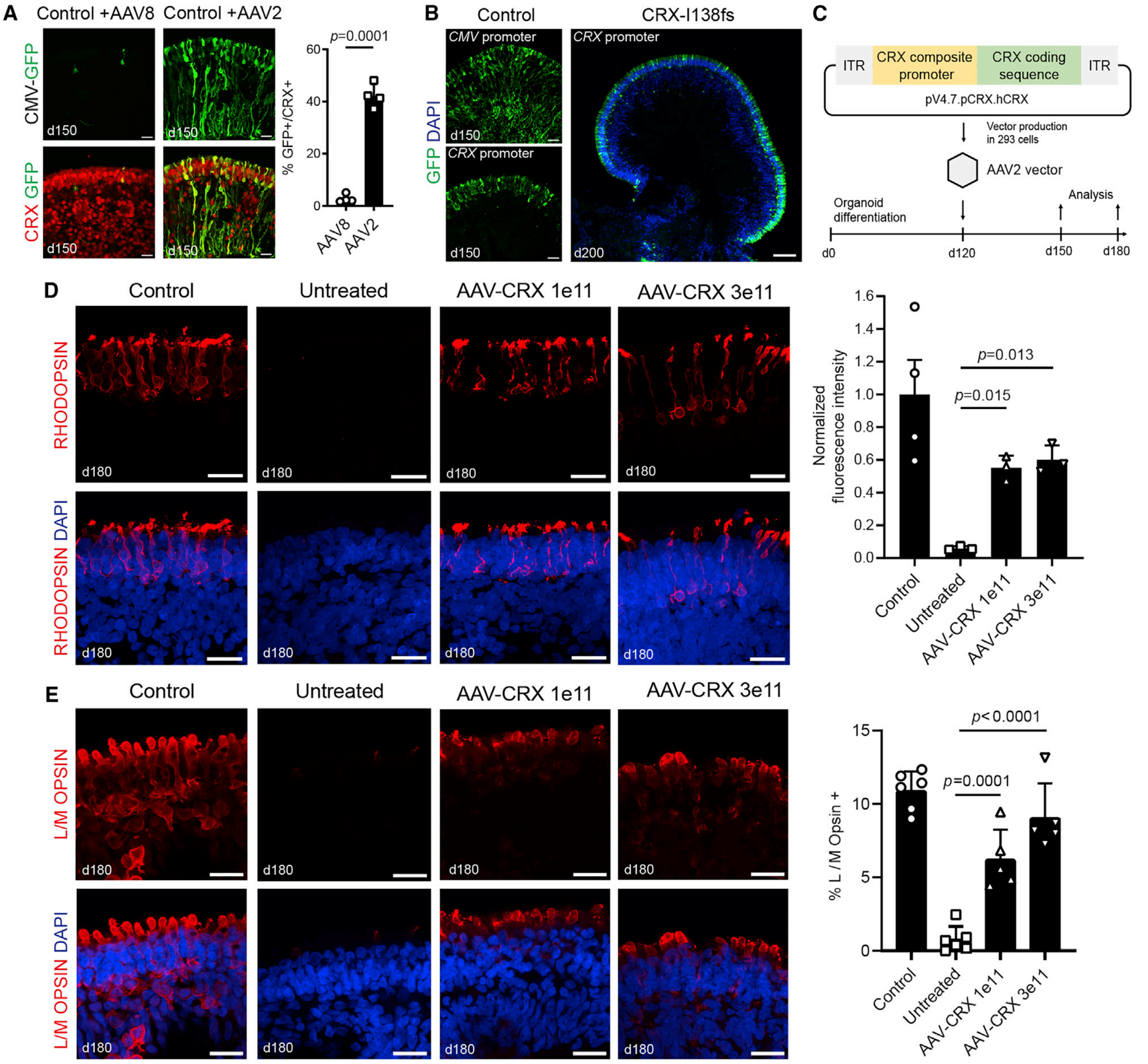

Figure 3. AAV-Delivered Gene Augmentation for *CRX*-LCA Caused by I138fs Mutation

(A) Transduction of CRX-expressing cells by AAV2 and AAV8 serotypes. Quantification of percent of AAV-delivered GFP reporter in CRX-positive cells on control organoid cryosections. Scale bar, 20 μm. For both serotypes, n = 4 organoids, three sections each averaged; mean ± SD, statistical significance by Student's t test, p value indicated.

(B) Promoter testing in retinal organoids. Comparison of GFP reporter expression driven by CMV or CRX promoters in day 150 control organoids (left; scale bar, 20 μm). Note broad expression using CMV as compared with localization primarily to outer organoid layer with CRX promoter. GFP expression driven by CRX promoter in CRX-I138fs48 patient organoids at day 200 (right). Nuclei were stained with DAPI (4′,6-diamidino-2-phenylindole). Scale bar, 200 μm.

(C) Schematic representation of the therapeutic AAV vector design and testing in retinal organoids.

(D and E) Rhodopsin (D) and Cone L/M Opsin (E) staining in healthy control, and untreated and AAV-treated patient retinal organoids at day 180. Two doses of AAV-CRX vector were tested (1×10^{11} and 3×10^{11} vg per organoid). Scale bar, 20 μm. Efficiency of treatment was evaluated with quantification of Rhodopsin expression rescue and percentage of L/M Opsin + cones. Number of organoids analyzed for quantifications: (D) control n = 4, untreated and AAV-treated n = 3 each dose; (E) control and untreated n = 6; AAV-treated n = 5 each dose; three sections each. Values represent mean ± SD with individual data points plotted. Statistical significance was determined by one-way ANOVA; p values indicated.

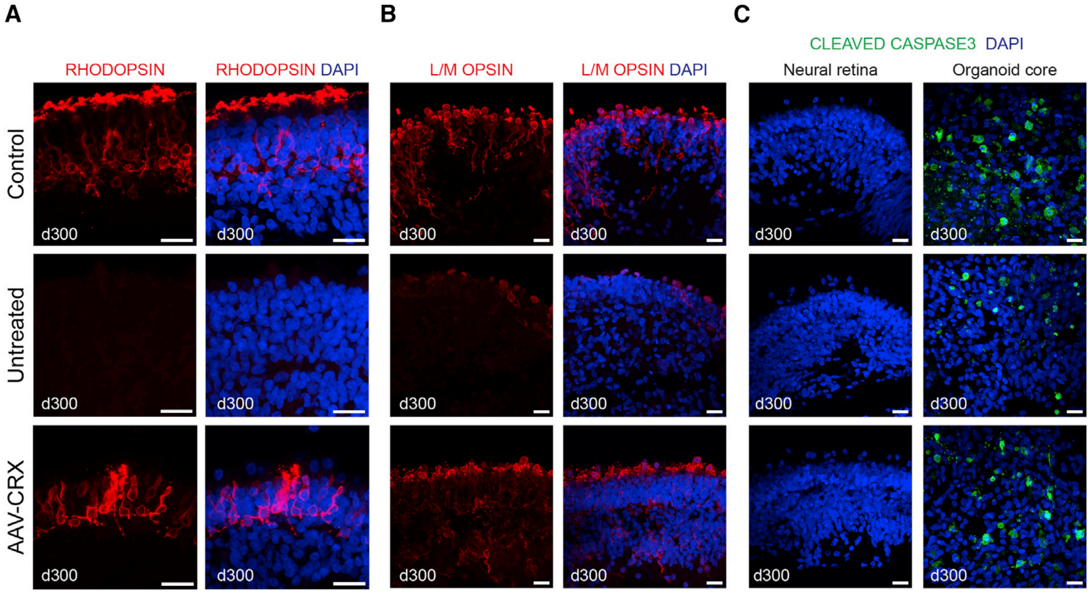

Figure 4. Long-Term Effects of AAV-CRX Gene Augmentation in CRX-I138fs Retinal Organoids
Retinal organoids were transduced at day 120 with AAV-CRX vector at 1×10^{11} vg per organoid and examined 180 days later, at day 300 in culture. Immunostaining for (A) Rhodopsin, (B) L/M Opsin, (C) cleaved Caspase3. Nuclei were counterstained with DAPI. Note the continued presence of cells with rescued expression of opsins. Prolonged CRX expression does not appear to induce apoptotic cell death in the photoreceptor layer, where cleaved Caspase3 staining is absent (C, left), in contrast to the core region of the organoid showing extensive apoptosis in extended culture (C, right). Scale bar in all images, 20 μm.

patient iPSC-derived organoids with both 1×10^{11} vg and 3×10^{11} vg doses and was almost half of signal intensity measured for the healthy control (Figure 3D). Fluorescence intensity levels were similar at both vector doses, suggesting that 1×10^{11} vg per organoid is sufficient for substantial rescue of Rhodopsin expression. Treatment also partially restored L/M Opsin expression (Figures 3E, S3G, and S3H). Rescue effect persisted at least for 6 months after treatment (day 300) with both Rhodopsin and L/M Opsin-positive cells still detectable (Figures 4A and 4B). Long-term expression of CRX did not lead to activation of the apoptotic marker Caspase3 in AAV-treated organoid neural retina (Figure 4C).

To decipher and validate the impact of CRX-I138fs mutation on specific cell types within retinal organoids, we performed single-cell RNA-sequencing (scRNA-seq) using 10x Genomics platform. Control, untreated CRX-I138fs and AAV-treated organoids were dissociated using a papain-based method (Fadl et al., 2020) at day 200 yielding 40,712 single-cell transcriptional profiles. Data processing using Seurat package identified cell clusters, which were assigned to known retinal cell types and visualized using UMAP dimension reduction (Figure 5A and S4A–S4C). In this representation, major retinal cell classes (apart from ganglion cells) emerge from centrally located undifferentiated cells (Figure 5A). Cell-type distribution was similar across the three sample origins (Figures 5A and S4B). Rods and cones formed well-defined differentiation trajectories in this manifold and were identified by expression of both common (*CRX*, *RCVRN*) and subtype-specific markers (rod: *GNGT1*, *GNAT1*; cone: *ARR3*, *PDE6H;* Figures 5B, S4D,and S4E). As predicted, CRX transcripts increased in photoreceptors after AAV-CRX transduction (Figure 5C). Rod and cone expression profiles could be clearly separated based on control or patient sample origin, whereas AAV-treated cells occupied the space in between (Figures 5D, 5E, 5G, and 5H). This shift was particularly evident by plotting the origin of most cells across hexagonal bins (Figures 5E and 5H). ScRNA-seq detected partially rescued expression of opsins following AAV treatment (*RHO*, *OPN1MW3*, Figures 5F and 5I; *OPN1MW3* was the most significantly dysregulated of three medium wavelength opsin genes *OPN1MW1-3*), as well as of other rod- and cone-specific transcripts (Figures S4D and S4E; for each gene adjusted p value <0.05, nonparametric Wilcoxon rank-sum test with Bonferroni correction; minimum percent expressed = 10% cells, minimum log fold change = 0.25). CABP4, a retinal disease gene and direct transcriptional target of CRX (Assawachananont et al., 2018) showed a similar trend (Figures S4F and S4G). Thus, single-cell analysis confirmed treatment effect of AAV-mediated overexpression of normal CRX.

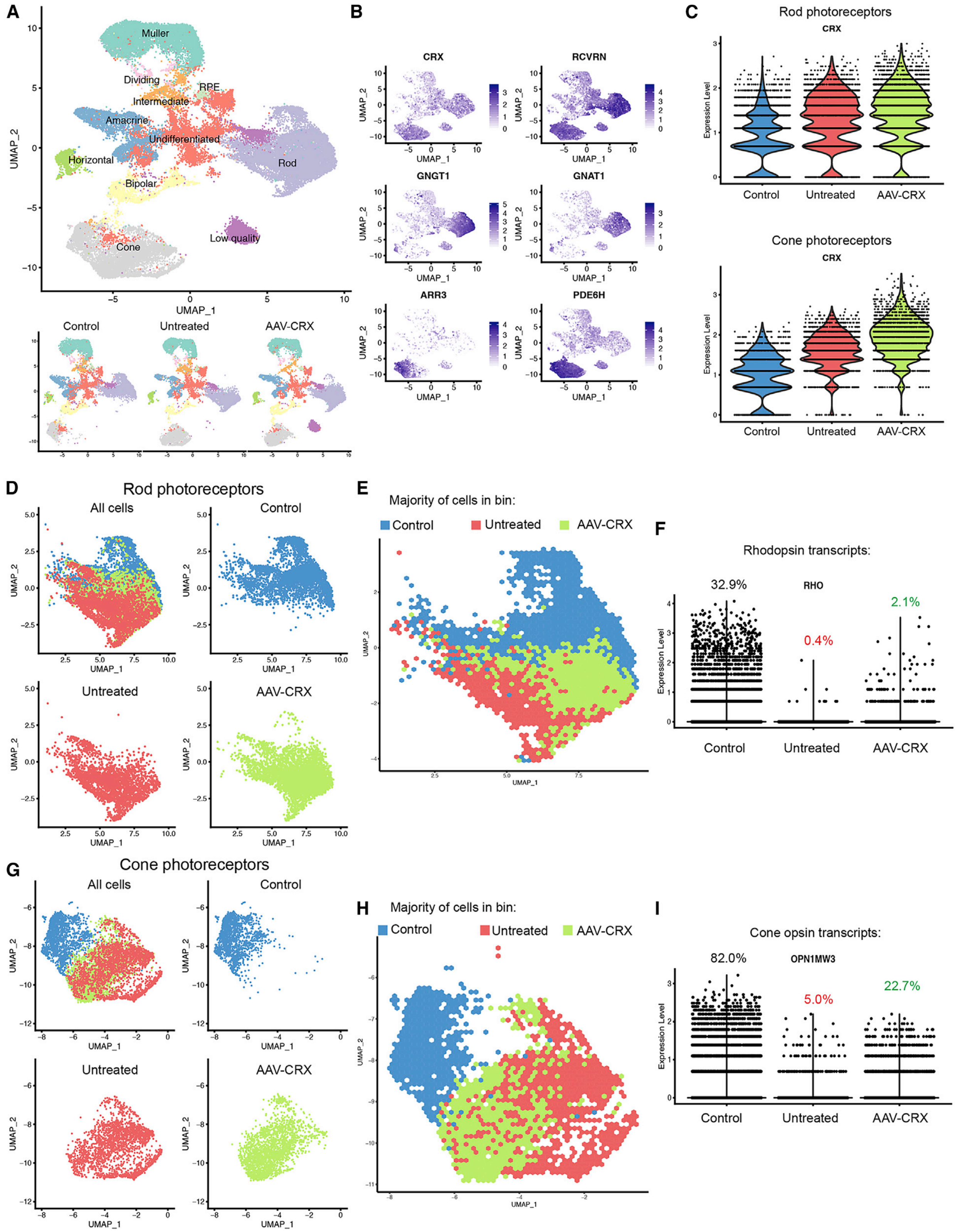

(legend on next page)

To determine whether the observed phenotypes and rescue were mutation-specific or could be generalized to other cases of dominant *CRX*-LCA, we examined organoids with the CRX K88N mutation. As for the frameshift mutation, no differences in morphology and expression of key retinal markers were evident at stage 1 or 2 of organoid differentiation (Figures S5C–S5E). However, outer segment-like structures were less developed at stage 3 in patient iPSC-derived organoids compared with the control (Figures 6A and 6B). Immunostaining showed the presence of CRX and Recoverin, but severely diminished Rhodopsin and L/M Opsin staining in CRX-K88N organoids (Figure 6C). Transcriptome analyses at days 120 and 200 revealed delayed upregulation of many photoreceptor-specific genes (Figure 6D) and confirmed the loss of Rhodopsin and L/M Opsin expression (*RHO*, *OPN1MW2*; Figure 6D). Treatment of CRX-K88N organoids with AAV-CRX vector increased CRX levels (Figure S6A), partially rescued Rhodopsin and L/M Opsin expression (Figures 6E–6G) and reduced abnormal S Opsin levels (Figure S6B), with a modest induction of rod visual arrestin and synaptic proteins (Figure S6B). Thus, CRX-K88N organoids showed a similar phenotype to CRX-I138fs, and AAV gene therapy was able to restore expression of CRX target genes.

DISCUSSION

Dominant diseases represent a considerable challenge for developing gene therapy because of the presence of a mutant allele that interferes with normal function (Ahmed et al., 2019; Collin et al., 2019). Overexpression of a normal copy of Rhodopsin or VSX2 has been shown to partially correct defects in mouse or retinal organoid models of respective dominant disease (Mao et al., 2011; Phillips et al., 2014). We previously observed reexpression of Rhodopsin following electroporation of wild-type *Crx* into the retina of a mouse model harboring a spontaneous dominant frameshift mutation in the *Crx* gene (Roger et al., 2014). Building on that observation, we tested the hypothesis that overexpression of a correct *CRX* cDNA increasing the ratio of normal to mutant protein (gene augmentation) may rescue defects in dominant human *CRX* retinopathies. We developed retinal organoid models of dominant *CRX*-LCA from patients with two different mutation types and demonstrate the feasibility of using AAV-delivered *CRX* as a therapeutic strategy. We note that patient iPSC-derived organoids have recently been used to provide proof-of-concept for AAV-mediated gene therapy of recessive X-linked retinitis pigmentosa caused by mutations in *RP2* (Lane et al., 2020).

CRX-LCA patient organoids showed impaired maturation of photoreceptors with particularly disrupted expression patterns of visual opsins. Rhodopsin and L/M cone opsins were almost undetectable, whereas S Opsin expression was initially delayed but recovered later, consistent with findings in animal models (Roger et al., 2014; Tran et al., 2014). We observed increased total CRX protein levels in organoids of the patient with I138fs mutation, especially of the mutant isoform, whereas K88N organoids showed reduced overall levels of CRX protein. These findings suggest distinct potential downstream impacts of the dominant alleles: (1) competition of mutant protein with normal CRX for target binding sites, (2) prolonged occupancy of mutant protein at binding sites preventing the correct form from associating to the target DNA, and (3) disruption of stoichiometric interactions with other retinal transcription factors, such as NRL. The contribution of these mechanisms may vary depending on a specific mutation, but as we demonstrate, convergent molecular pathology such as disruption of opsin expression is evident.

We took advantage of scRNA-seq to determine disease-associated changes in photoreceptor subtypes as well as to evaluate treatment effect following gene therapy. We observed clear separation of patient cones and rods from control populations and reduced expression of cell-type-specific genes. In rods, partial shift toward control population with AAV treatment is associated with higher rescue of Rhodopsin expression in a subset of transduced cells, validating histological observations. Importantly, we detected

Figure 5. Altered Gene Expression Patterns and AAV Treatment Effects in Cone and Rod Photoreceptor Subtypes of CRX-I138fs48 Patient Retinal Organoids at day 200

(A) Top: UMAP representation of the single-cell RNA-seq dataset (n = 40,712 transcriptomes) displaying major cell types (annotated using known cell-type marker genes). Bottom: UMAP plots showing the distribution of cells of control (n = 2 biological replicates, 4 organoids each), and untreated and AAV-CRX (n = 2 biological replicates, 3 organoids each) organoid samples.

(B) Expression of photoreceptor cell-type- (*CRX*, *RCVRN*) and subtype-specific markers (rods: *GNGT1*, *GNAT1*; cones: *ARR3*, *PDE6H*).

(C) Violin plot profiles of CRX expression levels in rods and cones. Note increased expression with AAV-CRX gene augmentation.

(D–I) Treatment effects in rod (D–F) and cone (G–I) photoreceptor subtypes. (D and G) UMAP plots showing the distribution of rod and cone cells by sample origin (control, blue; untreated, red; AAV-CRX, green). (E and H) Hexagonal bin plots illustrating identity of cell origin (coloring each hexagon according to the origin of the majority of cells it covers). Note placement of AAV-CRX treatment between patient and control sample majority areas. (F and I) Opsin transcript reads in the different samples visualizing increased expression in patient-derived samples following treatment. Percentages of cells of each origin in which transcript reads were detected are indicated.

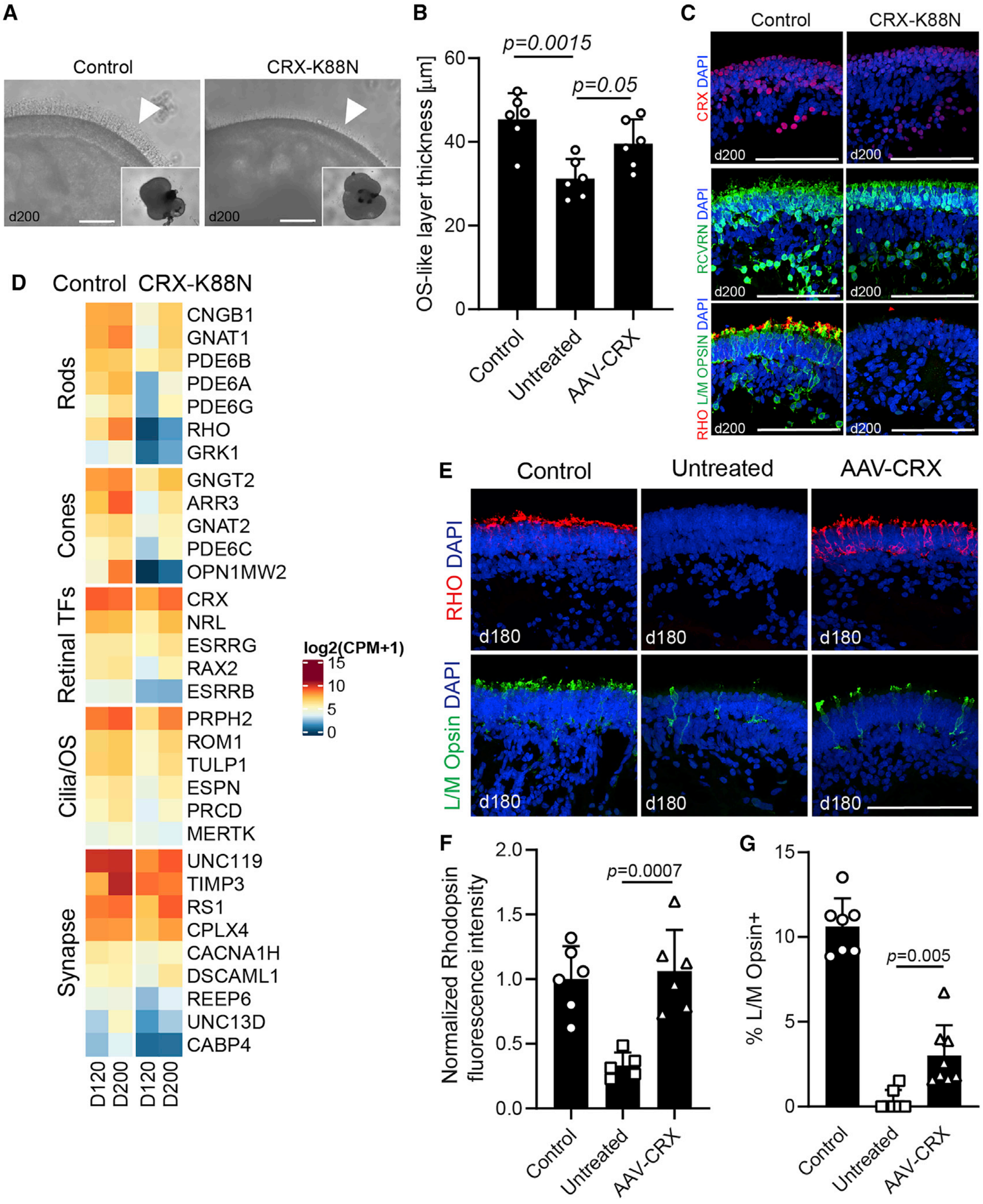

Figure 6. Disease Phenotype and Gene Augmentation Therapy of CRX-K88N Patient Retinal Organoids

(A) Brightfield images of control and CRX-K88N patient organoids showing reduced outer segment (OS) apical "brush" layer (arrowheads).

(B) Quantification of the outer segment-like layer thickness; n = 6 organoids per group, three sections each; mean ± SD, p values from one-way ANOVA.

(C) Immunostaining of organoids at day 200 for CRX, Recoverin, Rhodopsin and L/M Opsin. Note diminished Rhodopsin and L/M Opsin staining in patient-derived organoids. Nuclei were stained with DAPI (4′,6-diamidino-2-phenylindole). Scale bar, 100 µm.

(D) Heatmap comparing expression of genes (bulk RNA-seq) of day 120 and day 200 organoids. Expression of many photoreceptor-specific transcripts is either delayed or reduced in CRX-K88N patient samples. Normalized log2(CPM+1) values plotted. TFs, transcription factors; OS, outer segment.

(E) AAV treatment assessment by immunostaining. Immunoreactivity for both Rhodopsin and L/M Opsin is partially restored following AAV treatment. Scale bar, 100 µm.

(legend continued on next page)

continued rescue of Rhodopsin and L/M Opsin at 6 months posttreatment without any indication of apoptosis. Further investigations are needed to define optimal dosing as well as time window for gene therapy. In addition, we may require *in vivo* model(s) to fully evaluate the long-term impact of augmented CRX expression. In any event, single-cell transcriptomics offers a powerful approach to assess treatment approaches for retinopathies.

The difference in rescue of L/M Opsin expression was evident between patient organoids of the two genotypes, suggesting differential interaction of mutant CRX with binding partners in cones. While restoration of cone gene expression was clearly evident in our study, further enhancement is likely by selecting a more potent capsid serotype targeting cones (Gonzalez-Cordero et al., 2018). Treatment of cone photoreceptors is of relevance because mutations in *CRX* also cause milder adult-onset cone-rod dystrophies and macular dystrophies, which could potentially benefit from gene therapy treatment described here.

In conclusion, we used retinal organoids as a platform to examine disease mechanisms and design gene augmentation therapy for dominant *CRX*-LCA. We suggest that our experimental strategy should be applicable in developing effective treatments for rare, and even dominant, inherited diseases of the retina and other parts of the central nervous system.

EXPERIMENTAL PROCEDURES

Retinal organoids from patient-derived iPSCs

Skin biopsies were obtained from two CRX-LCA pediatric patients and respective healthy parental controls with informed consent (Table S1). Dermal fibroblasts were reprogrammed to generate iPSCs using a Sendai virus-based approach. Resulting iPSC lines were of normal karyotype and free of mycoplasma contamination (Table S2). iPSCs were differentiated following an established protocol (Kaya et al., 2019) with a minor modification of culturing dissected retinal organoids individually in a 96-well plate format (Table S3). Histology was performed as described in (Shimada et al., 2017), a list of all primary antibodies used for immunofluorescent staining is provided in Table S4. Images were obtained on Zeiss 700 confocal microscope.

Next generation sequencing

RNA was isolated from three pooled frozen organoids per sample using the QIAGEN RNeasy Kit according to manufacturer's manual. RNA quality was assessed using the Agilent 2100 Bioanalyzer (Agilent Technologies). Samples (with RNA integrity number >7) were used for library generation using the TruSeq Library Preparation Kit (Illumina Inc). Sequencing and bioinformatic analysis were performed as described (Kaya et al., 2019).

For single-cell RNA-seq, organoids were dissociated following a papain-based method and subjected to single-cell isolation and sequencing as described (Fadl et al., 2020). Processing and analysis of single-cell transcriptomes using Seurat package is detailed in Supplemental information.

Cloning of a composite human *CRX* promoter

Three fragments from the human CRX 5′ untranslated region (NCBI: NG_008605.1) were amplified from human genomic DNA and ligated to produce a 631 base pair promoter sequence (Table S5). In this promoter, 189 nucleotides correspond to positions 3,085–3,274, 69 correspond to 3,323–3,392, and 361 correspond to 4,808–5,169 of the reference. This promoter element contains first exon of the human *CRX* gene (nucleotides 4,999–5,169).

Statistical analysis

At least three independently cultured organoids (individual wells of a 96-well plate) were used for quantification. Three differentiation batches were analyzed to verify disease phenotypes. In histological analyses, data from three sections were averaged to account for regional variation within individual organoids. With respect to next generation sequencing and single-cell sequencing, three organoids were pooled to obtain one sample. GraphPad Prism version 8.0 software was used to plot data and perform statistical analysis. For two group comparisons two-tailed Student's t test was used. For multiple groups, one-way ANOVA analysis was used with either Dunnett's or Tukey's post hoc tests. Quantitative data are presented as means ± standard deviation (SD). Exact p values are reported in the figures, and p values of <0.05 were considered statistically significant.

Study approval

Skin biopsies were obtained from the patients and unaffected family members at the National Eye Institute Clinical Center under an institutional review board-approved protocol #15-EI-0128, NCT01432847.

Detailed description of all experimental methods is provided in Supplemental information.

ACCESSION NUMBERS

The accession number for the RNA-seq data reported in this paper is GEO: GSE153101.

SUPPLEMENTAL INFORMATION

Supplemental information can be found online at https://doi.org/10.1016/j.stemcr.2020.12.018.

(F) Quantification of Rhodopsin fluorescence intensity in AAV-treated retinal organoids. Control n = 6, untreated n = 5, and AAV-treated n = 6 organoids; three sections each; mean ± SD, p values from one-way ANOVA.
(G) Quantification of the percentage of L/M Opsin + cones in AAV-treated retinal organoids. Control n = 7, untreated n = 7, and AAV-treated n = 8 organoids; three sections each; mean ± SD, p values from one-way ANOVA.

AUTHOR CONTRIBUTIONS

Conceptualization, A. Swaroop, K.K.; Methodology, Investigation, Validation and Analysis, K.K., Z.Q., L.C., C.J.C., S.H., M.S., A.Samanta; Data curation and analysis, and Visualization, J.G., B.R.F., Z.B.; Resources, L.G., S.H., Z.W., B.P.B.; Writing – Original draft, K.K., A. Swaroop; Writing – Review & Editing, all authors; Project administration and supervision, Funding acquisition, A. Swaroop.

CONFLICTS OF INTEREST

K.K., S.H., Z.W., and A.S. are inventors on a patent application "Gene therapy for treatment of CRX-associated retinopathies," submitted by the National Eye Institute. Z.W. is now an employee of PTC Therapeutics. Other authors declare no conflict of interest.

ACKNOWLEDGMENTS

We are grateful to the patients and their families for tissue donations, which enabled this study. We thank Jeanette Beers at the NIH/National Heart, Lung, and Blood Institute iPS and Genome Engineering Core Facility for generating iPSC lines, Sandra Burkett at the Molecular Cytogenetics Core Facility, National Cancer Institute Center for Cancer Research for karyotyping, and Milton A. English and Charles Drinnan for fibroblast cell line generation and stem cell maintenance. This research was supported by the Intramural Research Programs of the National Eye Institute (ZIAEY000450 and ZIAEY000546 to A. Swaroop) and, in part, by the National Institute of Allergy and Infectious Diseases, National Institutes of Health. We are grateful to Opsomai Foundation (Italy) for a generous donation, which partly funded this work. This work used the high-performance computational capabilities of the NIH Biowulf Linux cluster (http://hpc.nih.gov).

Received: August 13, 2020
Revised: December 27, 2020
Accepted: December 28, 2020
Published: January 28, 2021

REFERENCES

Ahmed, C.M., Dwyer, B.T., Romashko, A., Van Adestine, S., Park, E.H., Lou, Z., Welty, D., Josiah, S., Savinainen, A., Zhang, B., et al. (2019). SRD005825 acts as a pharmacologic chaperone of opsin and promotes survival of photoreceptors in an animal model of autosomal dominant retinitis pigmentosa. Transl. Vis. Sci. Technol. *8*, 30.

Apte, R.S. (2018). Gene therapy for retinal degeneration. Cell *173*, 5.

Assawachananont, J., Kim, S.Y., Kaya, K.D., Fariss, R., Roger, J.E., and Swaroop, A. (2018). Cone-rod homeobox CRX controls presynaptic active zone formation in photoreceptors of mammalian retina. Hum. Mol. Genet. *27*, 3555–3567.

Capowski, E.E., Samimi, K., Mayerl, S.J., Phillips, M.J., Pinilla, I., Howden, S.E., Saha, J., Jansen, A.D., Edwards, K.L., Jager, L.D., et al. (2019). Reproducibility and staging of 3D human retinal organoids across multiple pluripotent stem cell lines. Development *146*, dev171686.

Collin, J., Queen, R., Zerti, D., Dorgau, B., Hussain, R., Coxhead, J., Cockell, S., and Lako, M. (2019). Deconstructing retinal organoids: single cell RNA-seq reveals the cellular components of human pluripotent stem cell-derived retina. Stem Cells *37*, 593–598.

Cowan, C.S., Renner, M., De Gennaro, M., Gross-Scherf, B., Goldblum, D., Hou, Y., Munz, M., Rodrigues, T.M., Krol, J., Szikra, T., et al. (2020). Cell types of the human retina and its organoids at single-cell resolution. Cell *182*, 1623–1640.e34.

den Hollander, A.I., Roepman, R., Koenekoop, R.K., and Cremers, F.P. (2008). Leber congenital amaurosis: genes, proteins and disease mechanisms. Prog. Retin. Eye Res. *27*, 391–419.

Fadl, B.R., Brodie, S.A., Malasky, M., Boland, J.F., Kelly, M.C., Kelley, M.W., Boger, E., Fariss, R., Swaroop, A., and Campello, L. (2020). An optimized protocol for retina single-cell RNA sequencing. Mol. Vis. *26*, 705–717.

Furukawa, T., Morrow, E.M., Li, T., Davis, F.C., and Cepko, C.L. (1999). Retinopathy and attenuated circadian entrainment in Crx-deficient mice. Nat. Genet. *23*, 466–470.

Gonzalez-Cordero, A., Goh, D., Kruczek, K., Naeem, A., Fernando, M., Kleine Holthaus, S.M., Takaaki, M., Blackford, S.J.I., Kloc, M., Agundez, L., et al. (2018). Assessment of AAV vector tropisms for mouse and human pluripotent stem cell-derived rpe and photoreceptor cells. Hum. Gene Ther. *29*, 1124–1139.

Hennig, A.K., Peng, G.H., and Chen, S. (2008). Regulation of photoreceptor gene expression by Crx-associated transcription factor network. Brain Res. *1192*, 114–133.

Hoshino, A., Ratnapriya, R., Brooks, M.J., Chaitankar, V., Wilken, M.S., Zhang, C., Starostik, M.R., Gieser, L., La Torre, A., Nishio, M., et al. (2017). Molecular anatomy of the developing human retina. Dev. Cell *43*, 763–779.

Huang, L., Xiao, X., Li, S., Jia, X., Wang, P., Guo, X., and Zhang, Q. (2012). CRX variants in cone-rod dystrophy and mutation overview. Biochem. Biophys. Res. Commun. *426*, 498–503.

Hull, S., Arno, G., Plagnol, V., Chamney, S., Russell-Eggitt, I., Thompson, D., Ramsden, S.C., Black, G.C., Robson, A., Holder, G.E., et al. (2014). The phenotypic variability of retinal dystrophies associated with mutations in CRX, with report of a novel macular dystrophy phenotype. Invest. Ophthalmol. Vis. Sci. *55*, 6934–6944.

Ibrahim, M.T., Alarcon-Martinez, T., Lopez, I., Fajardo, N., Chiang, J., and Koenekoop, R.K. (2018). A complete, homozygous CRX deletion causing nullizygosity is a new genetic mechanism for Leber congenital amaurosis. Sci. Rep. *8*, 5034.

Kaya, K.D., Chen, H.Y., Brooks, M.J., Kelley, R.A., Shimada, H., Nagashima, K., de Val, N., Drinnan, C.T., Gieser, L., Kruczek, K., et al. (2019). Transcriptome-based molecular staging of human stem cell-derived retinal organoids uncovers accelerated photoreceptor differentiation by 9-cis retinal. Mol. Vis. *25*, 663–678.

Kim, S., Lowe, A., Dharmat, R., Lee, S., Owen, L.A., Wang, J., Shakoor, A., Li, Y., Morgan, D.J., Hejazi, A.A., et al. (2019). Generation, transcriptome profiling, and functional validation of cone-rich human retinal organoids. Proc. Natl. Acad. Sci. U S A *116*, 10824–10833.

Kruczek, K., and Swaroop, A. (2020). Pluripotent stem cell-derived retinal organoids for disease modeling and development of therapies. Stem Cells *38*, 1206–1215.

Kumaran, N., Moore, A.T., Weleber, R.G., and Michaelides, M. (2017). Leber congenital amaurosis/early-onset severe retinal dystrophy: clinical features, molecular genetics and therapeutic interventions. Br. J. Ophthalmol. *101*, 1147–1154.

Lane, A., Jovanovic, K., Shortall, C., Ottaviani, D., Panes, A.B., Schwarz, N., Guarascio, R., Hayes, M.J., Palfi, A., Chadderton, N., et al. (2020). Modeling and rescue of RP2 retinitis pigmentosa using iPSC-derived retinal organoids. Stem Cell Rep. *15*, 67–79.

Mao, H., James, T., Jr., Schwein, A., Shabashvili, A.E., Hauswirth, W.W., Gorbatyuk, M.S., and Lewin, A.S. (2011). AAV delivery of wild-type rhodopsin preserves retinal function in a mouse model of autosomal dominant retinitis pigmentosa. Hum. Gene Ther. *22*, 567–575.

Mitton, K.P., Swain, P.K., Chen, S., Xu, S., Zack, D.J., and Swaroop, A. (2000). The leucine zipper of NRL interacts with the CRX homeodomain. A possible mechanism of transcriptional synergy in rhodopsin regulation. J. Biol. Chem. *275*, 29794–29799.

Nichols, L.L., 2nd, Alur, R.P., Boobalan, E., Sergeev, Y.V., Caruso, R.C., Stone, E.M., Swaroop, A., Johnson, M.A., and Brooks, B.P. (2010). Two novel CRX mutant proteins causing autosomal dominant Leber congenital amaurosis interact differently with NRL. Hum. Mutat. *31*, E1472–E1483.

Phillips, M.J., Perez, E.T., Martin, J.M., Reshel, S.T., Wallace, K.A., Capowski, E.E., Singh, R., Wright, L.S., Clark, E.M., Barney, P.M., et al. (2014). Modeling human retinal development with patient-specific induced pluripotent stem cells reveals multiple roles for visual system homeobox 2. Stem Cells *32*, 1480–1492.

Rivolta, C., Berson, E.L., and Dryja, T.P. (2001). Dominant Leber congenital amaurosis, cone-rod degeneration, and retinitis pigmentosa caused by mutant versions of the transcription factor CRX. Hum. Mutat. *18*, 488–498.

Roger, J.E., Hiriyanna, A., Gotoh, N., Hao, H., Cheng, D.F., Ratnapriya, R., Kautzmann, M.A., Chang, B., and Swaroop, A. (2014). OTX2 loss causes rod differentiation defect in CRX-associated congenital blindness. J. Clin. Invest. *124*, 631–643.

Shimada, H., Lu, Q., Insinna-Kettenhofen, C., Nagashima, K., English, M.A., Semler, E.M., Mahgerefteh, J., Cideciyan, A.V., Li, T., Brooks, B.P., et al. (2017). In vitro modeling using ciliopathy-patient-derived cells reveals distinct cilia dysfunctions caused by CEP290 mutations. Cell Rep. *20*, 384–396.

Swaroop, A., Wang, Q.L., Wu, W., Cook, J., Coats, C., Xu, S., Chen, S., Zack, D.J., and Sieving, P.A. (1999). Leber congenital amaurosis caused by a homozygous mutation (R90W) in the homeodomain of the retinal transcription factor CRX: direct evidence for the involvement of CRX in the development of photoreceptor function. Hum. Mol. Genet. *8*, 299–305.

Tran, N.M., Zhang, A., Zhang, X., Huecker, J.B., Hennig, A.K., and Chen, S. (2014). Mechanistically distinct mouse models for CRX-associated retinopathy. PLoS Genet. *10*, e1004111.

Veleri, S., Lazar, C.H., Chang, B., Sieving, P.A., Banin, E., and Swaroop, A. (2015). Biology and therapy of inherited retinal degenerative disease: insights from mouse models. Dis. Model. Mech. *8*, 109–129.

Watanabe, S., Sanuki, R., Ueno, S., Koyasu, T., Hasegawa, T., and Furukawa, T. (2013). Tropisms of AAV for subretinal delivery to the neonatal mouse retina and its application for in vivo rescue of developmental photoreceptor disorders. PLoS One *8*, e54146.

Zhong, X., Gutierrez, C., Xue, T., Hampton, C., Vergara, M.N., Cao, L.H., Peters, A., Park, T.S., Zambidis, E.T., Meyer, J.S., et al. (2014). Generation of three-dimensional retinal tissue with functional photoreceptors from human iPSCs. Nat. Commun. *5*, 4047.

Stem Cell Reports, Volume *16*

Supplemental Information

Gene Therapy of Dominant *CRX*-Leber Congenital Amaurosis using Patient Stem Cell-Derived Retinal Organoids

Kamil Kruczek, Zepeng Qu, James Gentry, Benjamin R. Fadl, Linn Gieser, Suja Hiriyanna, Zachary Batz, Mugdha Samant, Ananya Samanta, Colin J. Chu, Laura Campello, Brian P. Brooks, Zhijian Wu, and Anand Swaroop

Supplemental Information

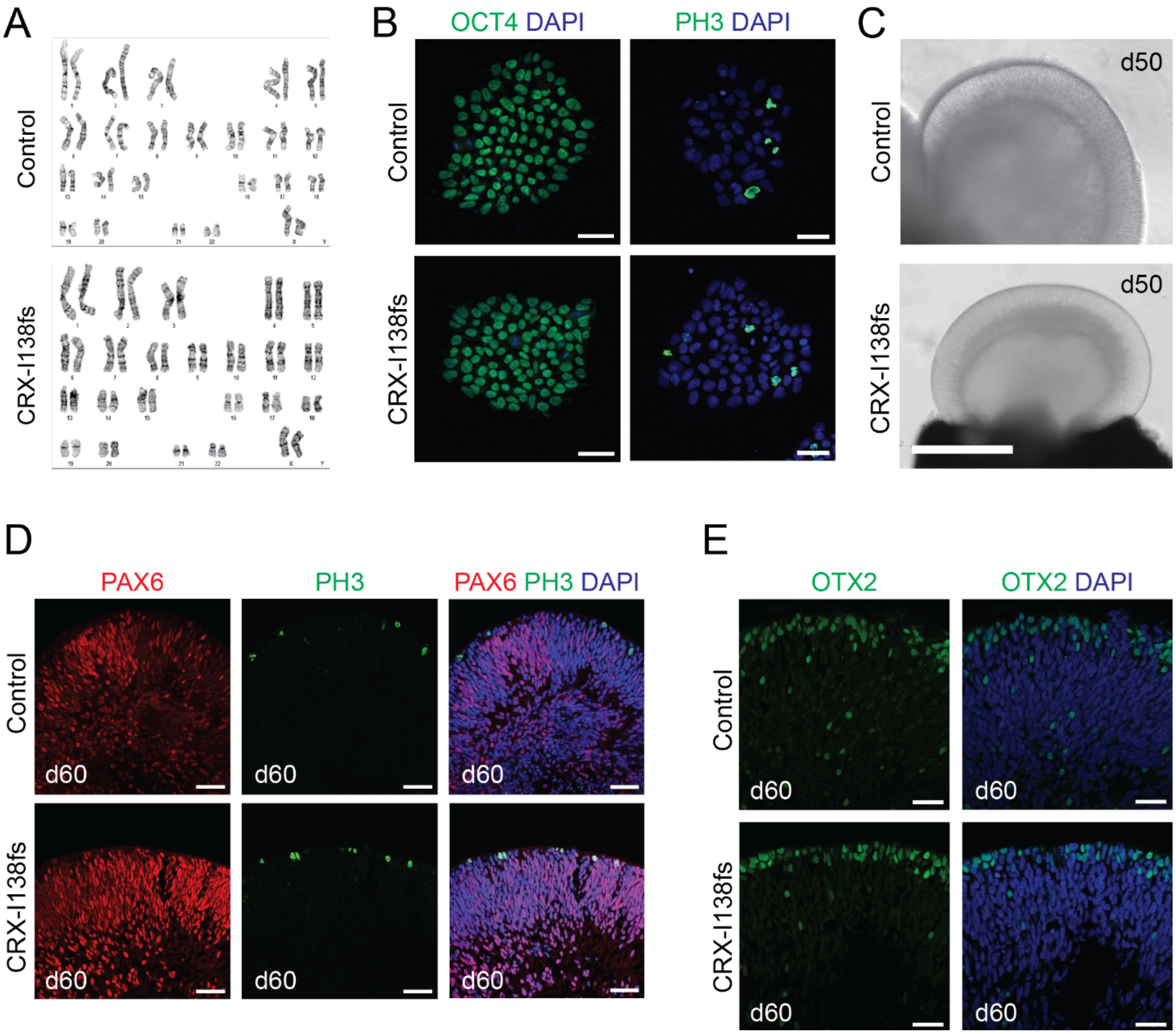

Figure S1. Differentiation of retinal organoids from control and CRX-I138fs iPSCs. Related to Figure 1.

(**A**) Representative karyograms for control and CRX-I138fs iPSC lines. (**B**) Staining of iPSCs for pluripotency (OCT4) and proliferation markers (phospho-histone 3, PH3). Scale bar, 10 µm. (**C**) Brightfield images of optic vesicle structures in control and patient organoids at differentiation day 50. Scale bar, 400 µm. (**D**) Immunostaining of organoids for early retinal marker PAX6 and proliferation marker PH3. Scale bar, 20 µm. (**E**) Immunostaining for retinal progenitor and early photoreceptor marker OTX2. Scale bar, 20 µm. Note indistinguishable patterns between control and patient organoids. Nuclei were stained with DAPI (4′,6-diamidino-2-phenylindole).

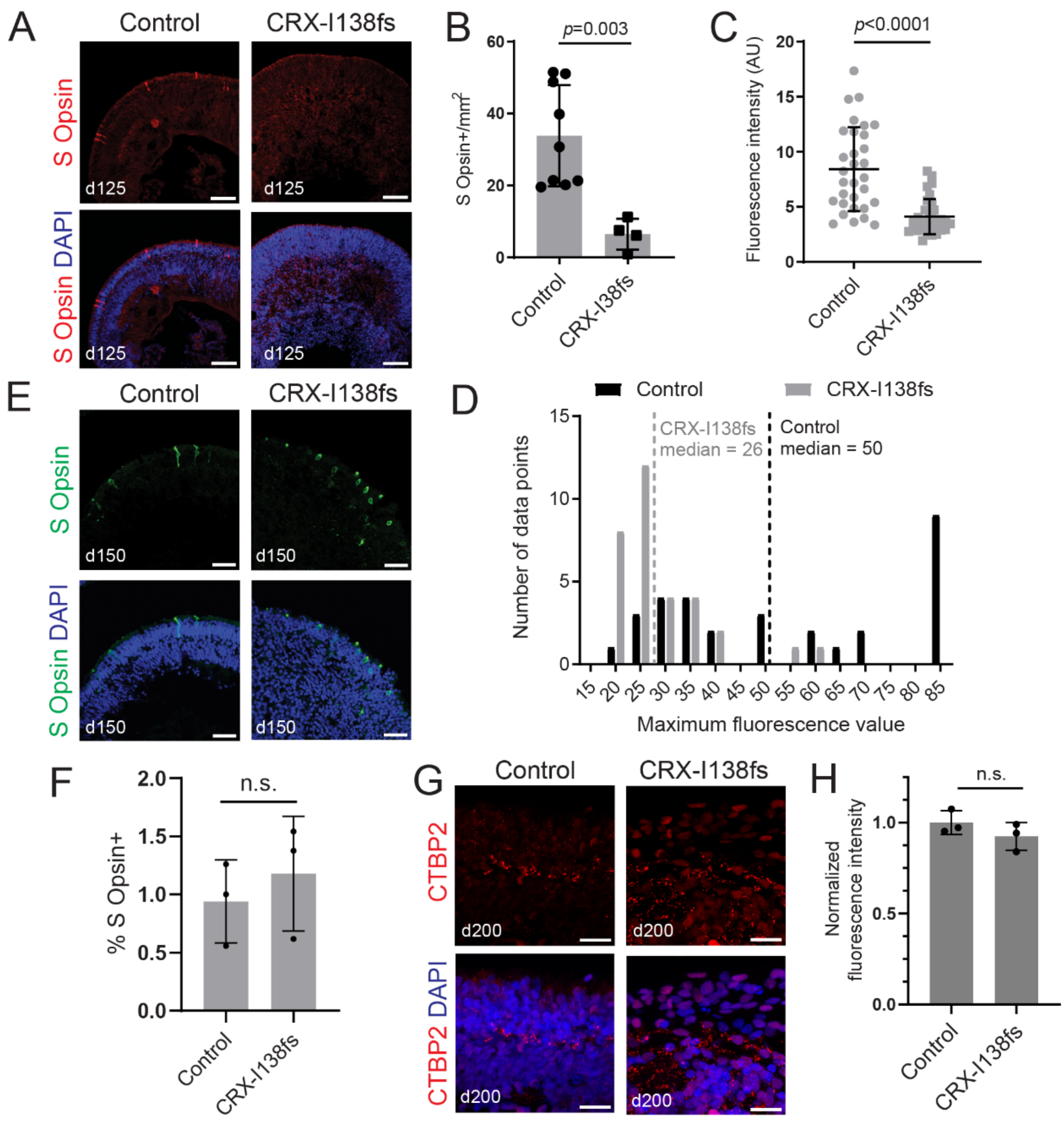

Figure S2. CRX-I138fs impairs specific aspects of photoreceptor differentiation. Related to Figure 2.

Delayed S opsin expression in CRX-I138fs organoids. (**A**) S opsin immunostaining in organoids at d125. Scale bar, 50 µm. S Opsin+ cells are mostly undetectable in the patient organoids. (**B**) Quantification of S Opsin+ cells per organoid section area at d125; control n=9, patient n=4 organoids; mean±SD. (**C**) Fluorescence intensity measurement (AU – arbitrary units) for S Opsin+ cells at d125; control n=31, patient n=32 cells; mean±SD. (**D**) Histogram of maximum fluorescence intensity values in S Opsin+ cells at d125. Control n=31, patient n=32 cells. Note the shift to lower values in patient samples. (**E**) Immunostaining for S Opsin at d150 shows induction of its expression in CRX-I138fs patient organoids by this timepoint. (**F**) Quantification of staining in (E) at d150, n=3 each group. (**G**) Immunostaining for CTBP2 (Ribeye) at d200. CTBP2-staining synaptic puncta are present in both genotypes. (**H**) Quantification of CTBP2 fluorescence intensity at d200, 3 organoids of each genotype were assessed, mean±SD plotted. Statistical significance was determined using Student's *t*-test for all quantifications; *p* values are indicated. In A, E, G: Nuclei were stained with DAPI (4′,6-diamidino-2-phenylindole).

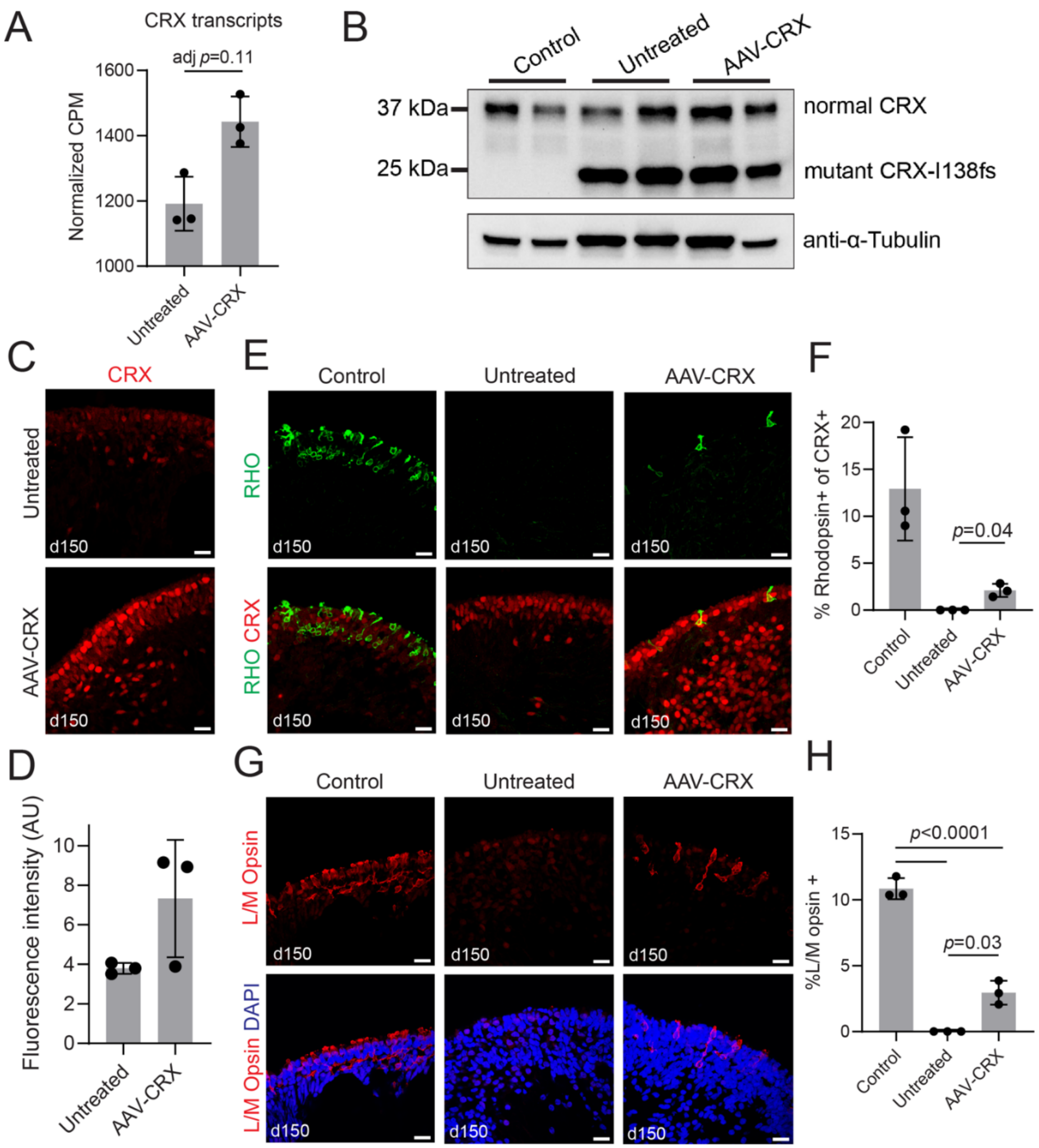

Figure S3. CRX gene augmentation in CRX-I138fs patient organoids. Related to Figure 3.

(**A**) Normalized CRX transcript counts in bulk RNA-seq data of untreated and AAV-transduced organoids at d150. (**B**) Immunoblot analysis of d200 control, untreated and AAV-treated protein samples using anti-CRX antibody. α-Tubulin was used as loading control. Molecular mass of the two CRX isoforms is indicated on the left. (**C**) Immunostaining of CRX in untreated and AAV-CRX treated organoids. Scale bar, 20 µm. (**D**) Quantification of CRX immunolabeling intensity with 3 organoids per group. (**E**) Immunostaining of Rhodopsin and CRX in control, untreated and AAV-CRX treated CRX-I138fs retinal organoids at d150, 30 days following the vector addition (at d120). (**F**) Quantification of immunostaining in (E); n=3 organoids for each group, data presented as percent of CRX+ cells. (**G**) Immunostaining of L/M Opsin of samples as in (E). Nuclei were stained with DAPI (4′,6-diamidino-2-phenylindole). (**H**) Quantification of immunostaining shown in (G), n=3 organoids per group, data presented as percent of all nuclei. Values in D,F,H represent mean ± SD with individual data points plotted. Statistical significance was determined by one-way ANOVA with Tukey's post hoc test for multiple comparisons. Significant *p* values are indicated.

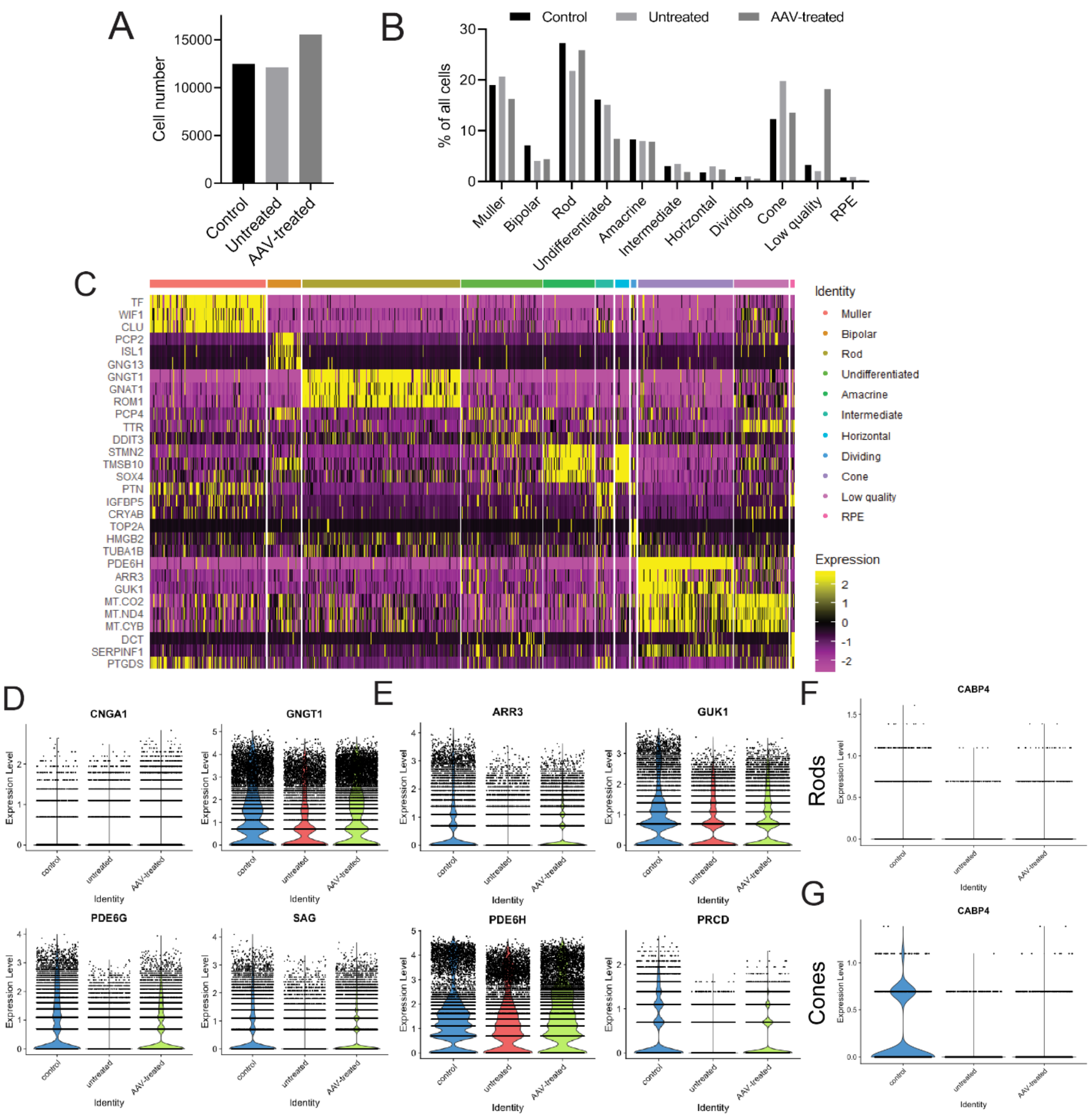

Figure S4. Cell type diversity in *CRX*-LCA retinal organoids and gene augmentation effects revealed by single cell transcriptomics. Related to Figure 5.

(**A**) Number of single cell transcriptome profiles obtained from control (12496 cells), untreated (12126 cells) and AAV-treated CRX-I138fs (15550 cells) organoids at d200. (**B**) Cell type distributions across various conditions. (**C**) Heatmap of 3 top transcripts most significantly enriched in each assigned cell class in the combined data set. The molecular markers were used to define cell types across experimental conditions. (**D**) Examples of rod-specific transcript expression rescued by AAV treatment in rod photoreceptors. For all genes adjusted p value < 0.05, Wilcoxon rank sum test with Bonferroni correction; min. percent expressed = 10% cells, min. log fold change = 0.25. (**E**) Example of cone transcripts rescued by AAV treatment. (**F,G**) Expression of CABP4, a retinal disease-associated direct transcriptional target of CRX, in rod (F) and cone (G) photoreceptors, showing a trend toward higher expression after AAV treatment.

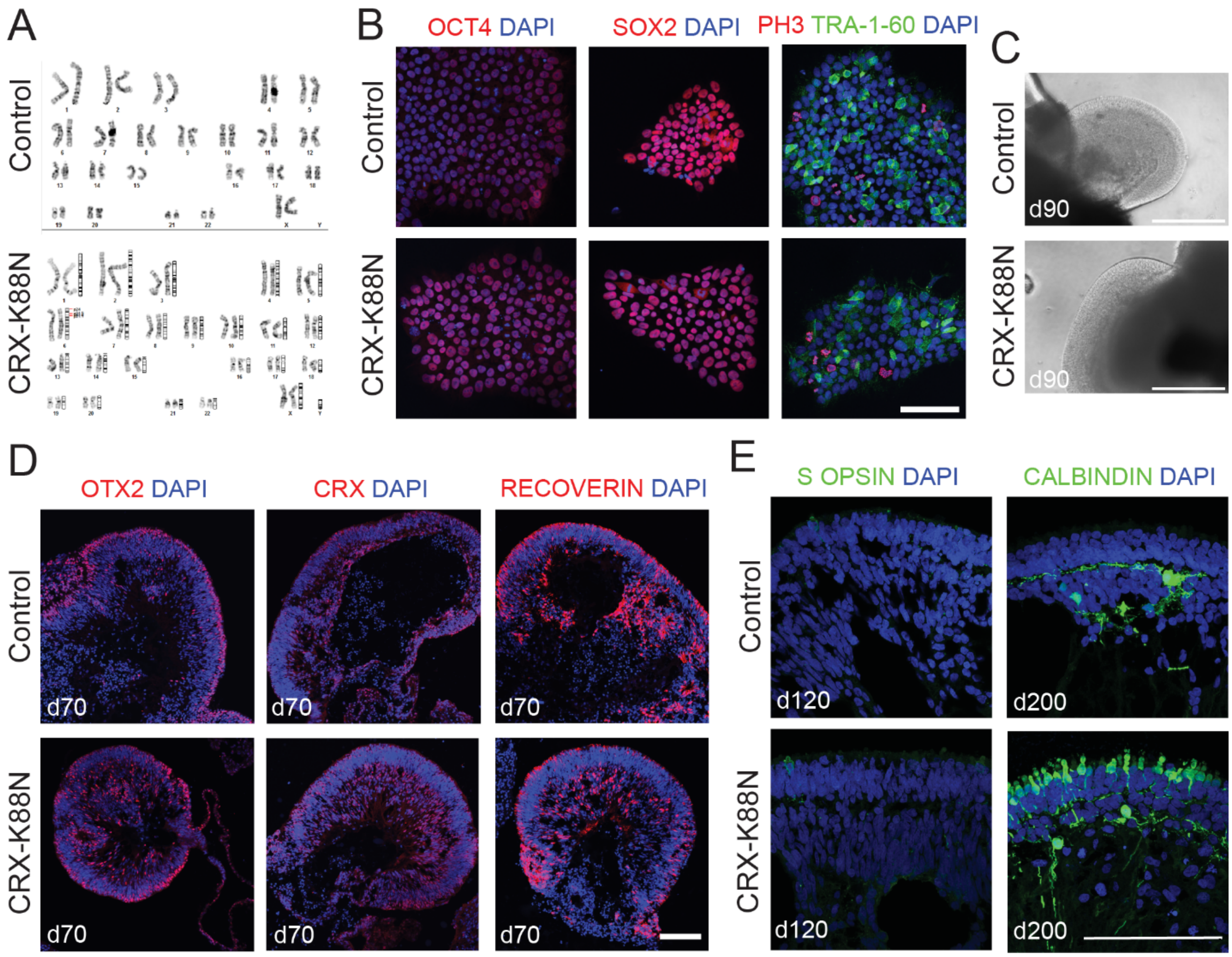

Figure S5. Differentiation of retinal organoids from CRX-K88N patient iPSCs. Related to Figure 6.

(**A**) Representative karyograms of control and CRX-K88N iPSC lines. (**B**) Immunostaining of control and CRX-K88N iPSC colonies using pluripotency (OCT4, SOX2, TRA-1-60) and proliferation (PH3) markers. (**C**) Brightfield images of organoid retinal epithelia at d90. Scale bar, 200 µm. (**D**) Immunostaining for OTX2, CRX and Recoverin in control and CRX-K88N retinal organoids at d70 of differentiation. All three markers showed similar staining between the two genotypes. (**E**) Immunostaining for S Opsin and Calbindin in control and CRX-K88N retinal organoids. Note an increased Calbindin staining in patient organoids at d200. Nuclei were stained with DAPI (4′,6-diamidino-2-phenylindole). Scale bars in B,D,E, 100 µm.

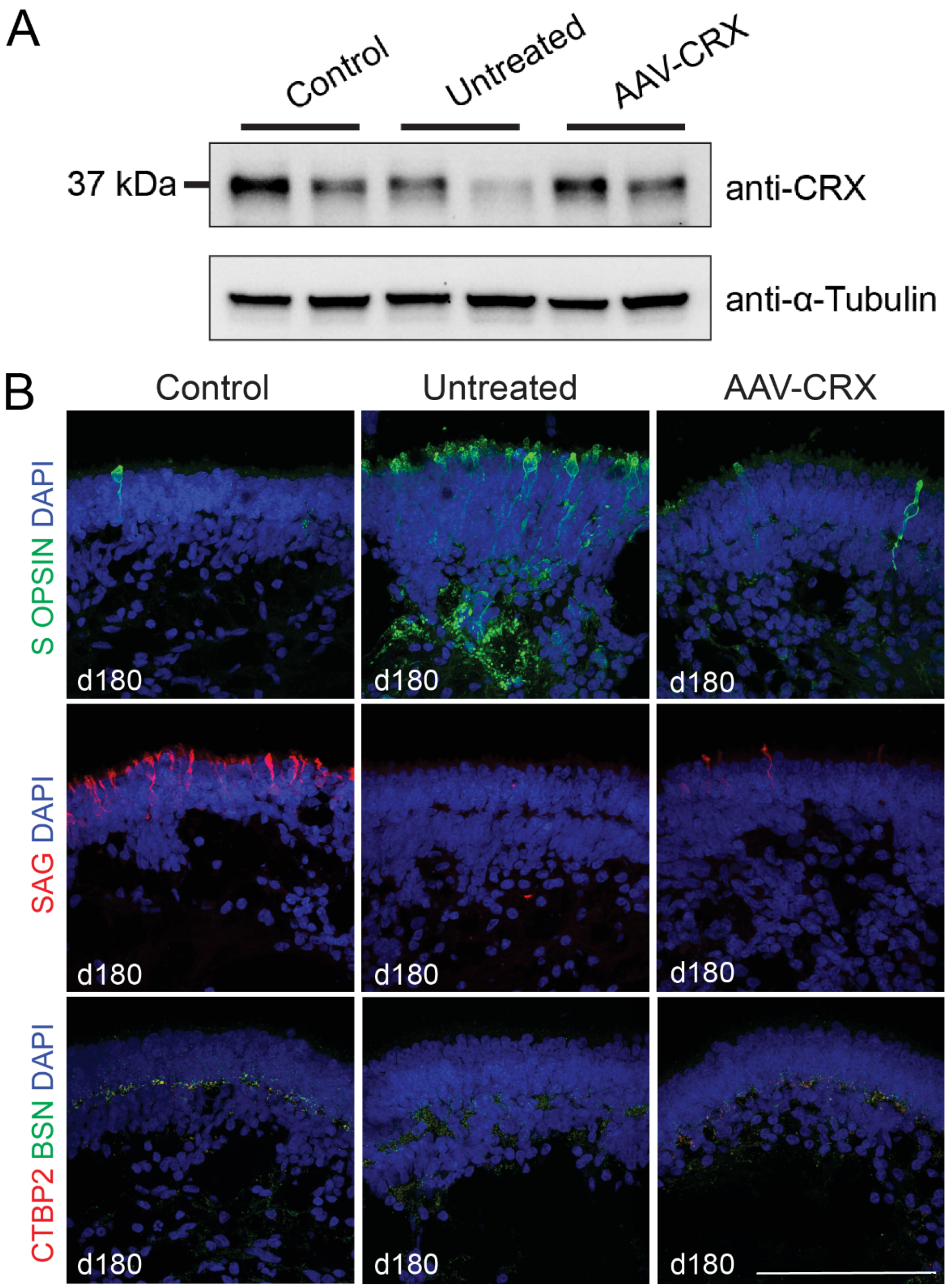

Figure S6. AAV treatment of CRX-K88N retinal organoids. Related to Figure 6.

(**A**) Immunoblot analysis of CRX protein in control, untreated and AAV-treated organoids at d200. α-tubulin was used as a loading control. Molecular mass of CRX is indicated on the left. (**B**) Immunostaining of retinal markers – S Opsin, SAG (rod Visual Arrestin) and synaptic proteins CTBP2 (Ribeye) and Bassoon (BSN) – in control, untreated and AAV-treated CRX-K88N organoids. Abnormally high S Opsin staining is reduced following AAV treatment; n=4 organoids examined in each group showed a consistent pattern. SAG expression which is undetectable in the patient organoids is modestly rescued and synaptic areas show qualitatively more staining after CRX augmentation. Nuclei were stained with DAPI. Scale bar, 100 µm.

Table S1. Study subjects. Related to all figures.

Patient ID	Disease status	Family relationship	Genotype	Protein variant	Sex	Age
NEI 001 Control	Healthy	Mother	Normal	Normal	F	36
NEI 001 Patient	*CRX*-LCA	Daughter	c.G264T	CRX-K88N	F	6
NEI 002 Control	Healthy	Mother	Normal	Normal	F	38
NEI 002 Patient	*CRX*-LCA	Daughter	c.413delT	CRX-I138fs	F	5

Table S2. IPSC line derivation and characterization. Related to all figures.

IPSC line	Derived from	Karyotype	Pluripotency markers	Mycoplasma status
NEI 001 Control B	NEI 001 Control	Normal, 46 XX	OCT4, SOX2, TRA-1-60	Negative
NEI 001 Patient A	NEI 001 Patient	Normal, 46 XX	OCT4, SOX2, TRA-1-60	Negative
NEI 002 Control A	NEI 002 Control	Normal, 46 XX	OCT4, SOX2, TRA-1-60	Negative
NEI 002 Patient B	NEI 002 Patient	Normal, 46 XX	OCT4, SOX2, TRA-1-60	Negative

Table S3. Retinal differentiation protocol. Related to all figures.

Day	Procedure
**0**	Detach the iPSCs by EDTA solution, dissociate into small clumps by pipetting a few times. Culture in suspension in E8 medium with ROCK inhibitor (final concentration: 10 µM) in ultra-low attachment dishes (9 ml in 100 mm diameter culture dish).
**1**	Add 3 ml of 1:1 NIM (composed of DMEM/F12 with Glutamax, 1% N2 supplement, 1x Minimum essential media-non essential amino acids, 2µg/ml Heparin) – ratio E8 to NIM 3:1
**2**	Add 6 ml of 1:1 NIM – ratio E8 to NIM 1:1
**3**	Collect the embryoid bodies (EBs) to a 14 ml tube and aspirate the supernatant. Suspend EBs in full 1:1 NIM (around 10 ml per dish).
**6**	Prepare Matrigel-coated dishes for day 7.
**7**	Seed the aggregates (average size of 0.22 ± 0.05 mm) onto Matrigel-coated dishes containing 1:1 NIM at an approximate density of 20 aggregates per cm^2.
**7-16**	Change the medium every 2-3 days.
**16**	Switch the medium to 3:1 NIM (composed of 3 parts DMEM, 1 part F12, 2% B27 without vitamin A, 2mM Glutamax, 1x Minimum essential media-non essential amino acids, 1% Antibiotic-antimycotic). Change medium every 2-3 days.
**16-35**	Change the medium every 2-3 days.
**28-35**	Detach horseshoe-shaped prospective neural retina domains manually with a sharpened Tungsten needle under inverted microscope. Collect retinal domains and culture in 3:1 NIM with 20 ng/ml IGF-1 in an ultra-low attachment U bottom 96-well plate.
**35-42**	Change the medium every 2-3 days.
**42**	Supplement the medium with 10% fetal bovine serum (Gibco), 100µM Taurine (Sigma) and IGF-1.
**42-63**	Change the medium every 2-3 days.
**63-91**	Use 3:1 NIM with 10% FBS, 20 ng/ml IGF-1, 100 µM taurine (Tau) and 1 µM 9-cis-retinaldehyde. Change every 2-3 days.
**91 onwards**	Lower the concentration of 9-cis-retinaldehyde to 0.5 µM. Replace every 2-3 days.

Table S4. List of primary antibodies used in the study. Related to all figures.

Antigen	Species/type	Dilution	Source	Identifier
Bassoon	Rabbit monoclonal	1:200	Cell Signaling	6897
Calbindin	Rabbit polyclonal	1:400	Millipore/Calbiochem	PC253L
CRX	Mouse monoclonal	1:100	Abnova	H00001406-M02
CTBP2	Mouse monoclonal	1:200	BD Transduction Laboratories	612044
GFP	Goat polyclonal	1:200	Rockland	600-101-215
L/M Opsin	Rabbit polyclonal	1:250	Millipore	AB5405
OCT4	Rabbit polyclonal	1:500	Abcam	ab19857
OTX1/2	Rabbit polyclonal	1:200	Abcam	ab21990
PAX6	Mouse monoclonal	1:200	DSHB	AB_528427
Peripherin2	Chicken polyclonal	1:200	Tiansen Li	n.a.
Pospho-Histone H3	Rabbit polyclonal	1:250	Cell Signaling	#9701
Recoverin	Rabbit polyclonal	1:500	Chemicon International	AB5585
Rhodopsin	Mouse monoclonal	1:500	Robert Molday	Clone 1D4
SAG	Mouse monoclonal	1:500	Abcam	ab190315
SOX2	Rabbit polyclonal	1:100	STEMGENT	09-0024
S Opsin	Rabbit polyclonal	1:250	Millipore	AB5407
TRA-1-60	Mouse monoclonal	1:200	Millipore	MAB4360
α-Tubulin	Mouse monoclonal	1:2500	Abcam	Ab7291
VSX2	Sheep polyclonal	1:200	Abcam	ab16142

Table S5. Sequence of composite human CRX promoter. Related to Figure 2.

**Composite human CRX promoter, 631 nucleotides**
CGTCGACGGGTCAGACGGCCCCTCCCTCTCTTGCTGTCATCCCTGGCTCTTCAAGCTAATGAGACCT<br>GTCCTGATTCCTCAGCCAGGCCTGTAGCCTTAATCTCTCCTAGCAGGGGGTTTGGGGGAGGGAGGA<br>GGAGAAAGAAAGGGCCCCTTATGGCTGAGACACAATGACCCAGCCACAAGGAGGGATTACCGGGG<br>AAGTGAAACAGACCCGTGTGGGACCCAGGAGCTCAGGGACATATTAATATCTAGAGAGACAGACG<br>GTCGACAGACACCAGTTAGACCTAAGGAAGGACTTCCCTGAGGAGTAGGGGCTTATGGTCACCGGC<br>AGGAGCTGGGGCCTCCCTTCCCCATCAGCCCTAATTGCCAAGATGTCATGGGGGGAAGAGGAGGGG<br>ATTAAGCAGACGGGTGCCCCTCCCCCTCCCAGCCAATGTCACCTCCTGGTGCCCAGTCGAGTCCCCC<br>ACCTTGGCCGGGATTACCCTCCGAGTTCCAGGCCATAACAAGTGACATCACTCCCGGCCCAGGCTTA<br>AAATCTCCCCACGTGAGGGGACGTGTTTCCTTCAGCCTCTGCTGTCTGGCCGCTCTGTCTAGGTCCTG<br>GGCCACGGGAGAGCCCCGTCCCTCCTTTCTGAAG

Table S6. AAV vector production protocol. Related to Figure 2.

Step	Procedures
AAV production by transient transfection	Seed HEK 293 cells in 5 roller bottles at a density of 3×10^7 cells per bottle in 300 ml DMEM medium with 10% FBS. Culture in a tissue-culture incubator at 37ºC in 5% CO_2.
	When cells reach 80% confluency add 5 ml of 1 M HEPES buffer for pH stabilization. Prepare transfection solution by mixing 150 µg of vector transgene plasmid, 150 µg of pHLP19-AAV2 capsid plasmid, 150 µg of pLAdeno5 helper plasmid with 15 ml 0.3 M $CaCl_2$ and 15 ml of 2×HBSS buffer. Mix gently by pipetting. Add the transfection solution to roller bottle immediately.
	Incubate with transfection mix 6 h to overnight at 37ºC in 5% CO_2.
	Replace medium with 100 ml of DMEM serum-free medium.
	48 h following transfection detach cells by vigorous swirling and harvest.
	Pool cells from all 5 roller bottles into one 500 ml conical tube.
	Centrifuge at 3000×*g* for 30 min. at 4ºC.
	Discard supernatant and resuspend cell pellet in 200 ml of TSM buffer. Cell pellet can either be used immediately for purification or stored at -80ºC. If stored, frozen pellet should be thawed in a water bath at 37ºC before proceeding to purification.
Isolation and purification of AAV	Homogenize cell pellet using a microfluidizer.
	In order to remove cell debris centrifuge at 3000×*g* for 30 min. and transfer supernatant into a fresh 500 ml centrifuge tube.
	Add 1M $CaCl_2$ to a final concentration of 25 mM. Mix well and leave to incubate for 10 min. at 4ºC.
	Centrifuge at 3000×*g* for 1 h. Collect supernatant and transfer into a fresh 500 ml centrifuge tube.
	Digest residual free DNA by treatment with Benzonase at 100 U/ml for 1 h at 37ºC.
	Precipitate AAV particles by adding 40% PEG8000 / 2.5 N NaCl to a final concentration of 8% PEG. Thoroughly mix, incubate for 2 h on ice.
	Centrifuge at 3000×*g* for 30 min. at 4ºC. Discard the supernatant.
	Resuspend the pellet in 25 ml of HSSE-RNase A buffer. Incubate for 30 min. at 37ºC.
	Prepare CsCl step gradient ultracentrifugation by mixing 5 ml of 1.5 g/ml CsCl to the bottom of a 38.5 ml ultracentrifuge tube, then add 8 ml of 1.3 g/ml CsCl for the middle layer. Finally add vector suspension to the top. Make sure ultracentrifuge tubes are correctly balanced before proceeding to the next step.
	Centrifuge in SW32Ti rotor for 18 h at 28,000 rpm.
	Place the ultracentrifuge tube above a halogen beam illuminator, identify and collect viral bands with a 18G needle attached to a 5 ml syringe. Transfer into a 14 ml ultracentrifuge tube for linear gradient ultracentrifugation. Fill up the tube with 1.4 g/ml CsCl.
	Centrifuge in SW40Ti rotor for 72 h at 38,000 rpm.
	Place the ultracentrifuge tube above halogen beam illuminator. Identify and collect viral band with a 18G needle attached to a 5 ml syringe.
	Dialyze overnight in a Slide-A-Lyzer cassette in Tris-buffered saline.
	Store vector solution at -80ºC until use.

Supplemental Experimental Procedures

Derivation of iPSC lines

Skin biopsies were obtained from two *CRX*-LCA pediatric patients and healthy parental controls with donor information presented Table S1. Dermal fibroblasts were reprogrammed into iPSCs using a Sendai virus-based approach. Resulting iPSC lines were of normal karyotype and free of mycoplasma contamination with details listed in Table S2.

Cell culture

Retinal organoid differentiation protocol

Differentiation was performed as described in (Kelley et al. 2020) with minor modification of maintaining dissected organoids individually in separate wells of a 96-well ultra-low attachment round-bottom plate (Corning). The detailed procedures are listed in Table S3.

Soluble factors used in retinal differentiation:

ROCK inhibitor (Y-27632 dihydrochloride, Tocris) stock 10 mM, final concentration 10 µM, dilution 1:1000.

Taurine (Sigma-Aldrich) stock 100 mM, final concentration 100 µM, dilution 1:1000.

9-cis-retinaldehyde (Sigma-Aldrich) stock solution 1 mM, final concentration 1 µM or 500 nM, dilution 1:1000 or 1:2000

IGF-1 (Gibco) stock 10 µg/ml, final concentration 20 ng/ml, dilution 1:500

Immunohistochemistry

Retinal organoids were collected using wide-bore pipette tips, washed with phosphate buffer saline (PBS; pH 7.4) and then fixed with 4% paraformaldehyde in PBS (Neuro Technologies) for at least 1 h. Organoids were then washed 3x with PBS and transferred into 15% sucrose solution until they sank to the bottom of the tube. Then the tissue was transferred into 30% sucrose solution and kept overnight. Following these dehydration steps the organoids were placed in Shandon M-1 embedding matrix (Thermo Fisher Scientific) and snap frozen in an ethanol/dry ice bath. Blocks were cryosectioned at 18 µm onto Superfrost Plus glass slides (Thermo Fisher Scientific) in a Microm HM550 cryostat (Thermo Fisher Scientific). Sections were dried before storage at -20°C. For staining, slides were rehydrated in PBS for 15 min and then blocked in 5% donkey serum (Jackson ImmunoResearch), 1% bovine serum albumin (BSA) (Sigma-Aldrich), 0.1% Triton X-100 (Sigma-Aldrich) solution in PBS for at least 1 h. Both primary and secondary antibodies were added in 1% BSA (Sigma-Aldrich), 0.1% Triton X-100 (Sigma-Aldrich) solution in PBS. List of primary antibodies used in the study is presented in Table S4. Sections were incubated with primary antibodies overnight. Slides were washed 5x with PBS, before secondary antibodies were added for 2 h. Slides were washed 3x with PBS then incubated with 4',6-diamidino-2-phenylindole (DAPI) before addition of Fluoromount-G mounting medium (SouthernBiotech) and covering with a microscopy cover glass (VWR). Samples were imaged on a Zeiss 700 confocal microscope (Zeiss) or Leica SP-8 confocal microscope (Leica Microsystems). Images were processed with ZEN Black, ZEN Blue (Zeiss), LAS X (Leica Microsystems), ImageJ and Adobe Photoshop 2020 software packages. Quantifications and fluorescence intensity measurements were performed using ImageJ software (Schroeder et al. 2020). At least three sections from three independent organoids were used for quantification.

Wholemount staining and 3D rendering: Organoids underwent fixation in a 1:4 dilution of BD Cytoperm/Cytofix buffer (BD Biosciences, 554722) for 18 h before blocking in 1x BD Perm wash (BD Biosciences) with 1% normal donkey serum (Jackson ImmunoResearch) for 12 h at room temperature. Primary antibodies (Rhodopsin, L/M Opsin, VSX2, see table above for details) were incubated at a 1:100 dilution at room temperature for 72 h. Secondary donkey anti-mouse, anti-rabbit and anti-sheep conjugated to AlexaFluor 488, 594, 647, respectively, were incubated for 4 h. Following a PBS wash, the organoids were mounted in Ce3D medium to perform tissue clearing (Li, Germain, and Gerner 2017). Imaging was performed with a Leica SP-8 confocal microscope (Leica Microsystems) and reconstructed using Imaris 9.3.0 software package (Oxford Instruments).

Bioinformatic analysis

Bulk RNA sequencing

RNA isolation and library preparation: RNA was isolated from 3 frozen organoids per sample using the QIAGEN RNeasy Kit according to manufacturer's manual (n=3 individual organoids per sample). RNA quality was assessed using the Agilent 2100 Bioanalyzer (Agilent Technologies). Samples (RIN >7) were subsequently used for library generation using the TruSeq Library Preparation Kit (Illumina Inc.). Paired-end sequencing was performed to a length of 125 bases using HiSeq2500 (Illumina Inc.). Human genome reference sequence GRCh38.p7 and Ensembl v94 annotation were used for alignment and quantitation.

Differential analysis and clustering: Transcript-level quantification was performed with kallisto v0.45.0 against Ensembl release 94 then summarized to the gene-level using tximport v1.16.0. Genes with at least 5 count per million (CPM) in all replicates of at least one group (e.g. all three replicates of the D150 control organoids) were retained for downstream analysis. Principal component analysis (PCA) was performed on normalized CPM (log2) values of TMM normalized counts (edgeR v3.20.9) (Robinson, McCarthy, and Smyth 2010). To identify differentially expressed genes (DEGs), the standard edgeR-limma-voom workflow (Ritchie et al. 2015) using empirical Bayes shrinkage of variance was used with at least a 2-fold change and a false discovery rate (FDR) ≤0.05 cutoffs. Genes identified by any of the comparisons between control and LCA model organoids at the same time point were considered DEGs.

Gene ontology analysis: GO enrichment analysis was performed using gProfileR (v 0.7.0) (Reimand et al. 2007) and clusterProfiler (v3.11) (Yu et al. 2012) packages using the Benjamini-Hochberg procedure for multiple-test correction and expressed genes as a custom background. Redundant terms were removed using the strong hierarchical filtering option. Heatmaps of GO term gene lists were generated using the log2 CPM values.

Single cell RNA sequencing

Dissociation

Dissociation performed as described in (Fadl et al. 2020). Retinal organoids were briefly washed in cold 1X HBSS, transferred to a 5 ml polypropylene round-bottom tube and dissociated using a papain-based dissociation protocol. Individual organoids were incubated in 1 ml of digestion solution containing the papain enzyme at 8°C for 1 h followed by a second incubation at 28°C for 10 min. The samples were gently mixed by inverting the tube approximately every 10 min during the 1 h incubation and after 5 min during the 10 min incubation. Following the incubation steps, the organoids, which remained morphologically intact, sank to the bottom of the tube. The supernatant of digestion solution was removed by pipetting. 700 µl of pre-warmed inactivation solution (containing papain inhibitor) was added to the organoids, mechanical trituration was carried out by slow pipetting 10-15 times with a flame polished end glass Pasteur pipette. The solution was aspirated and gently released along the tube wall until the organoids were visibly dissociated and 700 µl of chilled washing solution was layered under the cell suspension. Cells were centrifuged using a swing-bucket rotor at 200 x g for 5 min at 4°C. Supernatant was aspirated, cells resuspended in 500 µl of DPBS containing 0.04% BSA and passed through a 40 µm cell strainer (pluriSelect).

Droplet-based single-cell RNA sequencing

Single cell RNA-seq data were generated using the 10x Genomics Chromium technology. Dissociated organoids cell suspensions were loaded onto the Chromium Single Cell system using the v2 (Chromium Single Cell 3' Library & Gel Bead Kit v2) chemistry. The subsequent steps were performed according to manufacturer's instructions with minor modifications. Cell concentration and viability counts were estimated using the Cellometer Auto 2000 Cell Viability Counter (Nexcelom Bioscience). Suspensions were diluted to achieve 900-1,400 cells/µl. To account for the possibility of cell debris being falsely counted as cells, the cell count was adjusted by subtracting 200-300 cells/µl. In order to capture transcripts from approximately 10,000 cells, approximately 17,000 live cells per sample were loaded into Chromium chip following the Cell Suspension Volume Calculator Table. For every elution step in the cDNA amplification and library construction protocols, the elution volume was increased by 3 µl (Elution solution I or Buffer EB) in order to obtain a supernatant free of contaminating SPRI magnetic beads. 12 cycles were used for the cDNA amplification reaction. Libraries were quantified using the Kapa library quantification kit (Roche) and sequenced on a NovaSeq 6000 (Illumina Inc.).

Computational analysis of single cell data

Raw sequencing output was processed through Cell Ranger pipeline (v3.1.0, 10x Genomics) using *cellranger mkfastq* and *cellranger* count with default settings and the pre-built human (hg19, GRCh38) genome reference. Filtered

expression matrices were further processed using the Seurat package in R (v3.1) (Butler et al. 2018; Stuart et al. 2019) according the publisher's vignette using *sctransform* for normalization, variable gene detection and scaling (compiled October 08th, 2019). Imported data was subsequently processed using default settings for PCA dimensionality reduction, graph-based clustering, and UMAP embedding through the following functions: SCTransfrom() > RunPCA() > RunUMAP() > FindNeighbors() > FindClusters(). We used ElbowPlot() to estimate which principal components to use. Cell type identity was determined for each resulting cluster by assessing expression of known cell type-specific maker transcripts, clusters were then merged into groups reflecting broad retinal cell types.

Preparation of AAV vectors

HEK293 cells were transfected with vector plasmid and pHLP19 and pLAdeno5 helper plasmids using $CaCl_2$ method. Cell pellet was homogenized with a microfluidizer to release AAV particles. Cell debris were eliminated by centrifugation and free DNA removed by 1 h of 100U/ml bezonase treatment. AAV particles were then precipitated in 8% PEG on ice for 2 h. AAV particles pellet was collected by centrifugation and treated with RNaseA for 30 min. at 37°C. Purification of AAV was subsequently conducted using a series of ultracentrifugation steps on CsCl density gradient and dialysis. Titration was performed by qPCR. Detailed procedures are listed in Table S6.

References:

Butler, A., P. Hoffman, P. Smibert, E. Papalexi, and R. Satija. 2018. 'Integrating single-cell transcriptomic data across different conditions, technologies, and species', *Nat Biotechnol*, 36: 411-20.

Fadl, B. R., S. A. Brodie, M. Malasky, J. F. Boland, M. C. Kelly, M. W. Kelley, E. Boger, R. Fariss, A. Swaroop, and L. Campello. 2020. 'An optimized protocol for retina single-cell RNA sequencing', *Mol Vis*, 26: 705-17.

Kelley, R. A., H. Y. Chen, A. Swaroop, and T. Li. 2020. 'Accelerated Development of Rod Photoreceptors in Retinal Organoids Derived from Human Pluripotent Stem Cells by Supplementation with 9-cis Retinal', *STAR Protoc*, 1.

Li, W., R. N. Germain, and M. Y. Gerner. 2017. 'Multiplex, quantitative cellular analysis in large tissue volumes with clearing-enhanced 3D microscopy (C(e)3D)', *Proc Natl Acad Sci U S A*, 114: E7321-e30.

Reimand, J., M. Kull, H. Peterson, J. Hansen, and J. Vilo. 2007. 'g:Profiler--a web-based toolset for functional profiling of gene lists from large-scale experiments', *Nucleic Acids Res*, 35: W193-200.

Ritchie, M. E., B. Phipson, D. Wu, Y. Hu, C. W. Law, W. Shi, and G. K. Smyth. 2015. 'limma powers differential expression analyses for RNA-sequencing and microarray studies', *Nucleic Acids Res*, 43: e47.

Robinson, M. D., D. J. McCarthy, and G. K. Smyth. 2010. 'edgeR: a Bioconductor package for differential expression analysis of digital gene expression data', *Bioinformatics*, 26: 139-40.

Schroeder, A. B., E. T. A. Dobson, C. T. Rueden, P. Tomancak, F. Jug, and K. W. Eliceiri. 2020. 'The ImageJ Ecosystem: open-source software for image visualization, processing, and analysis', *Protein Sci*.

Stuart, T., A. Butler, P. Hoffman, C. Hafemeister, E. Papalexi, W. M. Mauck, 3rd, Y. Hao, M. Stoeckius, P. Smibert, and R. Satija. 2019. 'Comprehensive Integration of Single-Cell Data', *Cell*, 177: 1888-902.e21.

Yu, G., L. G. Wang, Y. Han, and Q. Y. He. 2012. 'clusterProfiler: an R package for comparing biological themes among gene clusters', *Omics*, 16: 284-7.
